# Breast cancer subtype predictors revisited: from consensus to concordance?

**DOI:** 10.1186/s12920-016-0185-6

**Published:** 2016-06-03

**Authors:** Herman MJ. Sontrop, Marcel JT. Reinders, Perry D. Moerland

**Affiliations:** Molecular Diagnostics Department, Philips Research, High Tech Campus 11, Eindhoven, 5656 AE The Netherlands; Friss Fraud and Risk Solutions, Orteliuslaan 15, Utrecht, 3528 BA The Netherlands; Delft Bioinformatics Lab, Delft University of Technology, Mekelweg 4, Delft, 2628 CD The Netherlands; Bioinformatics Laboratory, Academic Medical Center, Meibergdreef 9, Amsterdam, 1105 AZ The Netherlands

**Keywords:** Breast cancer, Subtype, Single sample predictor, Concordance, Gene expression

## Abstract

**Background:**

At the molecular level breast cancer comprises a heterogeneous set of subtypes associated with clear differences in gene expression and clinical outcomes. Single sample predictors (SSPs) are built via a two-stage approach consisting of clustering and subtype predictor construction based on the cluster labels of individual cases. SSPs have been criticized because their subtype assignments for the same samples were only moderately concordant (Cohen’s *κ*<0.6).

**Methods:**

We propose a semi-supervised approach where for five datasets, consensus sets were constructed consisting of those samples that were concordantly subtyped by a number of different predictors. Next, nine subtype predictors - three SSPs, three subtype classification models (SCMs) and three novel rule-based predictors based on the St. Gallen surrogate intrinsic subtype definitions (STGs) - were constructed on the five consensus sets and their associated consensus subtype labels. The predictors were validated on a compendium of over 4,000 uniformly preprocessed Affymetrix microarrays. Concordance between subtype predictors was assessed using Cohen’s kappa statistic.

**Results:**

In this standardized setup, subtype predictors of the same type (either SCM, SSP, or STG) but with a different gene list and/or consensus training set were associated with almost perfect levels of agreement (median *κ*>0.8). Interestingly, for a given predictor type a change in consensus set led to higher concordance than a change to another gene list. The more challenging scenario where the predictor type, gene list and training set were all different resulted in predictors with only substantial levels of concordance (median *κ*=0.74) on independent validation data.

**Conclusions:**

Our results demonstrate that for a given subtype predictor type stringent standardization of the preprocessing stage, combined with carefully devised consensus training sets, leads to predictors that show almost perfect levels of concordance. However, predictors of a different type are only substantially concordant, despite reaching almost perfect levels of concordance on training data.

**Electronic supplementary material:**

The online version of this article (doi:10.1186/s12920-016-0185-6) contains supplementary material, which is available to authorized users.

## Background

In the last decade substantial advancements have been made in our ability to probe the human transcriptome, especially by high-throughput techniques such as microarrays and more recently by next generation sequencing, i.e. RNA-seq. These techniques have deepened our understanding of complex diseases such as breast cancer [1]. Genome-wide studies have also firmly established the notion that breast cancer does not constitute a single disease at the molecular level, but comprises a heterogeneous set of subtypes, associated with striking differences in gene expression patterns, clinical outcome and response to therapy [2]. One of the most widely adopted subtyping schemes in this regard is the one introduced by Perou et al. [3], which distinguishes the subtypes luminal (subsequently divided in the subgroups A, B, and/or C), basal, HER2 and normal-like.

**Fig. 1:**
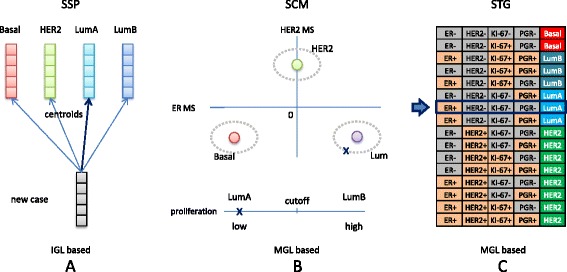
Conceptual overview subtype predictors. **a** Single sample predictor (SSP). For each subtype a centroid is computed (depicted by different colors) representing a vector of average values for each gene in the intrinsic gene list (IGL), i.e. a predetermined list of relevant genes, taken over a training set of samples assumed to be of the same subtype. In order to determine the subtype of a new case, one computes the distance to each of the centroids and assigns the new case to the subtype corresponding to the centroid that is nearest, here assumed to be the luminal A centroid, leading to the luminal A subtype. **b** Subtype classification model (SCM). Each sample is represented by three module scores (MS) calculated based on module gene lists (MGLs), i.e. the list of genes associated with a module. Training set samples are first divided into basal, HER2 and luminal subtypes by fitting a 3-component Gaussian mixture model to the ER and HER2 related module scores (*top panel, colored circles and dotted grey ovals*). Subsequently, cases of the luminal subtype are divided into two subtypes, based on their proliferation module score. Samples with a low proliferation score are assigned to the lumA (luminal A) subtype, whereas samples with a high proliferation score are assigned to the lumB (luminal B) subtype. The subtype of a new case can be determined by calculating the posterior membership probabilities under the Gaussian mixture model and selecting the subtype associated with the maximum posterior probability. In the example, the new case (*depicted with a cross*) has a high ER module score and low HER2 and proliferation module scores, leading to the luminal A subtype. **c** STG subtype predictor based on the St. Gallen surrogate intrinsic subtype definitions [14]. Over(+)/under(-)expression of clinical markers for ER, HER2, KI-67 (proliferation status) and PGR allows for 2^4^=16 distinct profiles. Here, the over/underexpression status of each marker was determined based on microarray measurements in a way similar to SCMs, i.e. via module scores. The subtype of a new case is fully determined by the over/underexpression status of the individual markers. In the example, the new case is assumed to have a high ER signaling score and low HER2, PGR and proliferation scores, leading to the luminal A subtype (*blue arrow*)

Subtype predictors have mainly been constructed via a two-stage approach [4]. In the first stage an initial grouping of samples of the same subtype is identified by hierarchical clustering, i.e. by unsupervised learning. Important ingredients of such schemes are the linkage criterion, distance measure and feature list. In the context of subtyping, the latter is often referred to as the intrinsic gene list (IGL) [3]. In the second stage a predictor is constructed based on supervised learning: cluster labels of individual cases from the first stage are used as class labels in order to train a predictor, often of the nearest centroid type. In breast cancer literature these predictors are frequently referred to as single sample predictors (SSPs) [5]. Note that once an SSP has been fitted, new cases can be subtyped without a clustering stage (Fig. 1a). The most well-known breast cancer SSPs are those by Sørlie et al. [6], Hu et al.[7] and PAM50, developed by Parker et al. [8]. In the remainder we will refer to these three predictors as the classic SSPs.

The two-stage approach towards subtype identification is, however, not without its pitfalls. Weigelt and colleagues [5] reported a low concordance between subtype assignments by the classic SSPs on four single-channel and dual-channel microarray datasets. They conclude that the classic SSPs do not reliably assign subtypes to individual patients and that therefore such identifications are not ready yet for routine clinical practice. The study was criticized by Perou et al. [9] and Sørlie et al. [10] based on bioinformatics-based technical limitations, claiming that the findings were flawed due to the use of uncentered data. In a subsequent rebuttal Weigelt et al. [11], however, showed that properly centering the data did not lead to substantial improvement of the levels of concordance. The findings by Weigelt et al. [5, 11] were corroborated by a meta-analysis of a substantially larger number of datasets from a variety of microarray platforms [12]. Herein, Haibe-Kains and colleagues reported low robustness and concordance for SSPs and proposed SCMGENE [12], a robust three-gene model based on the subtype classification model (SCM) methodology using a Gaussian mixture model on a set of module scores [13] (Fig. 1b).

From the findings of Weigelt et al. [5, 11] and Haibe-Kains et al. [12], an unsettling notion on the reliability of SSPs emerges. However, these studies have several limitations which may have negatively influenced the observed concordance. First, concordance assessments were made on data from multiple platforms, often different from the one(s) on which the SSPs had originally been constructed. Second, they used publicly available expression data that had been normalized by a variety of normalization schemes, even for data from the same platform. Third, the classic SSPs were not specifically designed to be concordant at the individual sample level [9]. Perou et al. [9] present PAM50 as a logical evolution over time in which several deliberate design changes were made compared to previous versions such as the SSPs of Sørlie and Hu. In that perspective, one could even argue that the discordance of the classic SSPs does not actually present a problem.

Here, we attempt to unify the different and sometimes conflicting views expressed in the articles by Weigelt et al. [5, 11], Perou et al. [9], Sørlie et al. [10] and Haibe-Kains et al. [12]. We do so by analyzing subtype predictors in a setup in which all predictors are specifically designed to be highly concordant at the individual sample level. For five training sets, a semi-supervised approach was used to construct corresponding consensus sets (CSs) consisting of those samples that were concordantly subtyped by a number of different predictors selected from three classes of subtype predictors: (*i*) the PAM50 SSP, (*ii*) three re-fitted SCMs and (*iii*) a novel rule-based predictor (STG) based on the surrogate intrinsic subtype definitions proposed at the 2011 St Gallen Consensus Conference [14] (Fig. 1c). For the resulting consensus samples, we argue that there is reasonable certainty regarding their subtypes. This enabled us to construct novel subtype predictors on consensus sets via supervised learning. For SSPs this may be especially advantageous as in this way a potentially unstable hierarchical clustering stage [12, 15, 16] in the predictor construction phase can be completely avoided.

We start with a comprehensive reassessment of the concordance of the classic SSPs on subtype assignments taken from the literature. We proceed with the construction of five consensus sets and construct a variety of CS-based models, which for a given subtype predictor type (SCM, SSP, or STG) mainly differ in the associated consensus training set and/or the gene list on which they were based. The CS-based predictors were subsequently applied to a large collection of validation sets. In total, we collected 22 uniformly preprocessed datasets containing over 4,000 unique hybridizations. We used this microarray compendium to assess the concordance of the classic SSPs and SCMs, and of nine novel CS-based subtype predictors: three SSPs, three SCMs, and three STGs.

## Methods

### Gene expression data

A breast cancer microrray compendium consisting of 22 datasets was constructed. The compendium comprises 4,227 breast cancer tumor samples (Table 1) and includes a set of 93 replicate array pairs. All datasets were obtained using a single measurement platform, i.e. Affymetrix. Each of the hybridizations was uniformly processed by a three-step procedure consisting of (*i*) re-normalization by frozen RMA (fRMA) [17], (*ii*) quality control and (*iii*) a robust scaling step, as described below. All the data analyzed in this study were previously published. Ethical approval was not required because no human breast tissue was acquired for this study.

**Table 1:** Overview Affymetrix compendium

ID	Dataset	Nr. of samples	Nr. of samples (QC)		Chip	Source	Reference
			Rejected	Passed			
D1	Richardson (I)	47	5	42	hgu133plus2	GSE3744	[39]
D2	Li	115	6	109	hgu133plus2	GSE19615	[40]
D3	Lu	127	3	124	hgu133plus2	GSE5460	[41]
D4	Bos	204	16	188	hgu133plus2	GSE12276	[42]
D5	Dedeurwaerder	90	7	83	hgu133plus2	GSE20711	[43]
D6	expO	353	20	333	hgu133plus2	GSE2109	[12]
D7	Kao	327	33	294	hgu133plus2	GSE20685	[44]
D8	Richardson (II)	84	9	75	hgu133plus2	GSE18864	[40]
D9	Sabatier	266	24	242	hgu133plus2	GSE21653	[45]
D10	Guedj	537	36	501	hgu133plus2	E-MTAB-365	[21]
D11	Symmans (III)	32	4	28	hgu133plus2	GSE17700	[46]
D12	Symmans (I)	298	23	275	hgu133a	GSE17705	[46]
D13	Symmans (II)	32	3	29	hgu133a	GSE17700	[46]
D14	Desmedt	198	13	185	hgu133a	GSE7390	[47]
D15	Farmer	49	3	46	hgu133a	GSE1561	[48]
D16	Schmidt	200	18	182	hgu133a	GSE11121	[49]
D17	VDX	344	29	315	hgu133a	GSE2034,GSE5327	[36, 37]
D18	Miller	251	18	233	hgu133a	GSE3494	[50]
D19	Pawitan	159	16	143	hgu133a	GSE1456	[51]
D20	Shi	278	19	259	hgu133a	GSE20194	[52, 53]
D21	MSK	99	8	91	hgu133a	GSE2603	[54, 55]
D22	UNT	137	6	131	hgu133a	GSE2990	[56, 57]
Total		4227	319	3908			

The compendium consists of data from 22 datasets measured by a single measurement platform, i.e. Affymetrix. The expression data was measured on two distinct array designs, i.e. hgu133plus2 (top 11 datasets, 2,182 samples) and hgu133a (bottom 11 datasets, 2,045 samples). We only considered the 22,215 probesets that these designs have in common, which represent all non-control probesets present on the hgu133a platform. Shared probesets are based on an identical set of probes with identical probe sequences. Remaining heterogeneity on these datasets was further reduced using frozen RMA [17] normalization and robust scaling [12] (Methods). Furthermore, an extensive quality control (QC) analysis was performed aimed at identifying (and removing) hybridizations that consistently showed indications of poor quality (Methods; Additional file 1: Section 1.2). *ID*: short dataset identifier; *Dataset*: dataset name; *Nr. of samples*: total number of available samples; *Rejected*: number of samples removed based on QC; *Passed*: total number of samples remaining after QC. In total 319 samples (7.55%) were rejected based on consistent indications of poor quality. *Chip*: array design used, i.e. hgu133plus2 or hgu133a; *Source*: the accession number under which the raw intensity data can be found at GEO [34]. Dataset D10 is available at ArrayExpress [35] (accession number E-MTAB-365); *Reference*: reference to main study. The 344 sample VDX dataset (D17) consists of the combined expression data of the 286 sample dataset by Wang et al. [36] and the 58 ER- sample dataset by Yu et al. [37]. Finally, note that the Symmans datasets (D11-D13) represent ER+ datasets. To prevent bias due to scaling of a dataset with a highly skewed subtype distribution [26, 38], datasets D12 and D13 were first concatenated to the VDX dataset and subsequently scaled as a single dataset, after which the VDX dataset was removed. Similarly, dataset D11 was combined with the expO dataset during scaling. A similar strategy was followed by Haibe-Kains et al. [12]

#### fRMA normalization

The Affymetrix compendium was normalized by fRMA using a pre-computed reference distribution for all 22,215 non-control probesets present on the hgu133a platform. Expression estimates were based on the robust weighted average mode [17] of fRMA. An extended description of the normalization procedure is provided in Additional file 1: Section 1.1.

#### Quality control

An extensive quality control (QC) analysis was performed aimed at identifying hybridizations that consistently showed indications of poor quality, either before or after normalization. The complete QC protocol, including related results, is described in Additional file 1: Section 1.2. In total 319 samples (7.5 %) were rejected based on consistent indications of poor quality. In the remaining analyses only hybridizations that passed QC were used.

### Subtype predictors

Subtype assignments to the four main subtypes on which broad agreement exists [18], i.e. basal, HER2, luminal A and luminal B, were based on three types of predictors: (*i*) SSPs, (*ii*) SCMs and (*iii*) STG subtype predictors derived from the gene expression-based quantification of estrogen receptor (ER), epidermal growth factor receptor 2 (HER2), progesterone receptor (PGR) and proliferation activity following the St. Gallen surrogate intrinsic subtype definitions (Fig. 1). A more comprehensive description of each subtype predictor type is provided in Additional file 1: Section 2.

#### Robust scaling

Normalization by fRMA does not completely remove systematic differences between datasets in the Affymetrix compendium, which were compiled over a large number of years and involve a substantial number of distinct processing sites. Therefore, for SSP-related experiments after normalization by fRMA the expression values of each dataset D1-D22 (Table 1) were robustly scaled [12], using the *genefu* package. In the scaling step, for each dataset and probeset separately, the 2.5 and 97.5 percentiles were scaled to -1 and +1, respectively. For a given SCM or STG and dataset, instead of scaling the expression data directly, we first computed the module scores on unscaled data and subsequently robustly scaled the module scores.

### Consensus sets and CS-based predictor construction and evaluation

#### Consensus sets

**Fig. 2:**
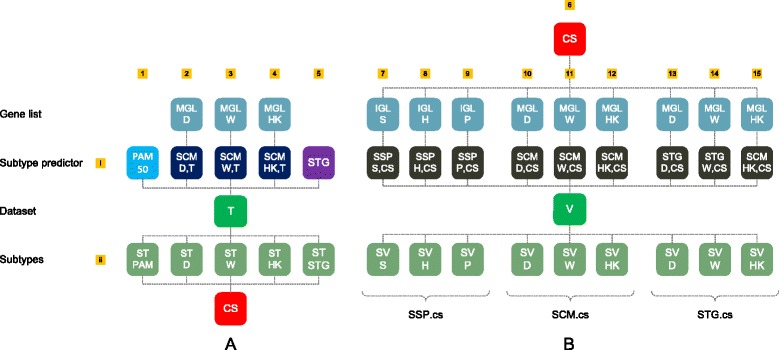
Consensus set-based predictor construction and evaluation. In panels (**a**) and (**b**), row (i) represents subtype predictor models and row (ii) corresponds to sets of predictions made by these models. Each column corresponds to a different predictor/subtype assignment set pair. **a** Consensus set construction (see Methods). For a given training set *T*, five initial sets of subtype assignments are obtained. First, the original PAM50 predictor is applied to *T*, resulting in subtype assignment set ST PAM. Next, three SCMs models are estimated on *T*, based on the MGLs D, W and HK (Additional file 1: Section 2). Here SCM X,T denotes an SCM estimated on *T*, based on MGL X. The resulting SCMs are subsequently applied to *T* resulting in three additional sets of subtype assignments, i.e. ST D, ST W, and ST HK. A final set of subtype assignments ST STG is obtained by the application of the STG predictor on the over/underexpression profile of ER, HER2, PGR and proliferation phenotypes, estimated on *T*. From the five subtype assignment sets in row (ii) a consensus set (CS) is derived consisting of those samples in *T* for which all five subtype assignments are concordant. **b** Construction and evaluation of the consensus set-based subtype predictors SSP.cs (*left*), SCM.cs (*middle*) and STG.cs (*right*), see Methods. For a given CS, three SSPs are constructed that differ only by their associated IGL S, H or P (Additional file 1: Section 2). Here SSP X,CS represents an SSP with associated IGL X, of which the centroids are estimated on CS. The SSPs are subsequently applied to a validation set V, leading to subtype assignment sets SV S, SV H and SV P, respectively. On the same CS also three SCMs are constructed, based on the MGLs D, W and HK. The resulting SCMs are subsequently applied to validation set V, yielding subtype assignment sets SV D, SV W and SV HK. Similar to SCMs, also three STG.cs predictors are constructed based on MGLs D, W and HK and applied to validation set V

In order to obtain predictors that are as concordant as possible on the individual sample level, for a given training set *T*, we only used those samples for predictor construction that were concordantly subtyped by five predictors: (*i*) the classic PAM50 SSP, (*ii*) three SCMs estimated on *T* and (*iii*) an STG predictor estimated on *T* (Additional file 1: Section 2). We refer to the set of concordantly subtyped samples as the consensus set (CS) of *T*. The complete procedure is outlined in Fig. 2a. Of the five predictors used to determine a CS, four are constructed via unsupervised learning on *T* itself. An advantage of using consensus sets for predictor construction is that SSPs, SCMs and STGs can be constructed on identical training cohorts. Furthermore, SSPs can be constructed in a supervised way, i.e. a potentially highly unstable hierarchical clustering step [12] can be avoided. Five training sets were used for consensus set construction (Table 2). In each CS all four subtypes were well represented. The stringent CS selection criteria implied a strong reduction in terms of samples available for predictor construction (median 64.0 % remaining). Note that the consensus set samples themselves can be stably identified using hierarchical clustering and lead to module scores that are reasonably bimodal (Additional file 1: Section 3).

**Table 2:** Consensus set statistics

Dataset	Chip	Nr. of samples	Nr. of samples (%)				
		after QC	CS	Basal	HER2	LumA	LumB
Bos	hgu133plus2	188	119 (63.3)	49 (41.2)	19 (16.0)	23 (19.3)	28 (23.5)
expO	hgu133plus2	333	213 (64.0)	56 (26.3)	20 (9.4)	75 (35.2)	62 (29.1)
Guedj	hgu133plus2	501	235 (46.9)	40 (17.0)	21 (8.9)	88 (37.4)	86 (36.6)
Li	hgu133plus2	109	83 (76.1)	25 (30.1)	10 (12.0)	29 (34.9)	19 (22.9)
Sabatier	hgu133plus2	242	162 (66.9)	63 (38.9)	15 (9.3)	40 (24.7)	44 (27.2)
Total		1373	812 (59.1)	233 (28.7)	85 (10.5)	255 (31.4)	239 (29.4)

Overview of the five training sets (see also Table 1) used for consensus set construction and the resulting consensus sets. Numbers in parentheses represent percentages. For CS, percentages were calculated w.r.t. the number of samples after QC; for the subtypes w.r.t. the size of the CS. The complete set of 812 consensus set samples, including subtype assignments, is available as Additional file 3

#### Construction of CS-based models

On each consensus training set, three SSPs, three SCMs and three STGs were constructed. For SSP construction we employed the IGLs related to the classic SSPs, i.e. IGL S(ørlie), H(u) and P(arker) (Additional file 1: Section 2), and used the updated probeset-to-gene mappings of Mackay et al. [19]. Similarly, for SCMs we used the module gene lists (MGLs) related to the classic SCMs, i.e. the MGL D(esmedt), W(irapati) and H(aibe-)K(ains) (Additional file 1: Section 2). For all IGLs and MGLs, in case multiple probesets mapped to the same Entrez Gene ID, the most variable probeset was selected [12]. SCMs consider three out of the four biological processes included in STGs, i.e. ER and HER2 signaling and proliferation. We therefore constructed a variety of CS-based STGs in which ER, HER2 and proliferation phenotypes were measured by the same modules as for SCMs, i.e. MGLs D, W and HK. As SCMs do not consider PGR, for this marker we always used the same single probeset module (Additional file 1: Section 2). We refer to the resulting CS-based predictors as SSP.cs, SCM.cs and STG.cs predictors, respectively. Note that CS-based predictors concordantly subtype each other’s samples (Additional file 1: Section 3). Hence, CS-based predictors were highly concordant on the individual sample level on training data. After CS-based predictor construction, all predictors were applied to a large collection of validation sets, of which the resulting subtype assignments were subsequently used in various concordance assessments. The complete procedure is outlined in Fig. 2b.

#### Concordance measure

The level of concordance between subtype assignments of two distinct subtype predictors was measured by the percentage of concordant samples (*cc*) and Cohen’s kappa statistic [20]. The range of values kappa can take is generally subdivided into five intervals that describe concordance in qualitative terms: 0–0.2 (slight), 0.21–0.4 (fair), 0.41–0.6 (moderate), 0.61–0.8 (substantial) and 0.81–1 (almost perfect). Kappa statistics were computed over all subtypes or for a specific subtype only. In the latter case, for a given subtype *s*, the complete subtype vector was transformed into a binary vector indicating whether the prediction was either *s* or not *s*. Subsequently, a contingency table was formed for which a kappa statistic was computed representing the subtype-specific kappa for subtype *s*.

## Results

**Fig. 3:**
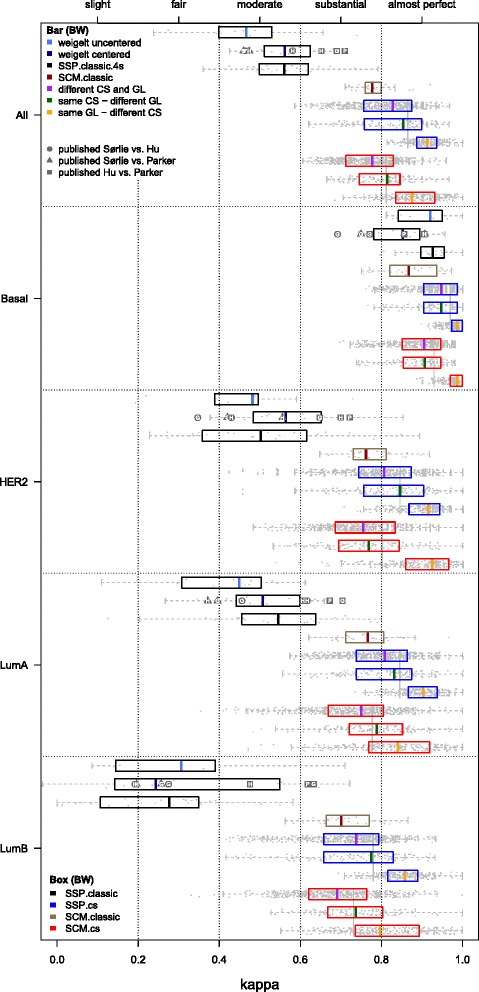
Intra-predictor concordance of SSPs and SCMs. Comparisons between predictors of the same type, e.g. the SSP of Hu vs. the SSP of Parker. The five panels show box and whisker (BW) plots for kappa statistics calculated over all subtypes and for each subtype separately, as indicated on the left hand side. Results for individual datasets are superimposed as dots. Each panel contains ten BW plots. From top to bottom these respectively indicate concordance for pairs of: (*i*) classic SSPs initially reported by Weigelt et al. [5], i.e. based on uncentered data (‘weigelt uncentered’), (*ii*) classic SSPs by Weigelt et al. [11], based on centered data (‘weigelt centered’). Estimates based on subtype assignments from the literature are superimposed as *gray symbols* with letters (see running text), (*iii*) classic SSPs without a normal-like subtype (SSP.classic.4s), (*iv*) classic SCMs (SCM.classic), (*v*) SSP.cs predictors, different CS and IGL, (*vi*) SSP.cs, same CS and different IGL, (*vii*) SSP.cs, same IGL and different CS, (*viii*) SCM.cs, different CS and MGL, (*ix*) SCM.cs, same CS and different MGL, and (*x*) SCM.cs, same MGL and different CS. Results for BWs (*iii*)-(*x*) are based on the hgu133plus2 compendium consisting of 11 datasets (2,019 samples after QC, Table 1). *Vertical gray* lines indicate kappa estimates that were pooled over all three groups of comparisons per predictor type. Top legend: type of concordance assessment indicated by the color of BW median values (indicated by a bar) (GL: gene list, IGL or MGL). Bottom legend: predictor type indicated by the color of a BW box. Numerical details of the BW plots and, highly similar, results for the analyses on all 3,908 arrays (including the hgu133a samples) are presented in Additional file 2: Tables S1 and S2

This section is divided into two parts: (*i*) concordance assessments based on a large set of previously reported classic SSP subtype assignments, (*ii*) evaluation of CS-based subtype predictors (Fig. 2) and their classic counterparts via intra- and inter-predictor concordance assessments on the Affymetrix compendium. The main results are shown in Fig. 3 that presents the central figure of this text.

### Concordance of classic SSPs on published subtype assignments

We compiled a large set of reported subtype (including normal-like) assignments for the classic SSPs based on the efforts of four research groups. The top box and whisker (BW) plot in each panel of Fig. 3 (‘weigelt uncentered’) shows the concordance levels calculated based on the subtype assignments reported by Weigelt et al. [5] (normal-like not shown) for four datasets, profiled on different array platforms, with a total of 832 samples (moderate concordance, median *κ*=0.467; Additional file 2: Table S1). Concordance levels when properly centering the data [9–11] are depicted by a second set of BW plots in Fig. 3 (‘weigelt centered’) and did not show a substantial improvement (median *κ*=0.561). Our reanalysis shows that for single-channel datasets, the effect of centering or not is in fact as large as the effect of a change to another SSP as studied by Weigelt and colleagues (Additional file 2: Table S3). From the latter observation the criticisms expressed by Perou et al. [9] and Sørlie et al. [10] appear justified.

The concordance estimates based on thousands of subtype assignments by the other three groups are superimposed over the ‘weigelt centered’ BW plots in Fig. 3 as gray symbols. Each symbol indicates a particular pair of classic SSPs (see legend), while letters indicate the origin of the subtype assignments, i.e. G: Guedj et al. [21], H: Haibe-Kains et al. [12] and P: Perou lab (https://genome.unc.edu/pubsup/breastGEO/). These findings clearly confirm the main claim by Weigelt et al. namely the lack of concordance of the classic SSPs, on a much larger number of samples. Especially the luminal B subtype was highly discordant (*κ*=0.192–0.633, Additional file 2: Table S4). In agreement with previous observations the basal subtype was most concordantly subtyped (*κ*=0.692–0.907). The highest level of overall concordance between SSPs was obtained by the Perou lab for the SSP by Hu and PAM50 (*κ*=0.710, cc=77.60 %). This is not surprising given that both SSPs were developed at the Perou lab and were mainly applied by them to data from the same dual-channel platform.

### Concordance of classic and CS-based subtype predictors on Affymetrix compendium

We next assessed the concordance of classic subtype predictors and CS-based predictors when evaluated on a large set of uniformly preprocessed validation datasets measured on Affymetrix hgu133plus2 and hgu133a microarrays (Table 1).

#### Classic SSP intra-predictor evaluation with and without a normal-like subtype

The classic SSP concordance estimates presented above were based on previously reported subtype assignments that included a normal-like subtype. We also estimated these on our hgu133plus2 compendium and again only moderate levels of agreement between classic SSPs were observed (median *κ*=0.575, median cc=70.75 %; Additional file 2: Table S2). SCM predictors, as well as our CS-based predictors, however, do not consider a normal-like subtype. The primary motivation for this choice is that currently there is no consensus whether this subtype is a genuine breast cancer subtype [21] or an artifact of breast tumor tissues having a high percentage of normal contamination in the tumor specimen [8]. Although the PAM50 predictor does include a normal-like subtype, this classification is merely considered as a quality-control measure [8]. In the remainder we do no longer consider the normal-like subtype and focus on the identification of the remaining subtypes instead. The third BW plot in each panel of Fig. 3 (SSP.classic.4s, where ‘4s’ indicates that we consider four subtypes instead of five) shows the concordance of the classic SSPs on our hgu133plus2 compendium when the normal-like centroid is removed. In this scenario we obtained similar kappa statistics for the classic SSPs as above (median *κ*=0.560, median cc=66.97 %; Additional file 2: Table S2).

#### Classic SCM intra-predictor evaluation

In our compendium the concordance of the classic SCMs was substantially higher than for the classic SSPs and in the upper range of substantial agreement (median *κ*=0.778, median cc=83.88 %; Fig. 3, Additional file 2: Table S2). Lowest concordance was observed for the luminal B subtype (median *κ*=0.701). Kappa statistics here are higher than those reported in Haibe-Kains et al. (see [12], Table 3), where concordance between the three classic SCMs reached an average *κ*=0.720 (median *κ*=0.700). In our case, however, the classic SCMs were all constructed and evaluated using data measured on a single array design, whereas Haibe-Kains et al. constructed the classic SCMs on Affymetrix data and evaluated them on a compendium that also contained many non-Affymetrix datasets. When excluding the non-Affymetrix datasets, the concordance estimates for the classic SCMs based on the subtype assignments reported by Haibe-Kains et al. [12] are highly similar to ours (Additional file 2: Table S5).

#### Strong increase in intra-predictor concordance for CS-based SSPs

The concordance levels of the consensus set-based SSPs, denoted as SSP.cs, showed a vast improvement w.r.t. the classic SSPs with kappa statistics in the range of almost perfect agreement (median *κ*=0.865, median cc=90.32 %; Additional file 2: Table S2). Note that 5 of the 11 hgu133plus2 validation sets were also used for the construction of the consensus sets and CS-based predictors. In order to avoid an upward bias of the concordance of CS-based predictors, the reported kappa statistics are strictly based on those combinations where the training set and the validation set were different. Subtype-specific performances were equally strong with median kappa statistics of 0.970, 0.846, 0.845 and 0.780 for the subtypes basal, HER2, luminal A and luminal B, respectively. In order to investigate differences due to a change in IGL or consensus set in more detail, kappa statistics were partitioned into three disjoint groups (Fig. 3, blue BW plots) for SSPs in which (*i*) both the consensus set and IGL were different, (*ii*) only the IGL was different and (*iii*) only the consensus set was different. As expected, concordance was lowest when both elements were different (median *κ*=0.828, Additional file 2: Table S1). Surprisingly, the impact of changing the IGL was larger than of a change to another consensus set (median *κ*=0.854 vs. *κ*=0.914). Consistent with previous literature, the luminal B subtype was most susceptible to changes in both the consensus set and IGL (median *κ*=0.738). However, when only the consensus set was changed, consensus for luminal B was still in the range of almost perfect agreement (median *κ*=0.857).

#### SCM.cs intra-predictor concordance

SCM predictors trained on consensus sets (SCM.cs) were also strongly concordant (median *κ*=0.812, median cc=86.67 %; Fig. 3 red BW plots; Additional file 2: Table S2), however, notably less than the SSP.cs predictors. The change to another MGL as compared to a change of consensus set showed a substantial loss in agreement (median *κ*=0.814 vs. *κ*=0.876). When both elements were changed, concordance dropped to the range of substantial agreement (median *κ*=0.778), a value equal to the overall concordance observed for the classic SCMs. Hence, SSP predictors benefit more from the consensus set construction scheme than SCMs.

#### Concordance of CS-based models and their classic counterparts

When based on the same MGL, the SCM.cs predictors showed almost perfect levels of concordance with their classic counterparts (median *κ*=0.893–0.926, median cc=92.15–94.55 %; Additional file 2: Figure S2, Table S6), with equally strong subtype-specific levels of agreement. A similarly strong level of concordance was observed between the classic PAM50 predictor and its CS-based counterpart based on IGL P (median *κ*=0.870, median cc=90.77 %). For the two oldest SSPs by Hu and Sørlie, however, only substantial (SSP Hu: median *κ*=0.775, median cc=83.95 %) and moderate (SSP Sørlie: median *κ*=0.584, median cc=70.24 %) levels of concordance were obtained with their CS-based counterparts, respectively.

#### Inter-predictor concordance of CS-based SSPs and SCMs is only substantial

**Fig. 4:**
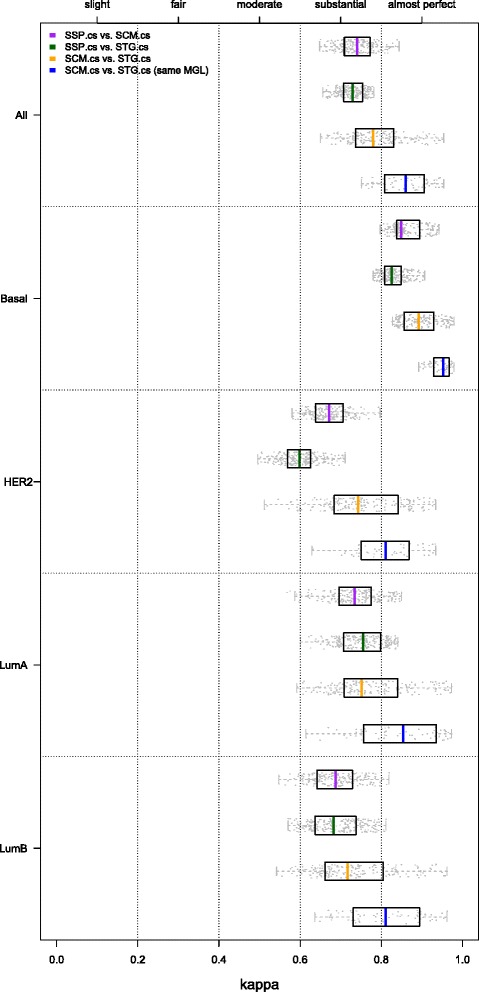
Inter-predictor concordance of CS-based models (hgu133plus2 compendium). Comparisons between predictors of different types, e.g. SCM vs. SSP. The five panels show box and whisker plots for kappa statistics calculated over all subtypes and for each each subtype separately, as indicated on the left hand side. Results for individual datasets are superimposed as dots. The upper three BW plots in each panel show the inter-predictor concordance estimates between the SSP.cs, SCM.cs and STG.cs predictors pairs, as indicated by the legend. The bottom BW plot in each panel provides the concordance estimates for SCM.cs and STG.cs predictor pairs when based on the same modules, i.e. MGLs (with exception of PGR). Results are based on the hgu133plus2 compendium. Numerical details of the BW plots are presented in Additional file 2: Table S7

Weigelt et al. [5, 11] mainly considered SSP intra-predictor concordance, i.e. concordance between predictors of the same type. Above, we showed that the intra-predictor concordances for CS-based SSPs and SCMs are in the range of almost perfect concordance. In the challenging scenario in which the consensus training set, predictor type and (as a consequence) the gene list, are different we observed only substantial levels of concordance when comparing SSP.cs and SCM.cs predictors (median *κ*=0.741; median cc=81.02 %; Fig. 4; Additional file 2: Table S7), despite the fact that the CS-based predictors showed almost perfect levels of concordance on the consensus sets themselves (Additional file 1: Section 3). In line with previous observations, only the basal subtype was identified with almost perfect levels of agreement (median *κ*=0.849), while the luminal B and HER2 subtype assignments were least concordant (median *κ*=0.688 and *κ*=0.671, respectively).

#### High inter-predictor concordance of CS-based SCMs and STGs

So far we mainly focused on SSP and SCM-based approaches. We now consider in more detail the third subtype predictor type (STG; Figs. 1 and 2b), based on the St. Gallen surrogate intrinsic subtype definitions [14]. When based on the same MGL, SCM.cs and STG.cs models show almost perfect concordance (median *κ*=0.861; median cc=89.84 %; Fig. 4, Additional file 2: Table S7). The SSP framework is conceptually quite different and overall concordance between STG.cs and SSP.cs models is indeed considerably lower (*κ*=0.729). Interestingly, the lowest concordance between STG.cs and SSP.cs models was not obtained for the luminal B subtype, but for the HER2 subtype (median *κ*=0.599). Note that even though the STG.cs predictors represent only a simple rule-based subtyping prediction scheme, fully defined by the over/underexpression status of four markers, their intra-predictor concordance was the highest of all predictors considered when based on the same MGL (Additional file 2: Figure S5).

## Discussion

A limitation of previous studies that assessed the concordance between subtype assignments [5, 9–12] is that subtype predictors were evaluated in what could be considered a worst-case scenario. Next to differences in gene lists, reported concordance statistics may have been negatively influenced by differences in the training sets used and technical heterogeneity, e.g. differences in microarray platforms, normalization and scaling strategies. Moreover, robustness and concordance of SSPs may have been negatively affected by the instability of the hierarchical clustering step [16, 19, 22, 23]. Our goal was to design an experimental setup that disentangles the various factors influencing concordance estimates, in order to obtain an improved perspective on the behaviour of modern subtype predictor schemes such as PAM50 [8] and SCMs [12, 13, 24].

### Standardization of microarray data

In contrast to the studies by Weigelt et al. [5, 11] and Haibe-Kains et al. [12], we constructed and evaluated predictors on data from a single measurement platform only, i.e. Affymetrix. Previously reported subtype assignments provide some evidence of the negative impact of technical heterogeneity (Additional file 2: Table S5), suggesting a decrease in performance when evaluating predictors in a multi-platform setup. In our study, all arrays were treated identically via a three-step procedure which involved a stringent quality control stage, renormalization of the intensity data by frozen RMA [17] and a subsequent robust scaling step. The quality of the resulting data was further supported by the high concordance obtained on replicate array pairs (Additional file 2: Figure S6, Table S8). In this standardized setup, we observed only a slight decrease in concordance when evaluating the CS-based predictors on data from another array design (hgu133a) than the one on which they were constructed (hgu133plus2), see Additional file 2: Tables S1 and S2. Note that the robust scaling step was essential for the removal of systematic technical variation between arrays from different chip designs after fRMA (Additional file 1: Figure S1). Robust scaling was also effective in datasets with a subtype distribution that is very different from the distribution used to train the subtype predictor. Recently, alternative approaches have been proposed that enable subtyping of highly skewed subtype distributions. Zhao et al. [25] introduced subgroup-specific gene centering for this purpose. Their approach is, however, limited by the need for an initial subtyping of the data, for example using ER, HER2 and PGR status as determined via immunohistochemistry. For many publicly available datasets including the ones in our Affymetrix compendium, this type of information is (partly) missing. Paquet and Hallett [26] proposed absolute intrinsic molecular subtyping (AIMS), a novel rule-based model that relates raw expression measurements of subtype-specific genes to the levels of other genes within each tumor sample. Since AIMS is truly a single sample predictor, it does not rely on a gene-centering step. An in-depth comparison of CS-based predictors and AIMS would be an interesting avenue for future research.

### Importance of consensus set

In our setup, predictor construction was performed on carefully designed training sets. Only those samples were used of which the subtypes could be concordantly identified across multiple sources, i.e. the consensus set samples (Additional file 3). The idea of a consensus set is reminiscent of the use of a core set of samples in most hierarchical clustering based subtyping approaches. From all clustered samples in general a selection is made in order to exclude samples with low correlation to each subtype. Core set selection is based on heuristics [6, 7] or statistical methods that assess the stability of a hierarchical clustering [8, 27]. Guedj et al. [21] constructed a core set by selecting those samples that were assigned to the same subtype by three different clustering methods, viz. hierarchical clustering, *k*-means and Gaussian mixture models. In contrast to these approaches, our consensus set inclusion criteria are stricter and also incorporate differences in gene lists. Since there is reasonable certainty regarding the subtype classification of the consensus set samples, we hypothesized that subtype predictors can safely be constructed on a consensus set via supervised learning. Indeed, our results show that the subtype classification of the consensus set samples themselves is highly concordant (median *κ*=0.957; Additional file 1: Table S6). Another important advantage of using consensus sets for predictor construction is that subtype predictors can be constructed on identical training sets. This allowed us to establish that the influence of a change in gene list is larger than of a change in consensus training set. Changing both elements still led to (close to) almost perfect concordance (SSP.cs: median *κ*=0.828, SCM.cs: median *κ*=0.778). For SSPs our concordance estimates are considerably higher than those reported by Weigelt et al. [5, 11] (median *κ*=0.467 before centering, median *κ*=0.561 after centering) and Haibe-Kains et al. [12] (*κ*=0.45–0.58). Concordance reported for the classic SCMs trained on the expO dataset (*κ*=0.65–0.81) [12] is also lower but more comparable to ours (SCM.cs, different MGL: median *κ*=0.814). If we consider only subtype assignments on Affymetrix cohorts, reported estimates on the concordance of the classic SCMs [12] (Additional file 2: Table S5) are highly similar to those reported here. SSPs appear to benefit more from the consensus set approach than SCMs. This is likely due to the fact that in our setup no hierarchical clustering stage was required in order to construct SSPs. For SCMs it may actually not be necessary to identify a consensus set for model fitting purposes. We observed almost perfect levels of concordance between SCM models based on consensus set samples only and those fitted on complete cohorts (median *κ*=0.954; median cc=96.67 %). In this respect SCMs are clearly superior in terms of robustness compared to SSPs constructed via hierarchical clustering.

### Factors influencing concordance

Prat et al. [28] recommend the highest level of concordance, i.e. almost perfect concordance for routine clinical use of pathology and gene-expression-based tests. Their comprehensive review shows that for virtually all currently used biomarkers in breast cancer only substantial or moderate concordance between two different methods has been reported. They state that almost perfect concordance can only be achieved by using a single platform and a standardized protocol for such tests. Our experimental setup provides an improved perspective on the factors influencing concordance between different subtyping schemes. When comparing different SSPs trained on different consensus sets, we moved from moderate concordance [5, 12] to almost perfect concordance. These results clearly illustrate the large benefit of using a standardized approach. The inter-predictor results, however, show that the choice of predictor type and associated gene lists matters. We observed large differences in the subtype assignments from predictors of different types. In the most challenging scenario in which training set, predictor type and gene list are different, we moved from moderate concordance (median *κ*=0.5) [12] to substantial concordance (median *κ*=0.741; Additional file 2: Table S7). Even though we based our conclusions on research data, we feel such discrepancies are an impediment to their incorporation into clinical practice as it is clear that the specific choice of a predictor type matters, yet it is unclear which predictor type is to be preferred. In the scenario analysed by Weigelt et al. [5] one could argue that the PAM50 predictor presents an evolution over time in which deliberate design changes were made with respect to older SSPs [9] and one may therefore claim that the observed discordance is a feature instead of a flaw. In the scenario analysed here, however, there is little room for such an interpretation as all predictors were specifically designed to be concordant on the individual sample level, while the influence of technical heterogeneity was strongly reduced. Our results also show large differences in concordance for the different subtypes. In general, the basal subtype was the only subtype which could consistently be identified with almost perfect concordance (Additional file 2: Table S1), as reported previously [5, 12].

The observed intra- and inter-predictor discordances can be explained by various factors. Our experiments clearly highlight the importance of the selected gene list, whose influence was consistently larger than the choice for a particular training set during predictor construction. Of the intrinsic subtypes the luminal B subtype was the most challenging subtype to detect concordantly. When based on the same gene list, however, we still obtained concordance levels in (or close to) the range of almost perfect agreement (SSP.cs: median *κ*=0.857, SCM.cs: median *κ*=0.797; Additional file 2: Table S1). To a certain degree, discordance between luminal A and luminal B subtype assignments may be expected if proliferation indeed forms a continuum, as suggested before [5, 12]. In most datasets considered here, however, the proliferation markers were bimodal, albeit almost never strongly (Additional file 2: Table S9). The observed lack of inter-predictor concordance can be further explained by differences in model assumptions and subtype definitions. Note that after more than a decade of molecular breast cancer subtyping, there still is no consensus on both the number and definitions of breast cancer subtypes. Especially problematic is the relation of HER2 to the other subtypes. HER2 has often been considered to belong to the ER- branch of subtypes, as is the case for the original St. Gallen surrogate intrinsic subtype definitions consisting of five subtypes [14]. In these, the luminal B subtype is split into two subtypes, i.e. luminal B (HER2+) and luminal B (HER2-) (Additional file 2: Figure S7A). In order to obtain a 4-subtype taxonomy as considered in this paper, we mapped the luminal B/HER2+ subtype to the HER2 subtype and luminal B/HER2- to the luminal B subtype. This mapping was chosen as it maximizes similarity with SCMs, in which HER2 subtype assignments are possible for both ER- and ER+ samples [13] (Additional file 2: Figure S7B). This mapping likely has a positive effect on the inter-predictor concordance of STG.cs and SCM.cs predictors. However, discordance may still arise between SCMs and STGs due to the PGR status, which is not considered by SCMs. Finally, we note that various studies have shown that within each of the intrinsic subtypes there still is considerable heterogeneity left [29–33]. Prat et al. [29] identified the claudin-low subtype, consisting of triple-negative tumors with different molecular characteristics than basal-like tumors. Lehmann and colleagues [30] described a further subdivision of triple-negative breast cancer into six stable molecular subtypes. Curtis et al. [32] proposed the 10 IntClust subtypes refining several of the intrinsic subtypes based on the integration of genomic and transcriptomic data. Molecular heterogeneity within a subtype does not imply discordance as studied in this article per se. However, this changes when it affects more than one of the intrinsic subtypes, as is the case in the St. Gallen criteria and for several of the IntClust subtypes. Therefore, in future concordance studies it is likely that considerable discordance will remain to be observed until the definitions of the molecular subtypes have been sufficiently refined. Another potential limitation of this study is that we did not evaluate the concordance of the predicted subypes with clinical parameters and their prognostic value in survival analysis. Note, however, that the high concordance of CS-based models with the classic SCMs and PAM50 suggests that they share the strong prognostic value that has been reported for classic subtype predictors [8, 12].

## Conclusions

We presented a comprehensive evaluation of SSP and SCM subtype predictors instigated by the Lancet Oncology article by Weigelt et al. [5] and subsequent reactions [9–11]. The initial study by Weigelt and colleagues reported low concordance between subtype assignments based on the classic SSPs and concluded that SSPs do not reliably assign subtypes to individual patients. In contrast, our findings show that in a carefully standardized setup via the use of consensus sets almost perfect concordance can be achieved by both SSP and SCM predictor types and for multiple gene lists. However, differences between predictor types, gene lists and training datasets combined result in subtype assignments that only show substantial levels of agreement. Prospective clinical trials are needed to go beyond the concordance issues investigated in this paper and to determine which subtype predictor is most relevant for guiding treatment choice for an individual patient.

## Additional files

Additional file 1 Supplementary methods and results. Comprehensive description of normalization and quality control of the gene expression data, different subtype predictors, and characterization of consensus set samples. (PDF 10854 kb)

Additional file 2 Supplementary figures and tables. (PDF 543 kb)

Additional file 3 Complete set of 812 consensus set samples, including subtype assignments. (XLSX 30 kb)
