# Erratum to: Breast cancer subtype predictors revisited: from consensus to concordance?

**DOI:** 10.1186/s12920-016-0209-2

**Published:** 2016-07-14

**Authors:** Herman M. J. Sontrop, Marcel J. T. Reinders, Perry D. Moerland

**Affiliations:** Molecular Diagnostics Department, Philips Research, High Tech Campus 11, Eindhoven, 5656 AE The Netherlands; Friss Fraud and Risk Solutions, Orteliuslaan 15, Utrecht, 3528 BA The Netherlands; Delft Bioinformatics Lab, Delft University of Technology, Mekelweg 4, Delft, 2628 CD The Netherlands; Bioinformatics Laboratory, Academic Medical Center, Meibergdreef 9, Amsterdam, 1105 AZ The Netherlands

## Erratum

Following the publication of the article in *BMC Medical Genomics* [1], it was reported that the initials in the author names were incorrectly captured as a part of the surname in the metadata. Please find the correct names below:

Given names: Herman M.J.

Surname: Sontrop

Given names: Marcel J.T.

Surname: Reinders

Given names: Perry D.

Surname: Moerland

We apologize for the inconvenience this may have caused.
