# Supplementary material for: Breast cancer subtype predictors revisited: from consensus to concordance?
Source: BMC Med Genomics. 2016 Jun 3;9:26. doi: 10.1186/s12920-016-0185-6 (PMC4893290; doi:10.1186/s12920-016-0185-6)
Supplement: Additional file 1 — Supplementary methods and results. Comprehensive description of normalization and quality control of the gene expression data, different subtype predictors, and characterization of consensus set samples. (PDF 10854 kb) [file 12920_2016_185_MOESM1_ESM.pdf]

Additional File 1: Supplementary Methods and Results

Breast Cancer Subtype Predictors Revisited: From Consensus to Concordance?

Herman M.J. Sontrop[1,2], Marcel J.T. Reinders[3], Perry D. Moerland[4,*]

1 Molecular Diagnostics Department, Philips Research, High Tech Campus 11, 5656 AE Eindhoven, The Netherlands

2 Friss Fraud and Risk Solutions, Orteliuslaan 15, 3528 BA, Utrecht, The Netherlands

3 Delft Bioinformatics Lab, Delft University of Technology, Mekelweg 4, 2628 CD Delft, The Netherlands

4 Bioinformatics Laboratory, Academic Medical Center, Meibergdreef 9, 1105 AZ Amsterdam, The Netherlands

∗ Corresponding author, e-mail: p.d.moerland@amc.uva.nl, phone: ++31 205666945

Contents

1 Gene expression data **2**
1.1 Normalization . . . 2
1.2 Quality control . . . 3

2 Subtype predictors **5**
2.1 SSP: single sample predictor . . . 5
2.2 SCM: subtype classification model . . . 5
2.3 STG: predictor based on St. Gallen surrogate intrinsic subtypes . . . 7

3 Consensus sets **7**
3.1 Consensus set subtype identification by hierarchical clustering . . . 7
3.2 Bimodality status of individual modules . . . 8
3.3 Concordance of CS-based predictors on consensus sets . . . 9

1 Gene expression data

For the construction and evaluation of the consensus set-driven subtype predictors only high-quality Affymetrix arrays were used. This section gives a detailed description of the normalization and quality control (QC) stages used to process and filter these hybridizations. All analyses were performed using R/Bioconductor packages.

1.1 Normalization

In order to make the expression data as comparable as possible, we (re)normalized the Affymetrix datasets by a modified version of the RMA methodology, known as frozen RMA (fRMA) [1]. This methodology allows one to normalize the intensity data of different arrays individually or in small batches and then combine the data for analysis. In particular, estimates of probe-specific effects and variances are pre-computed and frozen [1]. Another important distinction between default RMA and fRMA is the estimation of the reference distribution. In fRMA the reference distribution is not estimated from the data itself, but a pre-computed reference distribution is employed. Frozen RMA has the same logistical advantage as single chip models, in that it enables normalizing arrays one by one, while still having the benefits of a multi-chip normalization scheme. Our Affymetrix compendium involved two distinct array designs, i.e. hgu133plus2 and hgu133a arrays. We only considered the 22,215 probesets these designs have in common, which represent all non-control probesets present on the hgu133a platform. In order to utilize the common probesets, the hgu133plus2 arrays were first converted to the hgu133a platform using the function *convertPlatform* from the *frma* package. We then masked all control probesets in the arrays and in the *hgu133afrmavecs* object containing the frozen parameters, resulting in the desired 22,215 probesets. In this way all Affymetrix arrays could be normalised using a single reference distribution, i.e. the Affymetrix hgu133a reference distribution, as constructed by McCall et al. based on 1,000 samples originating from 200 distinct studies [2]. We ran *frma* in robust weighted average mode [1].

Frozen RMA mainly addresses batch effects at probe level. fRMA-normalized data may therefore still contain batch effects at probeset level. Our Affymetrix compendium indeed showed clear evidence of systematic technical variation between arrays from different chip designs after fRMA (Figure S1). This effect was removed via a robust scaling step (Methods, main text). A drawback of our approach is the loss of some hgu133plus2 probesets that are part of the gene list of certain subtype predictors. Some of these are Affymetrix control probesets which, interestingly, are included in the PAM50 gene list.

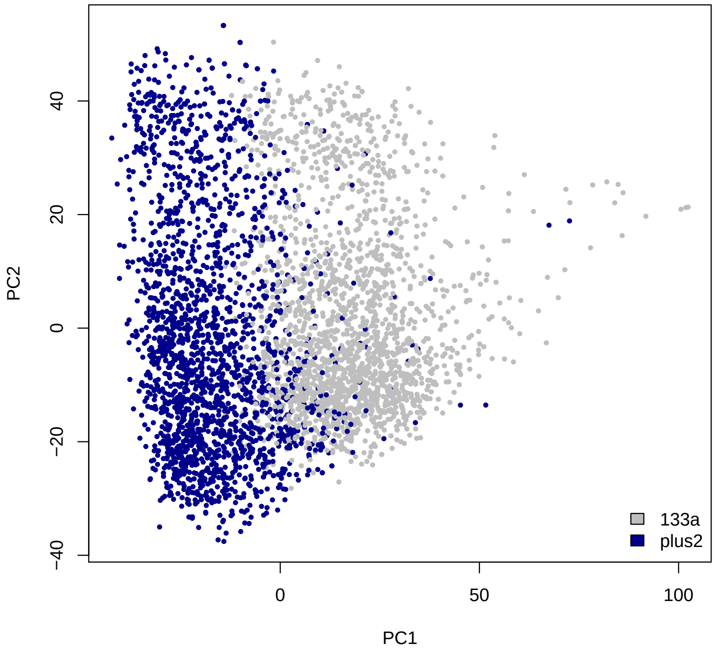

Figure S1. Principal component analysis of fRMA-normalized data (combined hgu133plus2 and hgu133a compendium). Principal component (PC) analysis plot of the fRMA-normalized expression data from our Affymetrix compendium. Expression data originated from two chip designs, i.e. hgu133plus2 and hgu133a. In order to reduce systematic technical variation we used the frozen RMA methodology in which both array designs were normalized via a single reference distribution. A set of 3,400 genes related to breast cancer subtyping was used to estimate the principal components. This set corresponds to the union of all genes contained in the gene lists of the classic SSPs, classic SCMs and the CIT subtyping scheme of Guedj et al. [3] for which probesets are present on the Affymetrix hgu133a design.

1.2 Quality control

Poor hybridizations can have a negative impact on performance [4]. As we used datasets related to a substantial collection of high-quality publications, one may reasonably expect these hybridizations had passed quality control. However, after a preliminary QC inspection a sizable number of arrays appeared to be problematic for one or more well established QC control indicators. Figure S2 provides several examples of problematic arrays encountered in our compendium. To ensure all hybridizations were of sufficient quality, an extensive QC analysis was performed aimed at identifying hybridizations that consistently showed indications of poor quality, either before or after normalization. The QC protocol we followed was based on six QC indicators: $Q = \{\text{RLE, NUSE, heatmap, boxplot, MA-plot, GNUSE}\}$. The first five represent well established QC indicators [4]. The GNUSE statistic was introduced by McCall et al. [5] and is an fRMA-based single chip alternative to the multi-chip NUSE QC statistic [6]. The NUSE, GNUSE and RLE QC indicators provide diagnostic information before normalization, while the remaining indicators provide information after normalization. All QC statistics with the exception of GNUSE were computed using the *arrayQualityMetrics* package, while GNUSE values were computed using the *frma* package. For a given QC indicator q and array i we used *arrayQualityMetrics* to obtain a series of QC scores and thresholds by repeatedly analyzing array i in the presence of B randomly selected arrays from the same dataset. Higher scores reflect arrays of potentially poor quality, while scores higher than the threshold are considered outlier arrays. For a given array i and QC indicator $q \in Q$, let $S^q_{i,r}$ and τ^q_r be the QC score and

threshold, respectively, as determined by *arrayQualityMetrics* at repeat r. Then, an array was rejected if it was considered an outlier in at least half of the QC repeats in which it was included. That is, array i was rejected based on QC if there exists a $q' \in Q$ for which we have

$$\sum_{r=1}^{R} I_{i,r}^{q'} \geq R/2$$

where $I_{i,r}^{q}$ is an indicator variable that equals 1 if $S_{i,r}^{q} > \tau_r^q$ and 0 otherwise and R is the number of repeats.

We ran the complete QC protocol on all 4,227 Affymetrix hybridizations part of our compendium. Arrays from different datasets and array designs were processed separately, with a QC batch size of $B = 30$ and $R = 10$ repeats. Hence, for each array and QC indicator we obtained 10 QC scores. In total 7.55% of the arrays (319 out of 4,227) were removed based on QC; 250 arrays (5.91%) showed consistent indications of poor quality prior to normalization and 182 (4.31%) after normalization; 2.67% (113 out of 4,227) of the arrays considered showed consistent indications of poor quality both before and after normalization. Table 1 in the main text provides an overview of the QC results per dataset.

A visualization of all computed QC statistics for each dataset is provided on pages 12-23. For each array and QC indicator separately, a box and whisker plot is shown depicting the distribution of the various QC scores associated with each array. For each QC indicator a separate row is used. For reference the QC overview figures also include several other often used Affymetrix QC indicators, i.e. average background, percentage present, and scaling factor. These, however, were not used to filter the arrays. The centered string in the top row shows the name of the dataset, the total number of arrays and the total number of arrays rejected based on QC, taken over all QC indicators. Rejected arrays are indicated by vertical dashed red lines, see Table S7 for a detailed overview. A short ID is used to indicate an array, the full name is available in Table S7. For some datasets additional information was available on the processing groups [7], e.g. the research institute in which the hybridizations were performed. In those instances QC batches were confined to include arrays from the same processing group only, even if this implied a batch size smaller than $B = 30$. Distinct processing groups are separated by green vertical lines and results are displayed per processing group. Within each processing group arrays are ordered by their median RLE score. Horizontal blue lines indicate the median QC thresholds. The box and whisker plots clearly illustrate the variability of the QC statistics, which was the main reason to design the resampling-based QC protocol described above.

x	y	ID	Dataset	Chip	GSM
1	1	771	Pawitan	hgu133a	GSM107151
1	2	1051	Schmidt	hgu133a	GSM282572
1	3	760	Pawitan	hgu133a	GSM107140
1	4	813	Pawitan	hgu133a	GSM107193.
2	1	708	Pawitan	hgu133a	GSM107087
2	2	670	MSK	hgu133a	GSM50110
2	3	1813	Wang	hgu133a	GSM36861
2	4	2343	Bos	hgu133plus2	GSM308459
3	1	415	Miller	hgu133a	GSM79350
3	2	1648	Symmans (II)	hgu133a	GSM441336
3	3	1564	Symmans (I)	hgu133a	GSM441858
3	4	4421	Sabatier	hgu133plus2	GSM540319_15744_T7
4	1	4426	Sabatier	hgu133plus2	GSM540324_16325_T56
4	2	1845	Wang	hgu133a	GSM36966
4	3	1218	Shi	hgu133a	GSM505494
4	4	163	Desmedt	hgu133a	GSM177952

Table S1. Details on the 16 poor quality arrays from Figure S2. x, y: coordinates of the examples, e.g. top left chip pseudo-image: x = 1, y = 1, bottom right: x = 4, y = 4; ID: short ID used in the QC overview figures on pages 12-23 and in Table S7; GSM: accession number in GEO [8].

2 Subtype predictors

This section provides a comprehensive description and references to the literature for the different types of subtype predictors used in the main manuscript.

2.1 SSP: single sample predictor

The classic single sample predictors are nearest centroid predictors, that is, prototype-driven classification rules that are completely defined by a set of centroids and a suitable distance function (Figure 1A, main text) [9]. In line with previously described SSP schemes [10, 11], we used the Spearman rank correlation distance measure. SSPs were constructed using the intrinsic gene lists (IGLs) related to the classic SSPs. We refer to the IGLs of the SSPs by Sørlie et al. [12], Hu et al. [10] and Parker et al. [11] as the IGL S, H and P, respectively. For the classic SSPs we used the following functions from the *genefu* package: *ssp2003.robust* (SSP Sørlie), *ssp2006.robust* (SSP Hu) and *pam50.robust* (SSP Parker).

2.2 SCM: subtype classification model

As an alternative to SSPs, Desmedt et al. [13] proposed a biology-inspired module-driven approach referred to as subtype classification models [14] (Figure 1B, main text). Module scores are calculated for three modules that reflect the activity of several key biological processes: (*i*) estrogen receptor signaling, (*ii*) HER2 signaling and (*iii*) proliferation. Three SCMs have been published previously, based on the same set of prototypes: the SCM by Desmedt et al. [13], the SCM by Wirapati et al. [15] and more recently the SCM by Haibe-Kains et al. [14], also known as SCMGENE. We refer to these as the classic SCMs. In addition, for a given SCM we refer to the list of genes associated with a module as the module gene list (MGL). The latter can be thought of as the SCM equivalent of an IGL. We refer to the MGLs corresponding to the SCMs by Desmedt et al. [13], Wirapati et al. [15] and Haibe-Kains et al. [14] as the MGLs D, W and HK, respectively. For the classic SCMs we used the following functions from the *genefu* package: *scmod1.robust* (SCM D), *scmod2.robust* (SCM W) and *scmgene.robust* (SCM HK).

For SCM.cs we used the *subtype.cluster* function in the Bioconductor package *genefu*, which for a given consensus training set and MGL computes the module scores and estimates the parameters of the

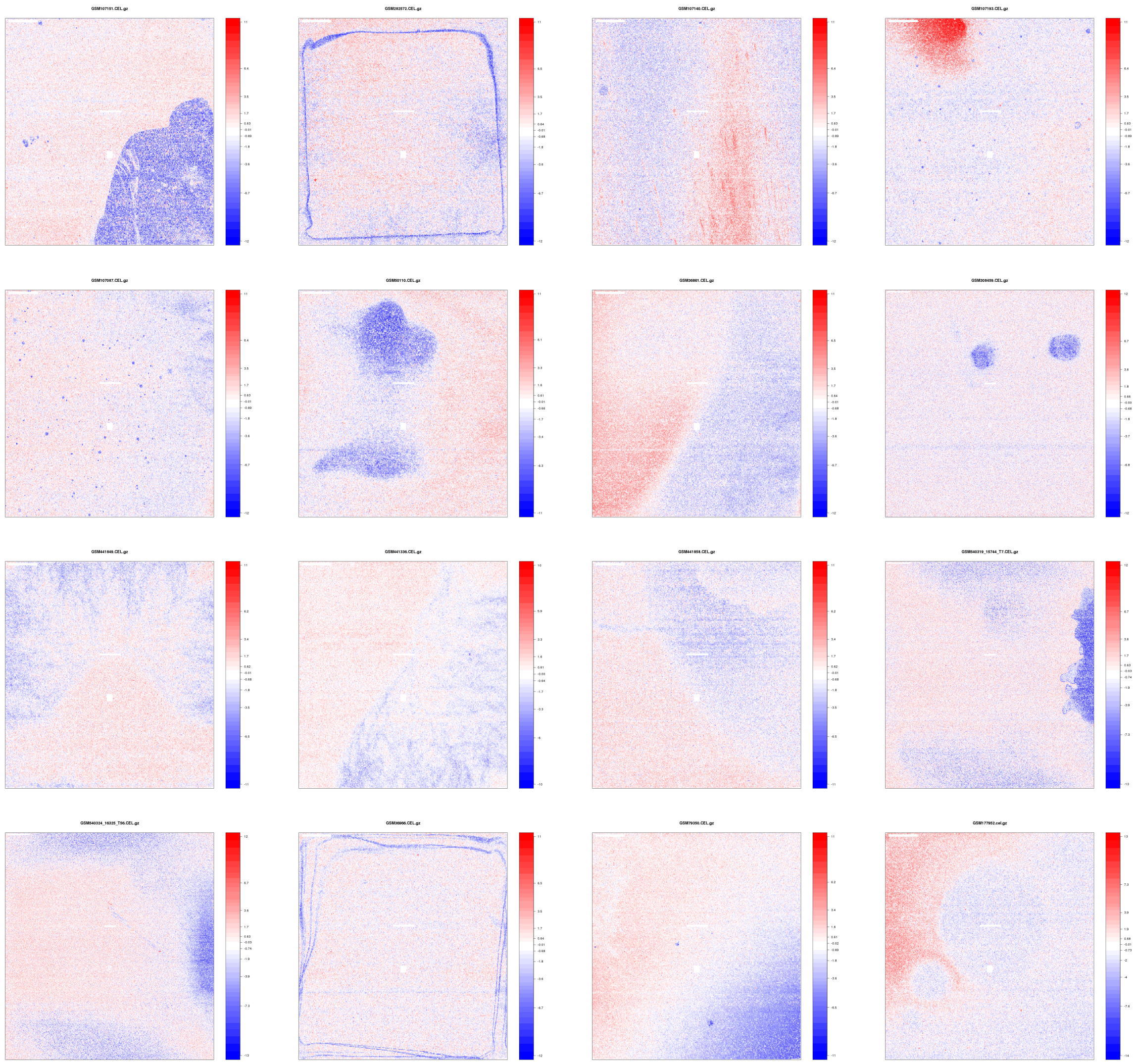

Figure S2. Chip pseudo-images for 16 examples of arrays with consistent indications of poor quality. Details are provided in Table S1.

Probeset	HUGO gene symbol	Entrez Gene ID
202095_s_at	BIRC5	332
202589_at	TYMS	7298
202870_s_at	CDC20	991
202954_at	UBE2C	11065
209773_s_at	RRM2	6241
214710_s_at	CCNB1	891

Table S2. STG proliferation module. The module composition of the 6-gene proliferation module was based on the intersection of all genes in the AURKA proliferation modules by Desmedt [13] and Wirapati [15] retrieved from the *genefu* package and the 11-gene proliferation signature proposed by Nielsen et al. [18]. The latter signature consists of the HUGO gene symbol entries: CCNB1, UBE2C, BIRC5, KNTC2, CDC20, PTTG1, RRM2, MKI67, TYMS, CEP55, CDCA1. All probesets had a weight of +1 in the calculation of the module score.

associated mixture model.

2.3 STG: predictor based on St. Gallen surrogate intrinsic subtypes

In this study, we developed a rule-based predictor (STG) derived from the St. Gallen surrogate intrinsic subtype definitions which are based on clinical markers of ER, HER2, PGR and KI-67 (proliferation) status [16]. An STG is fully defined by the over/underexpression status of the markers, which allows for 16 distinct profiles (Figure 1C, main text). Over/underexpression status of the four markers was determined by considering module scores. The ER, HER2 and PGR modules consisted of a single probeset. These correspond to the probesets previously suggested for these processes [17], and for ER and HER2 are identical to those used by SCMGENE. The proliferation module was based on the intersection of all genes in the AURKA proliferation modules by Desmedt and Wirapati and the 11-gene proliferation signature proposed by Nielsen et al. [18]. This resulted in a 6-gene proliferation module (Table S2). For each marker and training set separately, over/underexpression was estimated by fitting a 2-component Gaussian mixture model on the module scores. For each component i, let u_i, σ_i^2 and w_i be the estimated mean, variance and mixing proportion, respectively. Assuming equal variances, the following cutoff can be used to determine the actual over/underexpression status for a new case:

$$c = \frac{\sigma^2 \log(\frac{w_2}{w_1}) + \frac{1}{2}(u_1^2 - u_2^2)}{u_1 - u_2}.$$

Cases with a module score larger than or equal to c were considered overexpressed, while the others were considered underexpressed.

3 Consensus sets

This section gives an overview of a number of additional experiments, characterizing the consensus set samples in more detail.

3.1 Consensus set subtype identification by hierarchical clustering

In breast cancer literature SSP construction has almost always been linked to unsupervised learning via hierarchical clustering (HC) [3,10–12]. Instability of hierarchical clustering is a well-known problem [19,20]. Haibe-Kains et al. [14] reported very low levels of concordance for HC-based SSP predictors when clustering complete sample cohorts. We investigated to what extent the subtype labels of the consensus sets could have been identified by HC alone and to what degree their identification is influenced by the presence of additional samples during clustering. Importantly, for any given dataset concordance was always measured

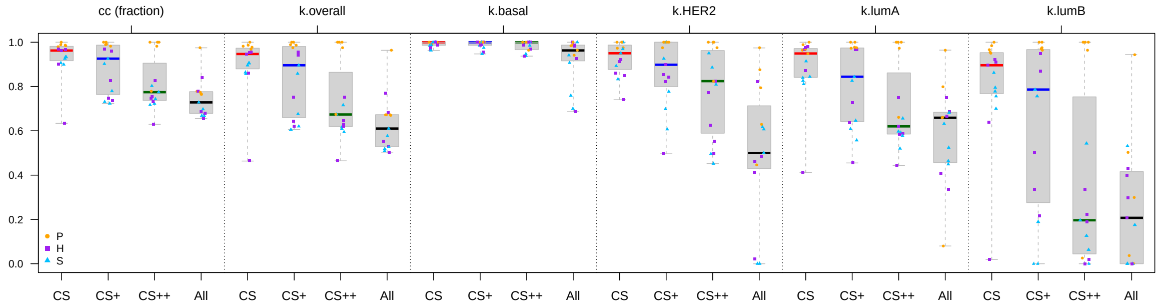

Figure S3. CS subtype identification by hierarchical clustering. For each of the training sets used to construct the five consensus sets (Table 2, main text) and for each of the IGLs S, H and P, four hierarchical clusterings were performed, labeled CS, CS+, CS++ and All (indicated on the x-axis for each panel). These respectively represent clusterings on the CS samples and three supersets of the consensus set. CS+: all samples for which PAM50 and all three SCMs are concordant, i.e. samples for which the St. Gallen criteria were left out of the CS inclusion criteria; CS++: all samples for which all three SCMs were concordant, i.e. samples for which the St. Gallen and the PAM50 CS inclusion criteria were not taken into account; All: the complete training set, i.e. when all CS inclusion criteria were dropped. Depicted are concordance percentage (cc) and kappa statistics between subtype assignments based on hierarchical clustering and the CS subtype labels. For a given set of samples concordance measures were always calculated on the CS samples only. The *intrinsic.cluster.predict* function from the *genefu* package was used to build a dendrogram (correlation distance, average linkage) and cut the dendrogram so as to obtain four clusters with a minimum of five samples per cluster [14]. Concordance between the cluster labels and the consensus set subtype labels was determined by mapping clusters to a subtype label using the *matchClasses* function (method="exact") from the R package *e1071*. This function computes all possible permutations between rows and columns of the confusion matrix between two vectors of labels and selects the mapping such that as many cases as possible are in a matched pair. See Table S3 for a detailed numerical summary.

over the CS samples only. When we only cluster CS samples, in all but one case almost perfect levels of concordance were obtained (Figure S3). However, it becomes increasingly more difficult to identify the CS subtype labels by HC when the training set becomes larger (and more heterogeneous). Furthermore, similar to Pusztai et al. [21], results strongly depended on the selected IGL. For the IGL P in nearly all cases almost perfect levels of concordance were obtained, however, not when clustering the CS samples in the presence of all additional samples. Concordance for the IGLs H and S was notably lower, especially when clustering CS samples in the presence of additional samples. Lowest concordance was observed for the luminal B subtype, whose concordance with CS subtype labels decreased strongly in the presence of additional samples.

3.2 Bimodality status of individual modules

Module scores are a core ingredient of both SCMs and STGs (Section 2). For a module score that is unimodally distributed, it is difficult to estimate a sensible cutoff for determining the over/underexpression status of the module for individual cases. The bimodality status of a module score, therefore, provides a good indication of the performance of SCM and STG subtyping schemes. We used the bimodality index (BMI) [17] to assess bimodality of the distribution of the module scores related to ER, HER2, and PGR signaling and proliferation on the five consensus sets (Table S4). In most instances all modules showed strong indications of bimodality (BMI$\geq$1.5). However, the level of bimodality depended on both the dataset and module composition. Furthermore, in some cases modules were only weakly bimodal (BMI$\geq$1.1) or

Subset	cc (all, %)	κ (all)	κ (basal)	κ (HER2)	κ (lumA)	κ (lumB)
CS	96.23	0.946	1.000	0.950	0.949	0.896
CS+	92.59	0.896	1.000	0.898	0.844	0.786
CS++	77.45	0.674	1.000	0.824	0.620	0.196
All	72.84	0.610	0.963	0.500	0.659	0.207

Table S3. CS subtype identification by hierarchical clustering. Numerical details of Figure S3: median percentage of concordant samples (cc) and median kappa statistics.

even not bimodal at all (BMI<1.1), in particular for the HER2-related module of Desmedt. Even though the module scores are not always strongly bimodal, the results provide solid ground for fitting the mixture models and cutoff values associated with SCM- and STG-based predictors.

3.3 Concordance of CS-based predictors on consensus sets

An important distinction between our approach and previous subtyping efforts is that our CS-based predictors were specifically designed to be highly concordant at the individual sample level. We first investigated the resubstitution performance, i.e. the ability of a CS-based predictor to correctly predict the subtype labels of the CS samples on which it was constructed. As expected, the resubstitution performance showed almost perfect levels of overall and subtype-specific concordance (Table S5).

A prerequisite for concordance over large validation cohorts is that predictors view each others training data in a consistent way. We, therefore, also considered the 'internal CS' validation performance, i.e. the ability of a CS-based predictor to predict the labels of all 812 CS samples, minus its own consensus training samples. Also in terms of internal CS validation performance, the CS-based predictors showed almost perfect levels of overall and subtype-specific concordance. The SCM.cs predictors showed the strongest levels of concordance (median κ=0.966, median cc=97.54%, Table S6), closely followed by the SSP.cs predictors (median κ=0.940, median cc=95.66%), with equally strong subtype-specific levels of concordance. These results demonstrate that CS-based predictors are highly concordant on the individual sample level on training data.

	ER HK	ER D	ER W	HER2 HK	HER2 D	HER2 W	PGR	Proliferation	AURKA HK	AURKA D	AURKA W
Bos	2.45	2.26	2.09	1.76	1.28	2.26	1.97	1.40	1.08	1.24	1.36
expO	3.11	1.94	1.94	1.40	1.14	1.72	1.71	1.78	1.65	1.57	1.52
Guedj	2.87	1.91	1.90	1.24	0.86	1.67	1.95	1.79	1.71	1.64	1.61
Li	3.63	2.39	2.22	1.16	1.09	1.52	1.93	1.86	1.61	1.68	1.64
Sabatier	2.90	2.55	2.62	1.44	0.94	1.53	1.98	1.87	1.52	1.70	1.63
BMI (median)	2.90	2.26	2.09	1.40	1.09	1.67	1.95	1.79	1.61	1.64	1.61
Nr. BMI $\geq$ 1.1	5	5	5	5	2	5	5	5	4	5	5
Nr. BMI $\geq$ 1.5	5	5	5	1	0	5	5	4	4	4	4

Table S4. Bimodality indices (BMI) of individual modules on consensus sets. Wang et al. [17] characterize a distribution as being bimodal if BMI $\geq$ 1.1 and strongly bimodal if BMI $\geq$ 1.5. The first row indicates the various modules used to measure ER, HER2, PGR and proliferation (Section 2). Proliferation was measured by the AURKA proliferation modules by Haibe-Kains et al. [14] (HK), Desmedt et al. [13] (D) and Wirapati et al. [15] (W) and the proliferation module (Proliferation) described in Table S2. BMI values are listed for each consensus set. The last three rows provide the median BMI value over all five consensus sets, the number of times the module was bimodal and the number of times the module was strongly bimodal, respectively.

Subset	cc (all, %)	κ (all)	κ (basal)	κ (HER2)	κ (lumA)	κ (lumB)
All	98.80	0.983	1.000	1.000	0.991	0.983
SCM.cs	99.57	0.994	1.000	1.000	1.000	1.000
SSP.cs	97.65	0.967	0.945	0.987	0.983	0.954
SCM.cs HK	99.57	0.994	1.000	1.000	1.000	0.991
SCM.cs D	99.06	0.987	1.000	1.000	1.000	0.982
SCM.cs W	100.0	1.000	1.000	1.000	1.000	1.000
SSP.cs S	95.68	0.939	0.945	0.987	0.920	0.904
SSP.cs H	97.65	0.967	0.927	0.987	0.991	0.954
SSP.cs P	98.59	0.980	0.962	0.983	0.991	0.985

Table S5. Resubstitution performance of CS-based predictors. Median percentage of concordant samples (cc) and median kappa statistics for CS-based predictors used to predict the subtype labels of their own consensus training set, i.e. to predict the associated CS labels. *Subset*: indicates the set of CS-based predictors over which the results were computed. Note that we report median values, it may therefore happen that for each individual subtype the median kappa statistic is equal to 1 but the overall median is not (2nd row).

Subset	cc (all, %)	κ (all)	κ (basal)	κ (HER2)	κ (lumA)	κ (lumB)
All	96.91	0.957	0.948	0.990	0.953	0.938
SCM.cs	97.54	0.966	0.991	0.996	0.951	0.948
SSP.cs	95.66	0.940	0.931	0.983	0.956	0.902
SCM.cs HK	97.55	0.966	1.000	0.997	0.949	0.941
SCM.cs D	96.99	0.958	0.945	0.996	0.943	0.937
SCM.cs W	98.44	0.978	0.991	0.996	0.967	0.959
SSP.cs S	94.63	0.926	0.933	0.988	0.887	0.870
SSP.cs H	96.77	0.955	0.882	0.984	0.971	0.932
SSP.cs P	97.55	0.966	0.955	0.972	0.970	0.960

Table S6. Internal CS validation performance of CS-based predictors. Median percentage of concordant samples (cc) and median kappa statistics for CS-based predictors used to predict the subtype labels of the union of all 812 CS samples, minus its own consensus training samples. *Subset*: indicates the set of CS-based predictors over which the results were computed.

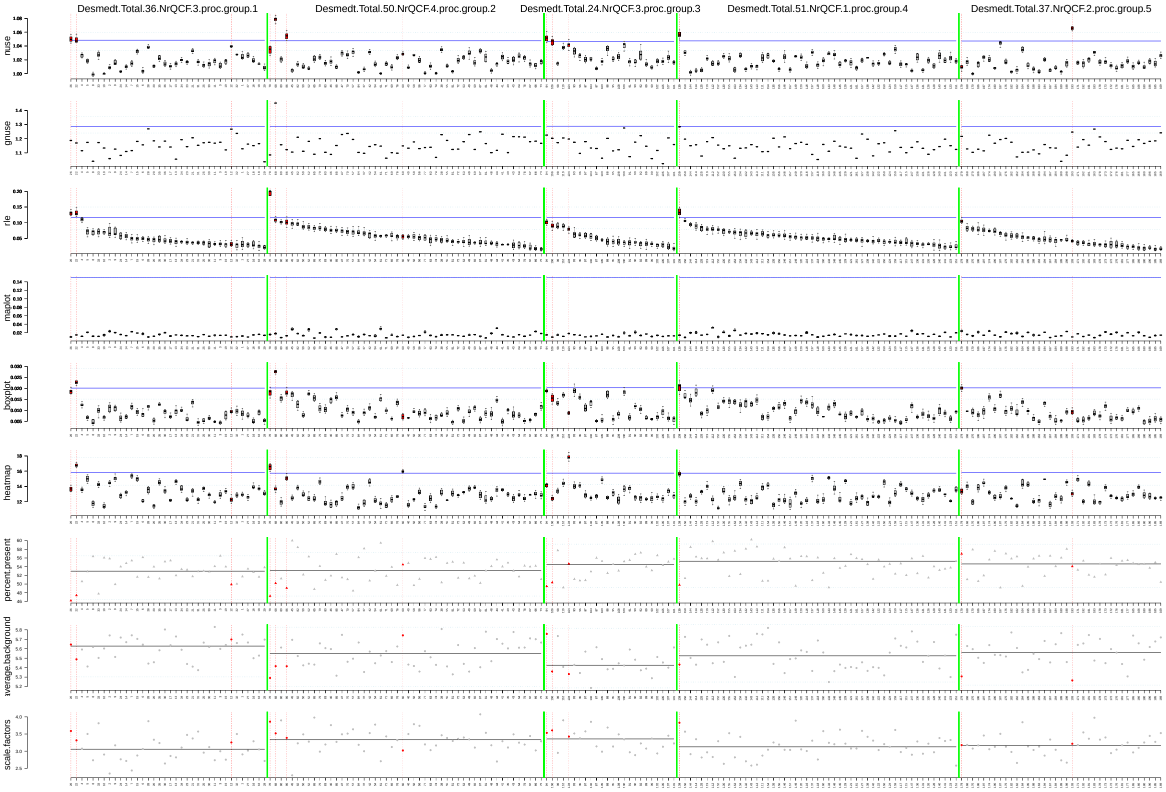
Desmedt.Total.36.NrQCF.3.proc.group.1
Desmedt.Total.50.NrQCF.4.proc.group.2
Desmedt.Total.24.NrQCF.3.proc.group.3
Desmedt.Total.51.NrQCF.1.proc.group.4
Desmedt.Total.37.NrQCF.2.proc.group.5
nuse
gnuse
rle
maplot
boxplot
heatmap
percent.present
average.background
scale.factors

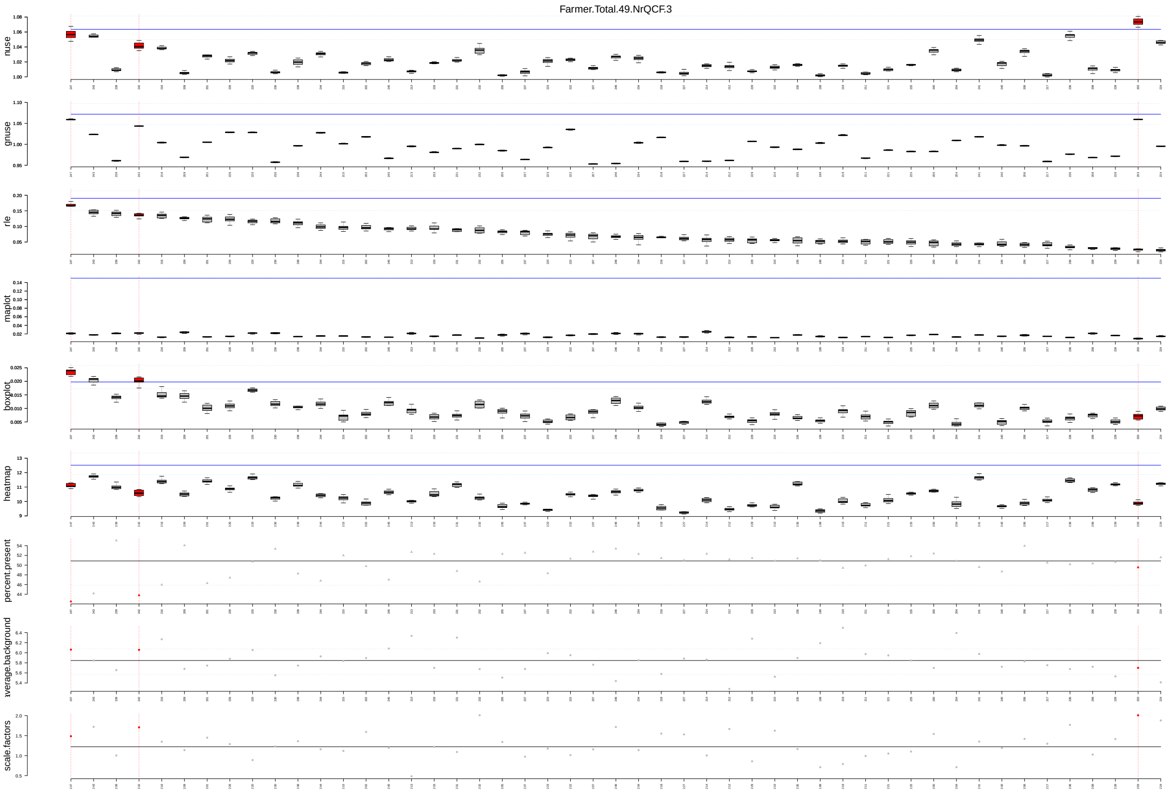
Farmer.Total.49.NrQCF.3
nuse
gnuse
rle
maplot
boxplot
heatmap
percent.present
average.background
scale.factors

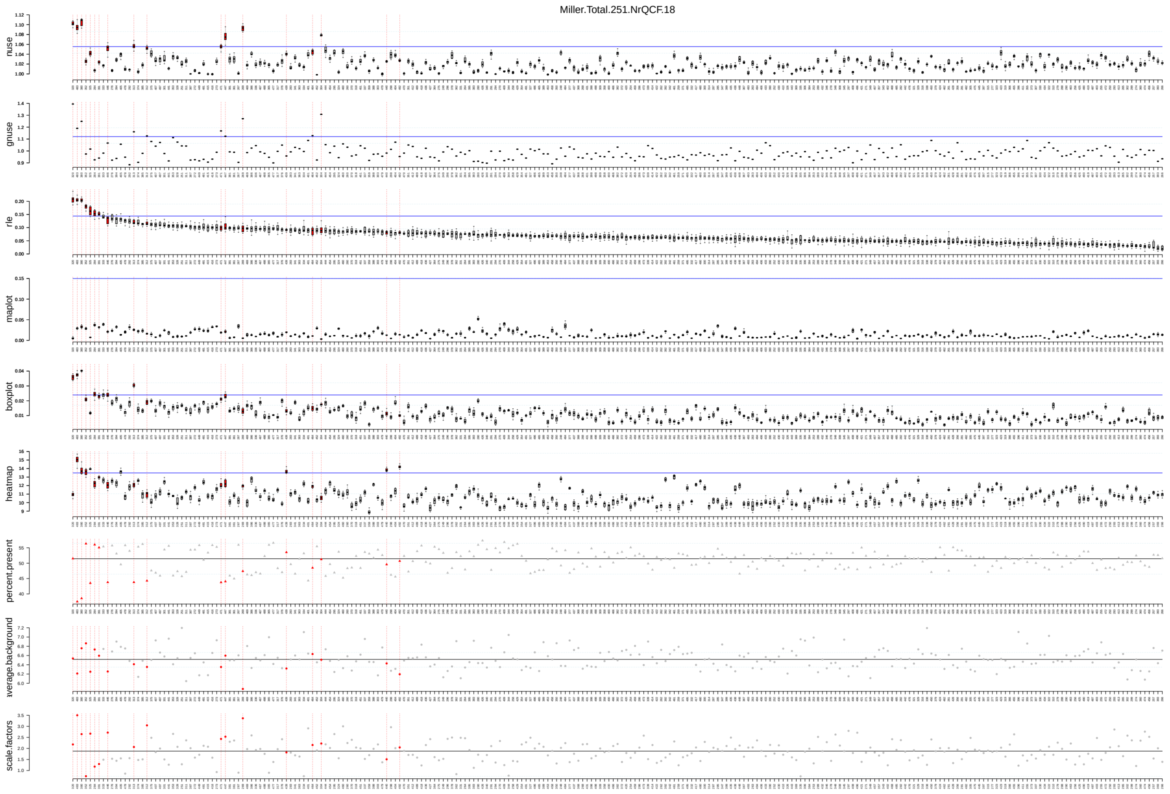
Miller.Total.251.NrQCF.18
nuse
gnuse
rle
maplot
boxplot
heatmap
percent.present
verage.background
scale.factors

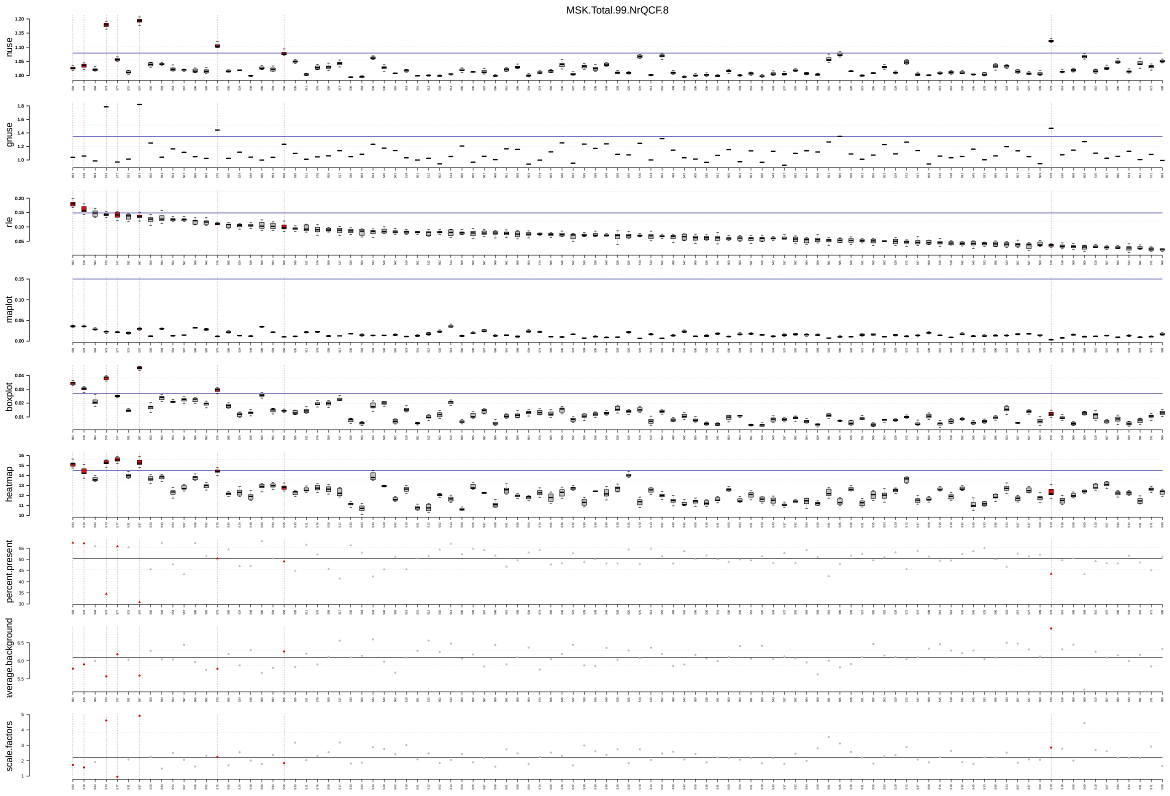
MSK.Total.99.NrQCF.8
nuse
gnuse
rle
maplot
boxplot
heatmap
percent.present
verage.background
scale.factors

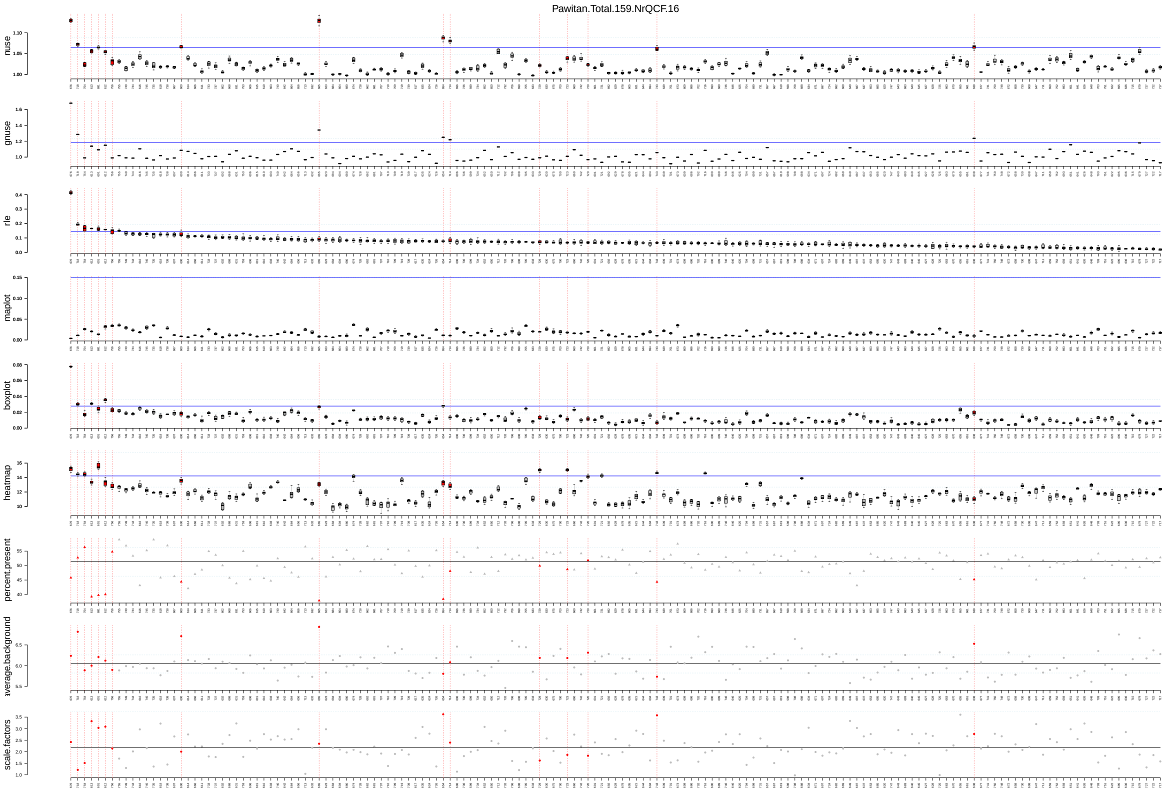
Pawitan.Total.159.NrQCF.16
nuse
gnuse
rle
maplot
boxplot
heatmap
percent.present
average.background
scale.factors

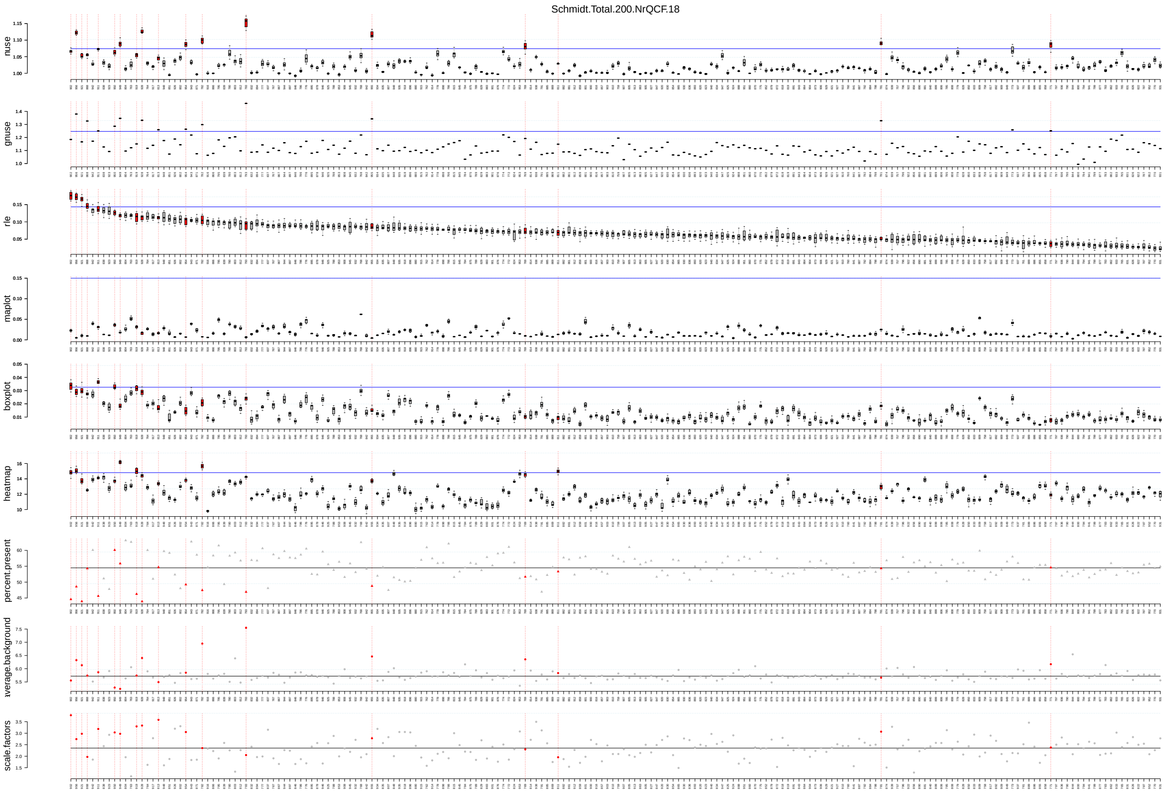
Schmidt.Total.200.NrQCF.18
nuse
gnuse
rle
maplot
boxplot
heatmap
percent.present
average.background
scale.factors

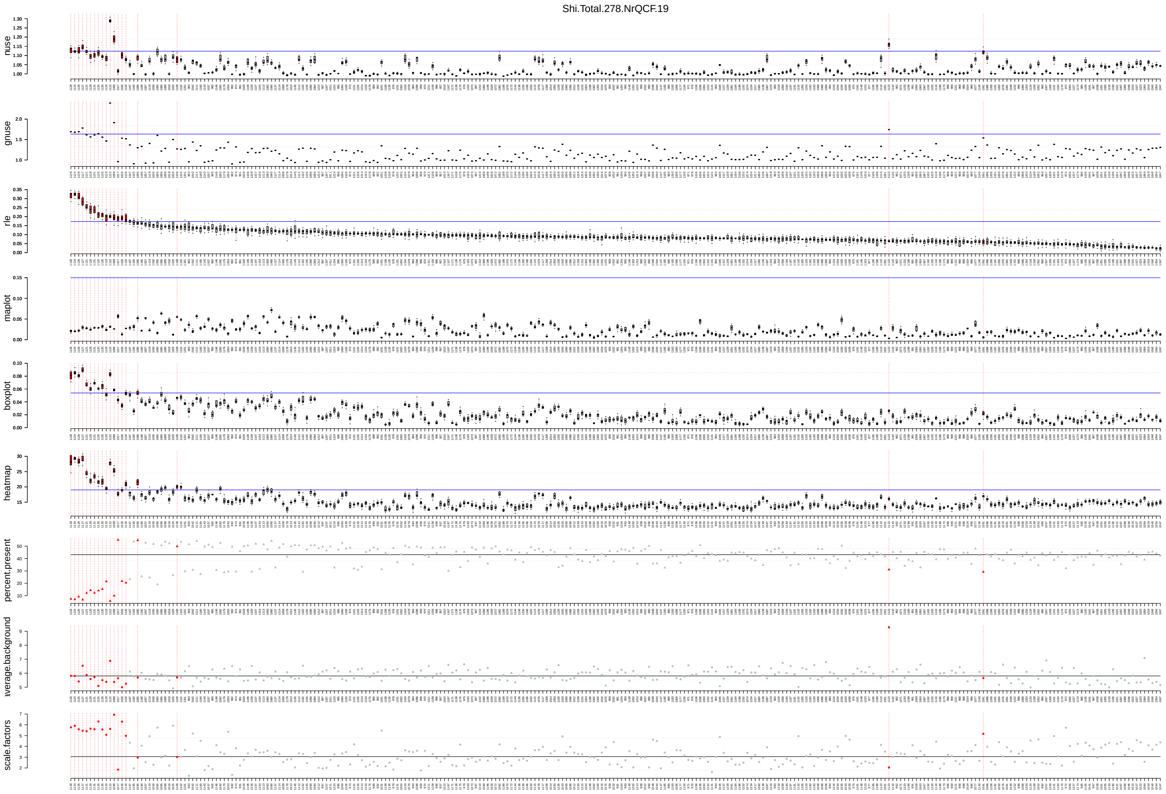
Shi.Total.278.NrQCF.19
nuse
gnuse
rle
maplot
boxplot
heatmap
percent.present
average.background
scale.factors

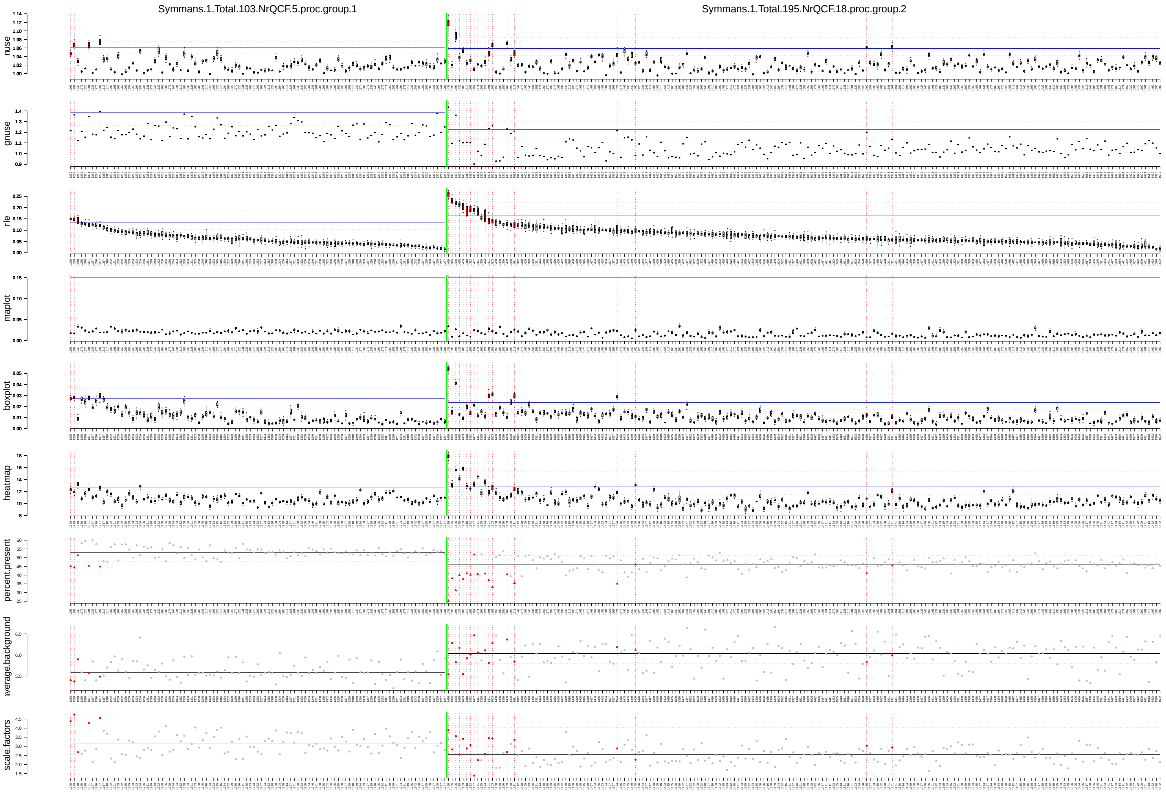
Symmans.1.Total.103.NrQCF.5.proc.group.1
Symmans.1.Total.195.NrQCF.18.proc.group.2
nuse
gnuse
rle
maplot
boxplot
heatmap
percent.present
average.background
scale.factors

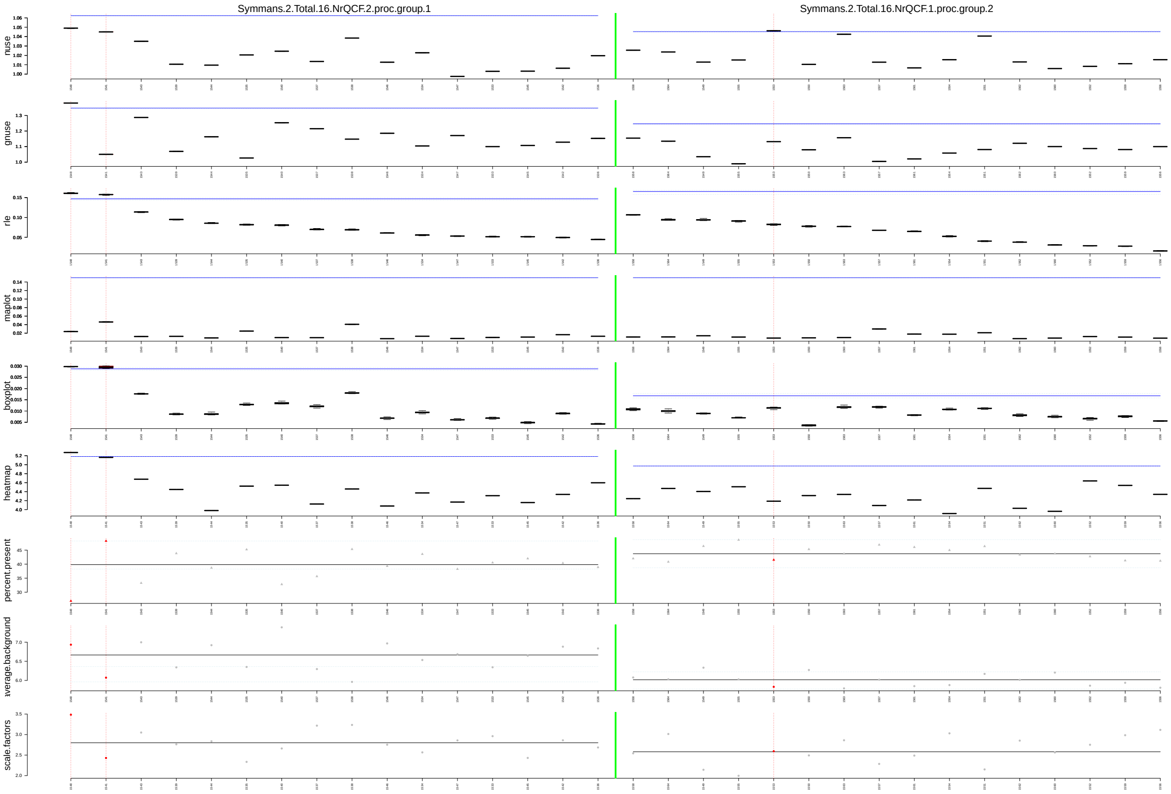
Symmans.2.Total.16.NrQCF.2.proc.group.1
Symmans.2.Total.16.NrQCF.1.proc.group.2
nuse
gnuse
rle
maplot
boxplot
heatmap
percent.present
average.background
scale.factors

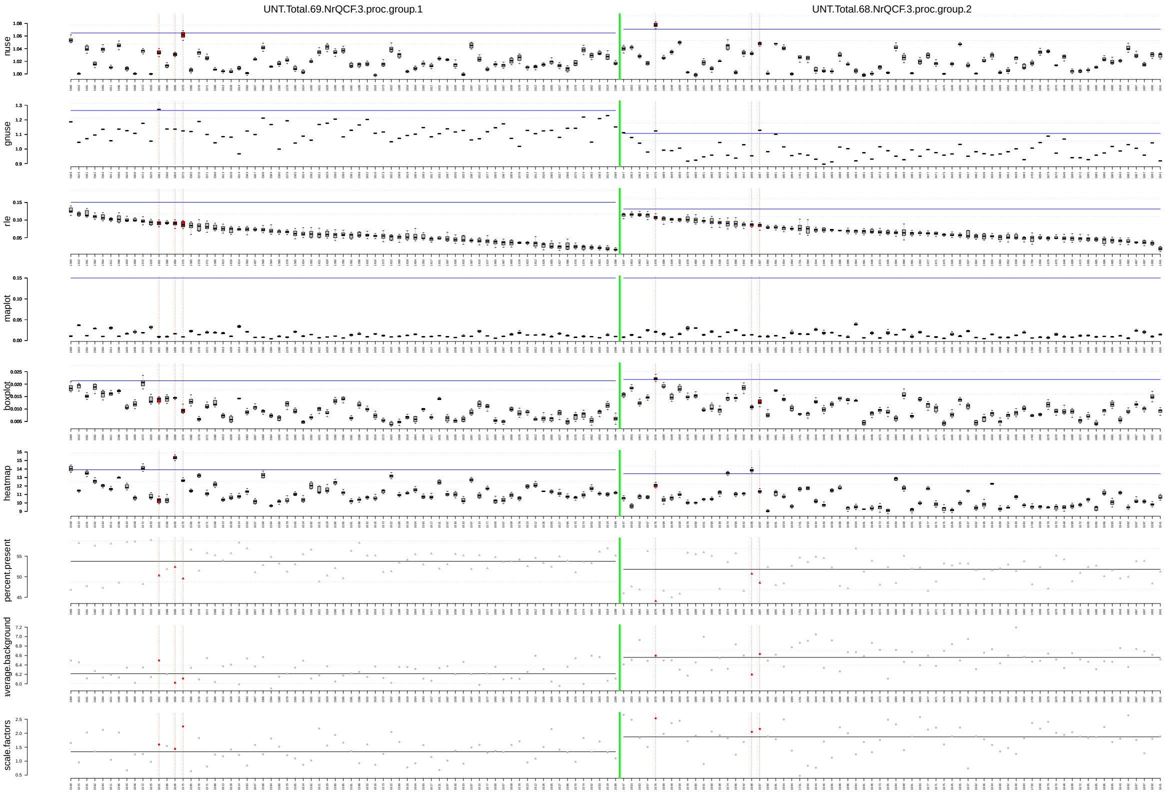
UNT.Total.69.NrQCF.3.proc.group.1
UNT.Total.68.NrQCF.3.proc.group.2
nuse
gnuse
rle
maplot
boxplot
heatmap
percent.present
average.background
scale.factors

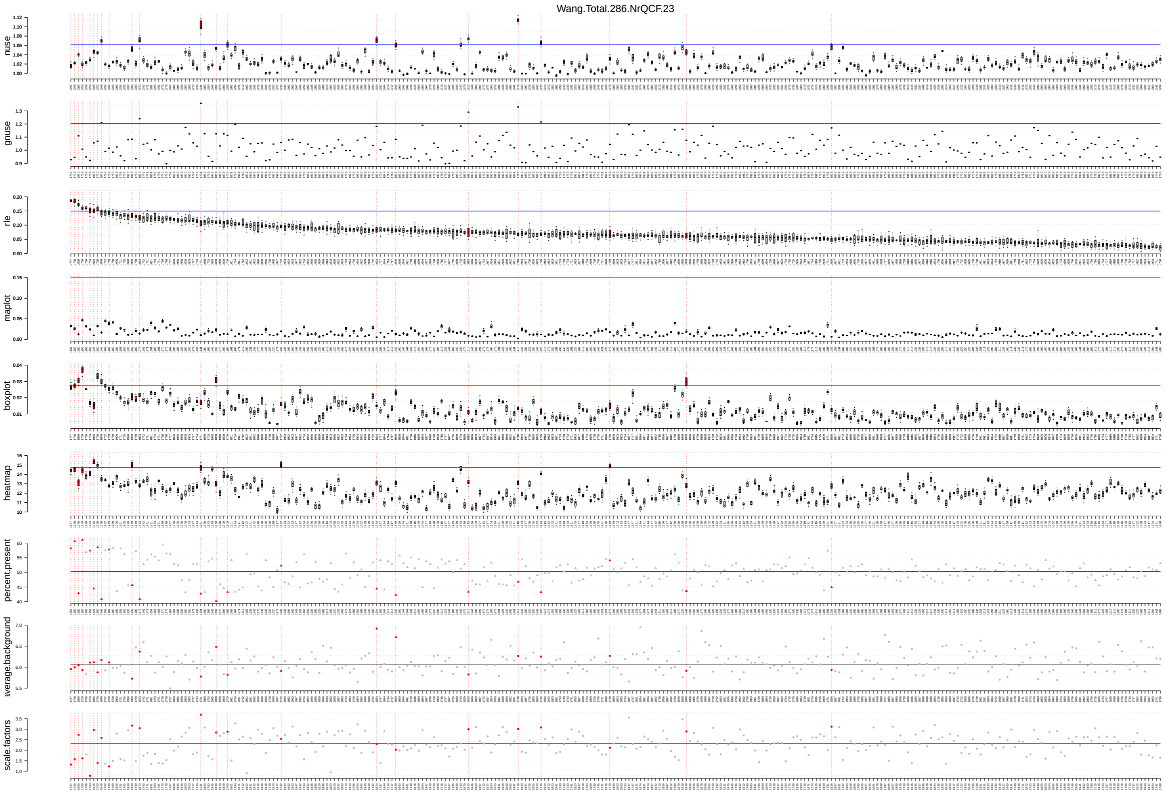
Wang.Total.286.NrQCF.23
nuse
gnuse
rle
maplot
boxplot
heatmap
percent.present
average.background
scale.factors

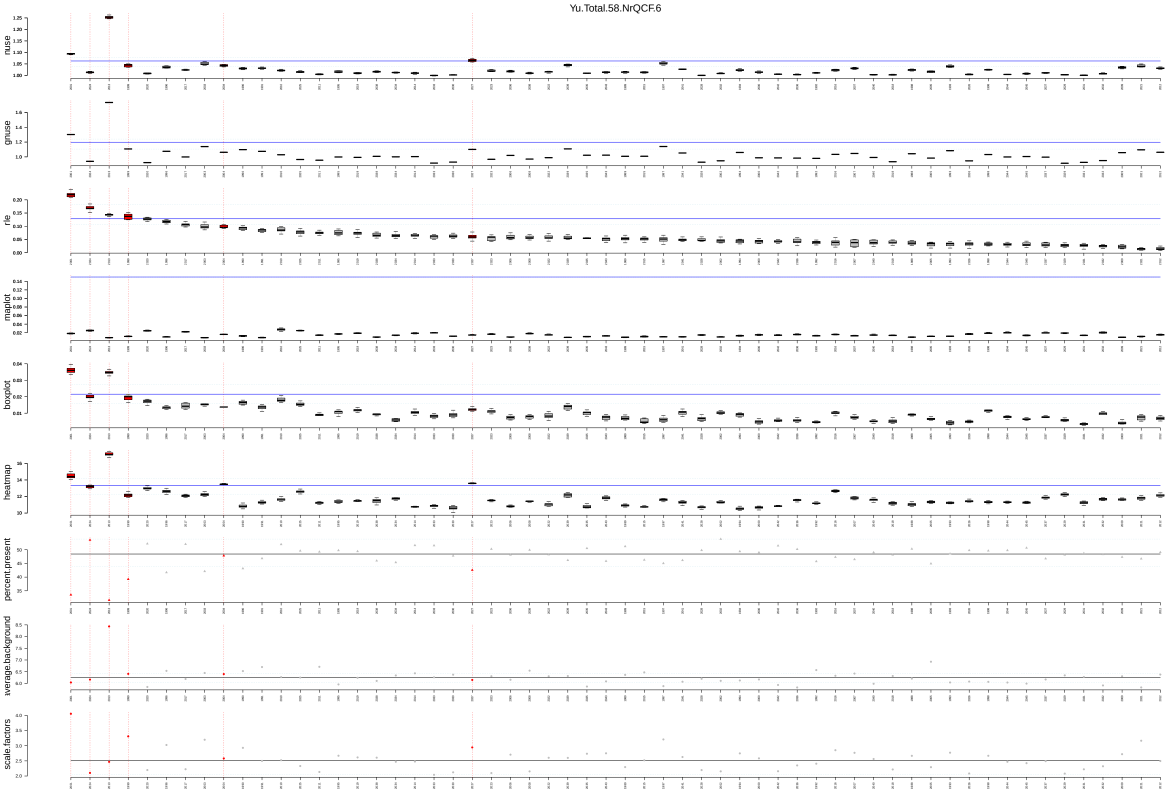
Yu.Total.58.NrQCF.6
nuse
gnuse
rle
maplot
boxplot
heatmap
percent.present
average.background
scale.factors

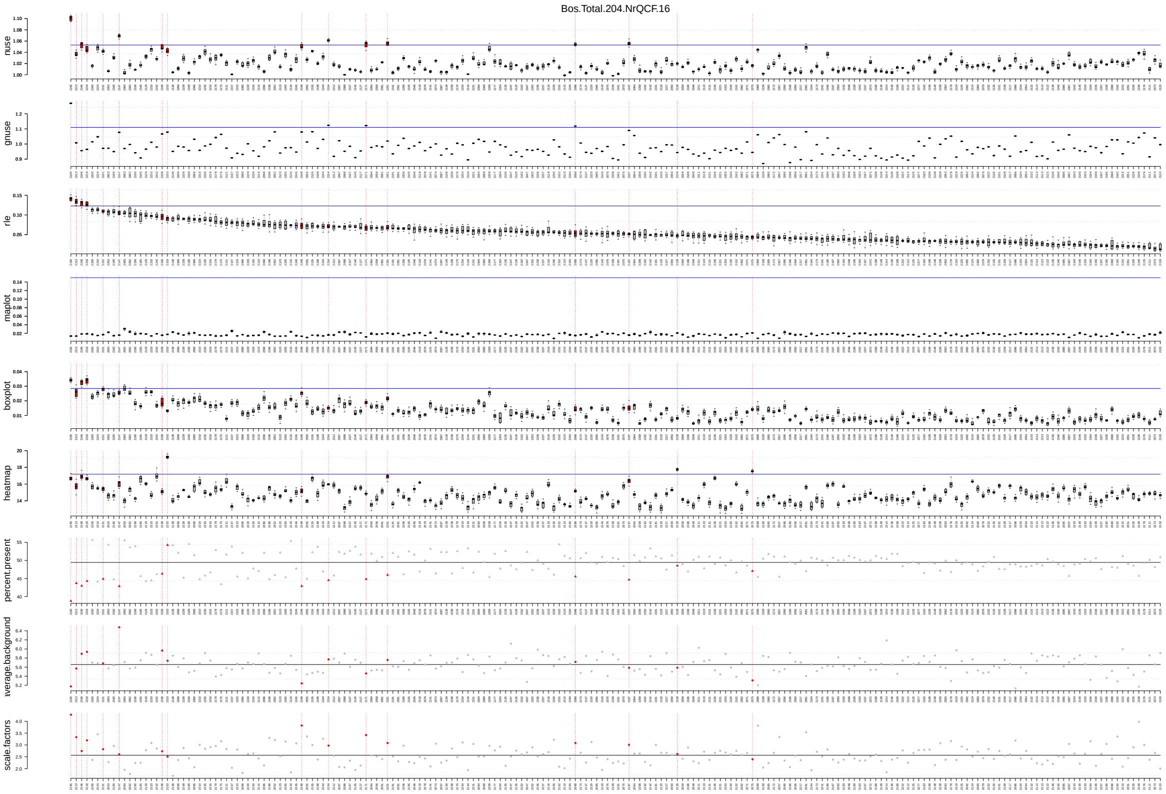
Bos.Total.204.NrQCF.16
nuse
gnuse
rle
maplot
boxplot
heatmap
percent.present
average.background
scale.factors

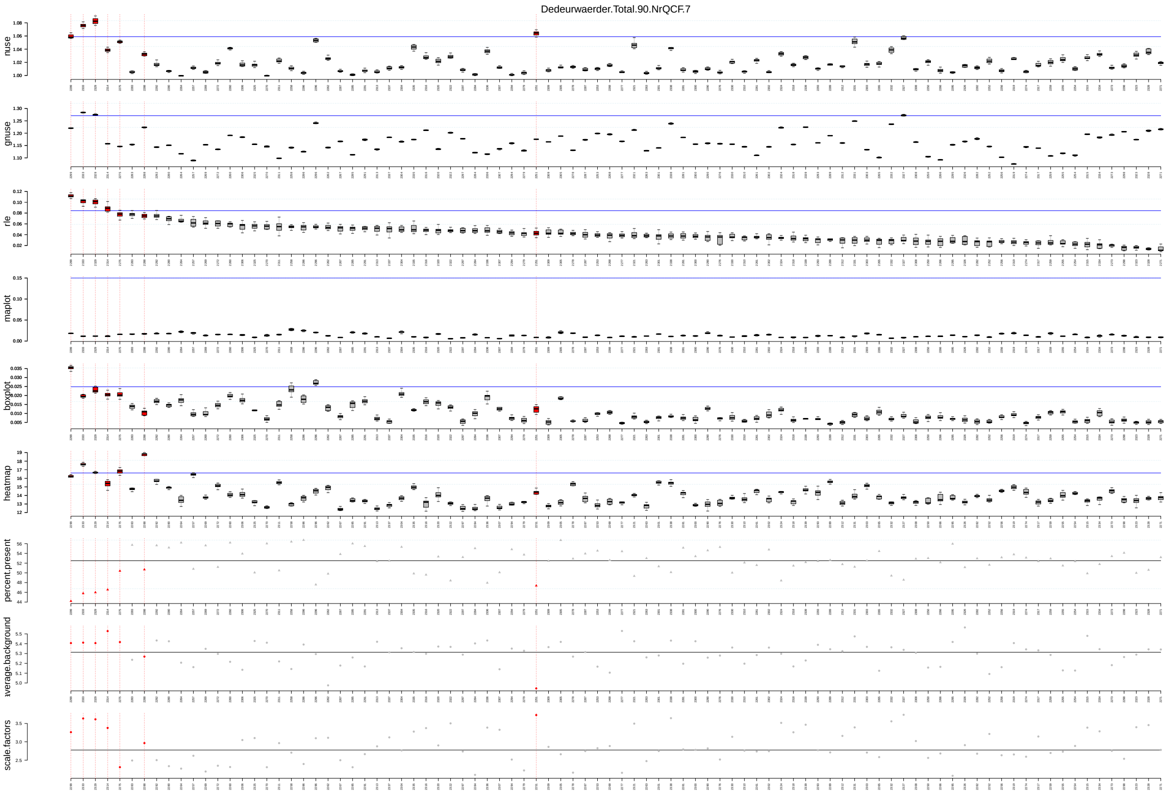
Dedeurwaerder.Total.90.NrQCF.7
nuse
gnuse
rle
maplot
boxplot
heatmap
percent.present
average.background
scale.factors

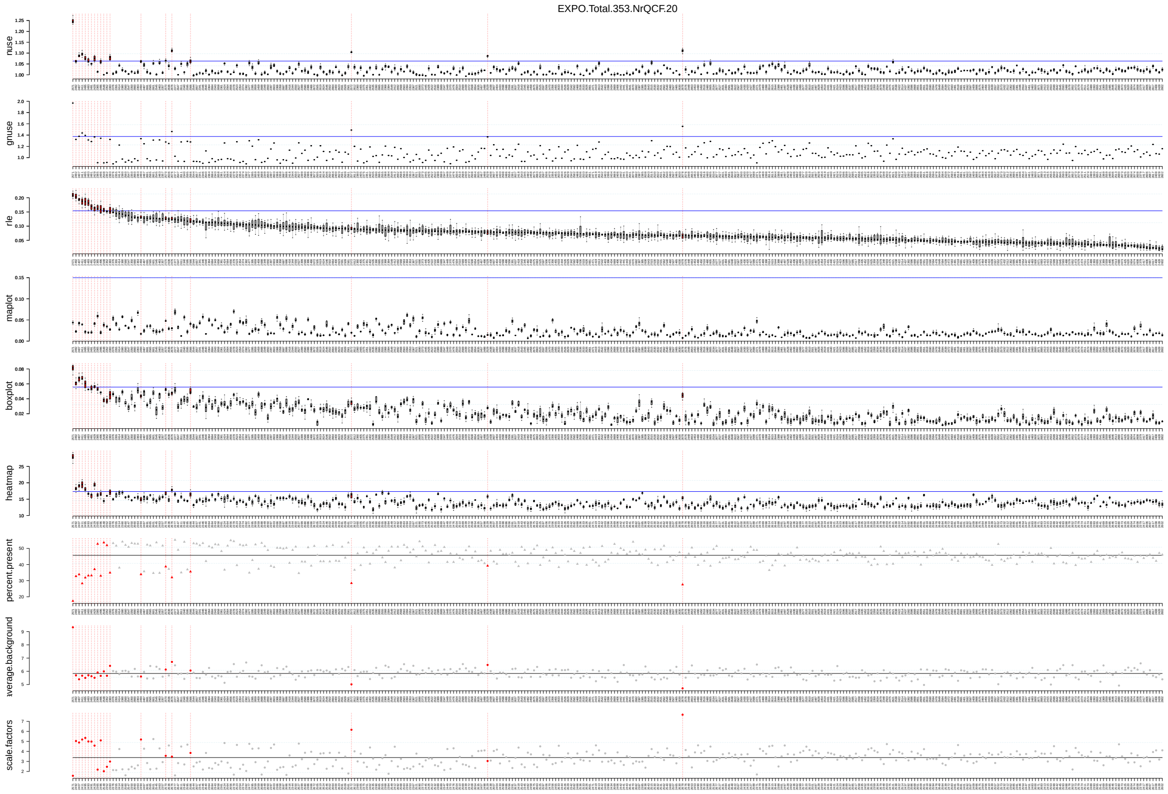
EXPO.Total.353.NrQCF.20
nuse
gnuse
rle
maplot
boxplot
heatmap
percent.present
average.background
scale.factors

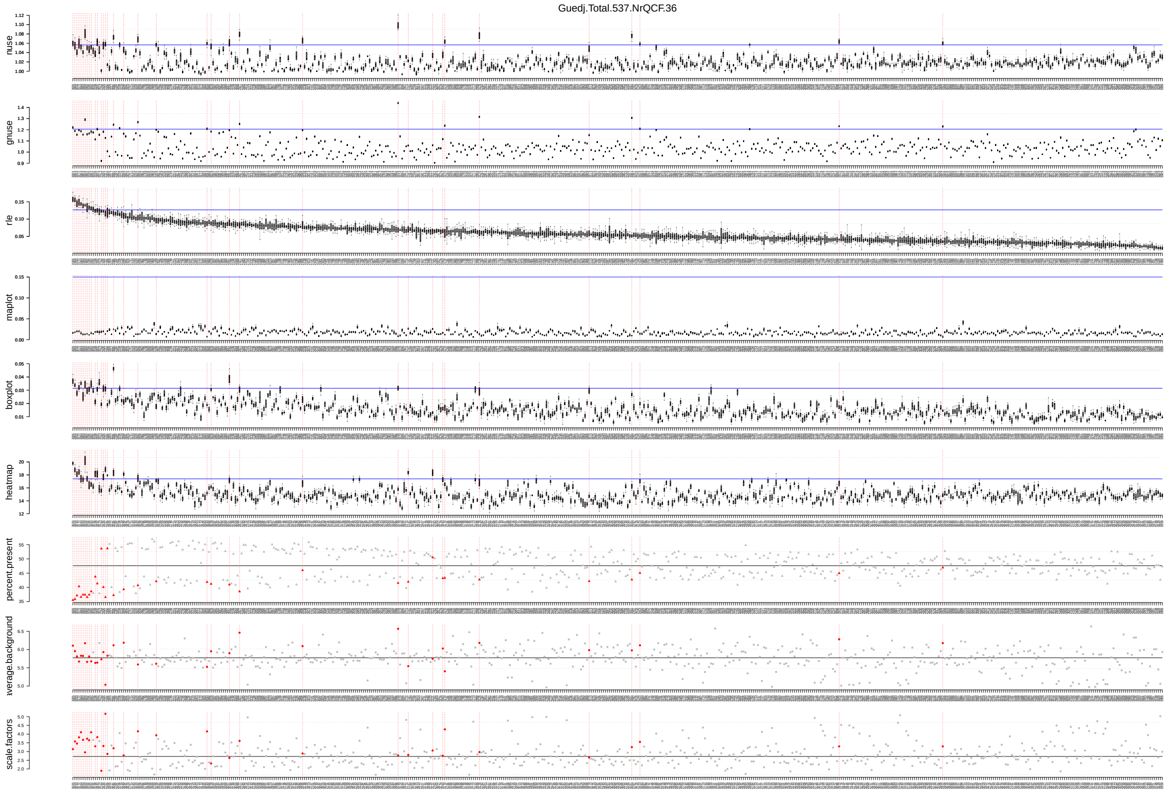
Guedj.Total.537.NrQCF.36
nuse
gnuse
rle
maplot
boxplot
heatmap
percent.present
average.background
scale.factors

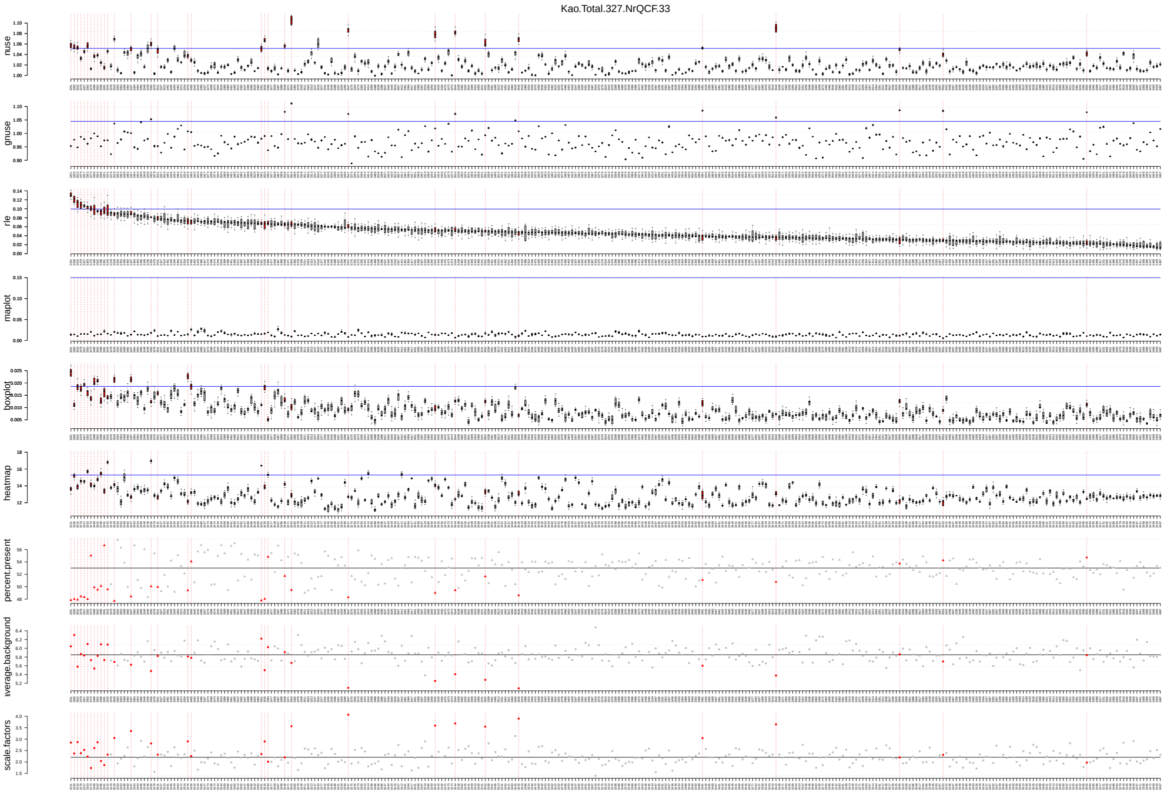
Kao.Total.327.NrQCF.33
nuse
gnuse
rle
maplot
boxplot
heatmap
percent.present
average.background
scale.factors

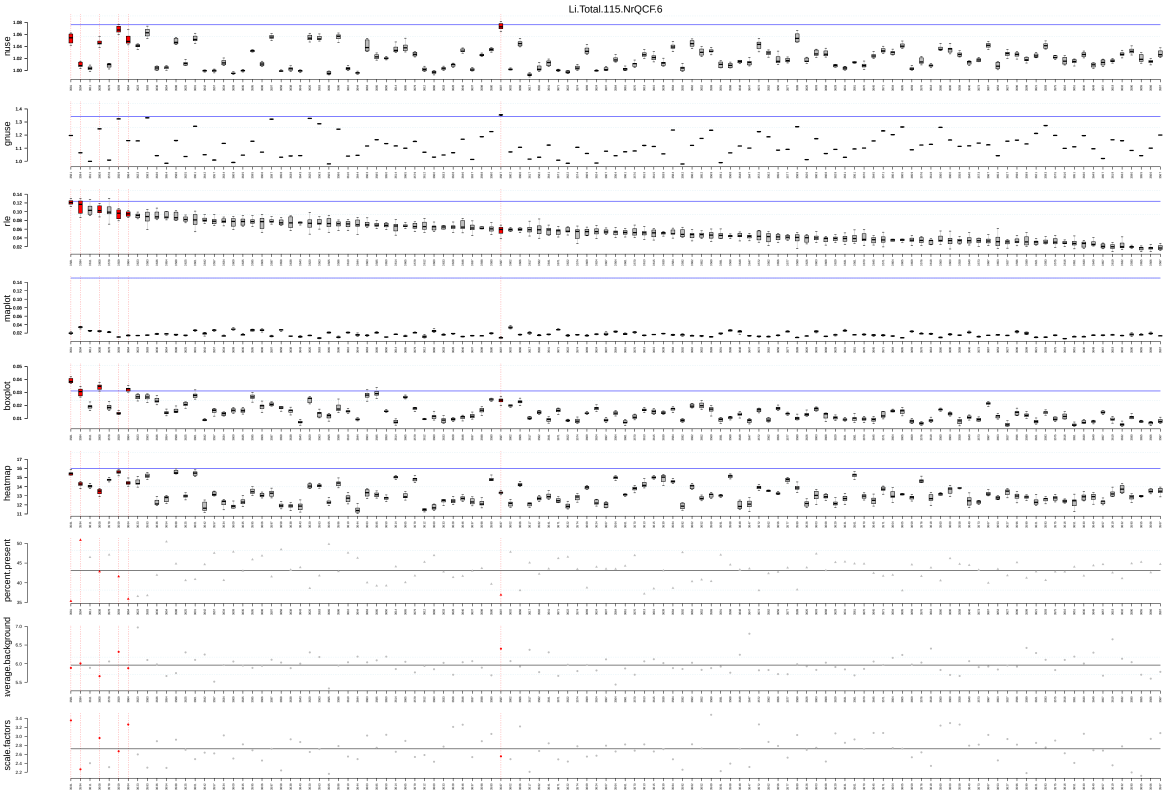
Li.Total.115.NrQCF.6
nuse
gnuse
rle
maplot
boxplot
heatmap
percent.present
average.background
scale.factors

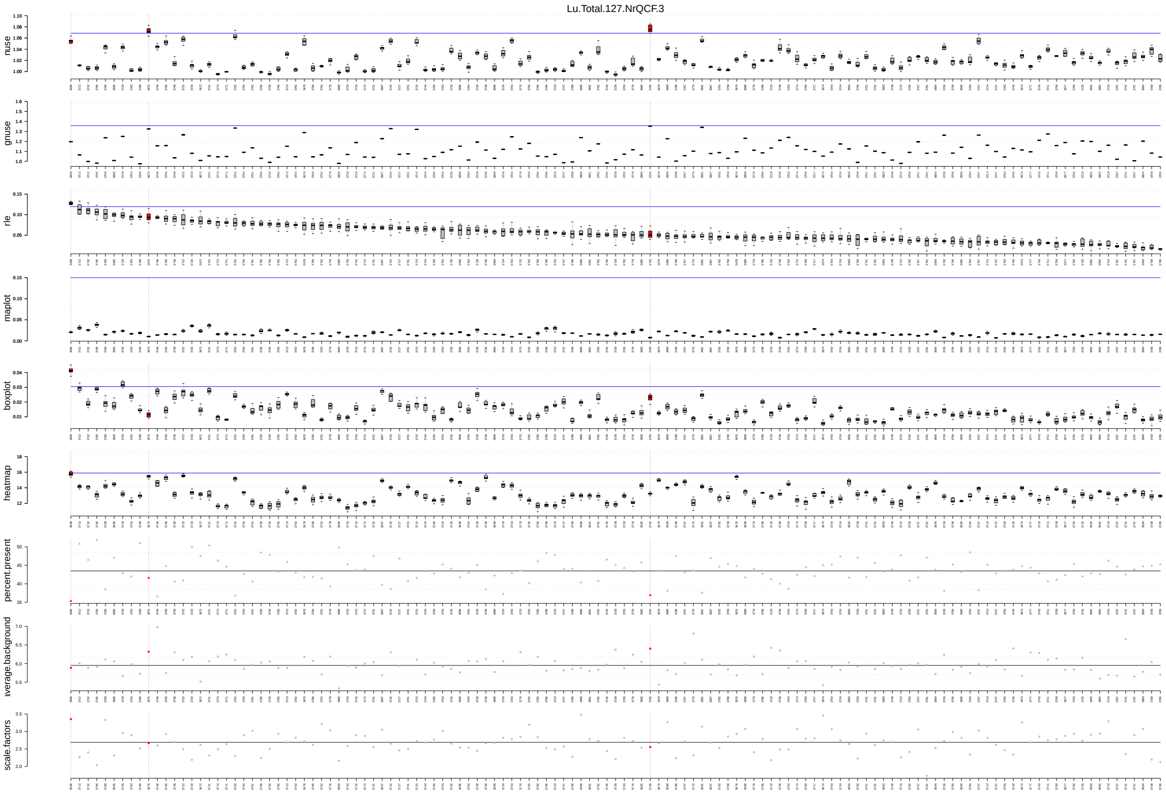
Lu.Total.127.NrQCF.3
nuse
gnuse
rle
maplot
boxplot
heatmap
percent.present
average.background
scale.factors

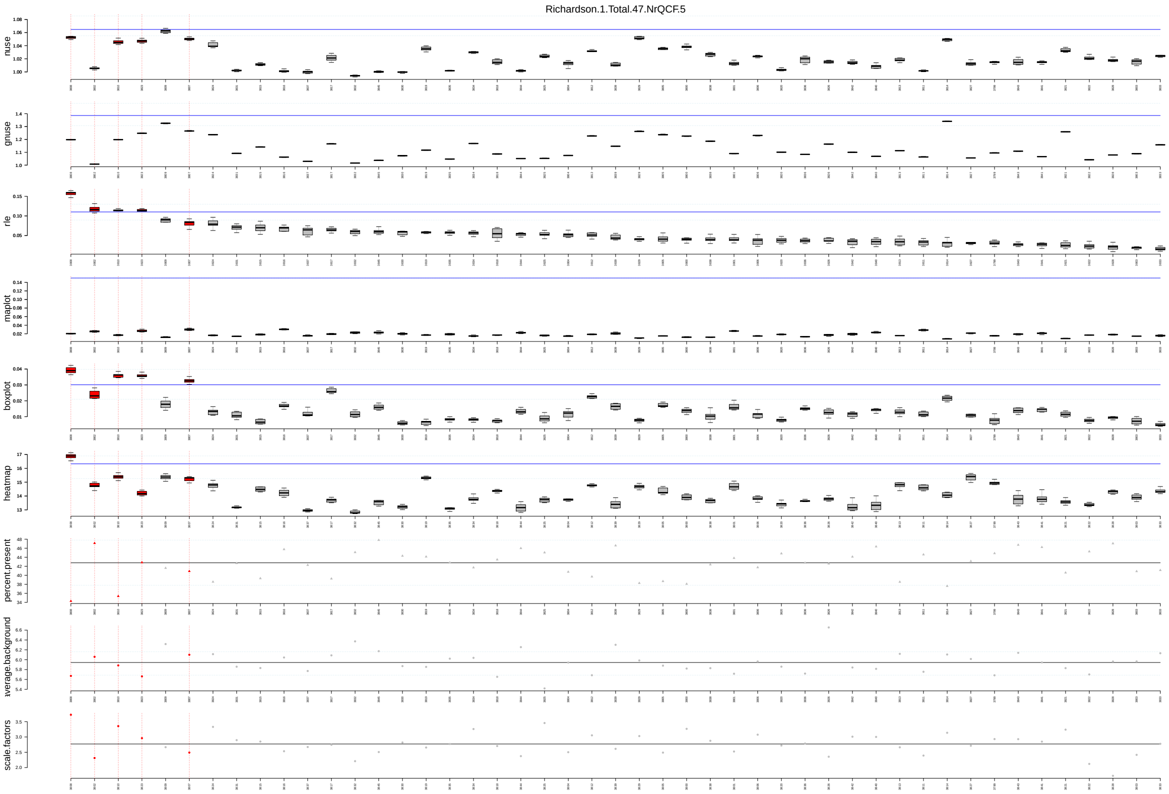
Richardson.1.Total.47.NrQCF.5
nuse
gnuse
rle
maplot
boxplot
heatmap
percent.present
average.background
scale.factors

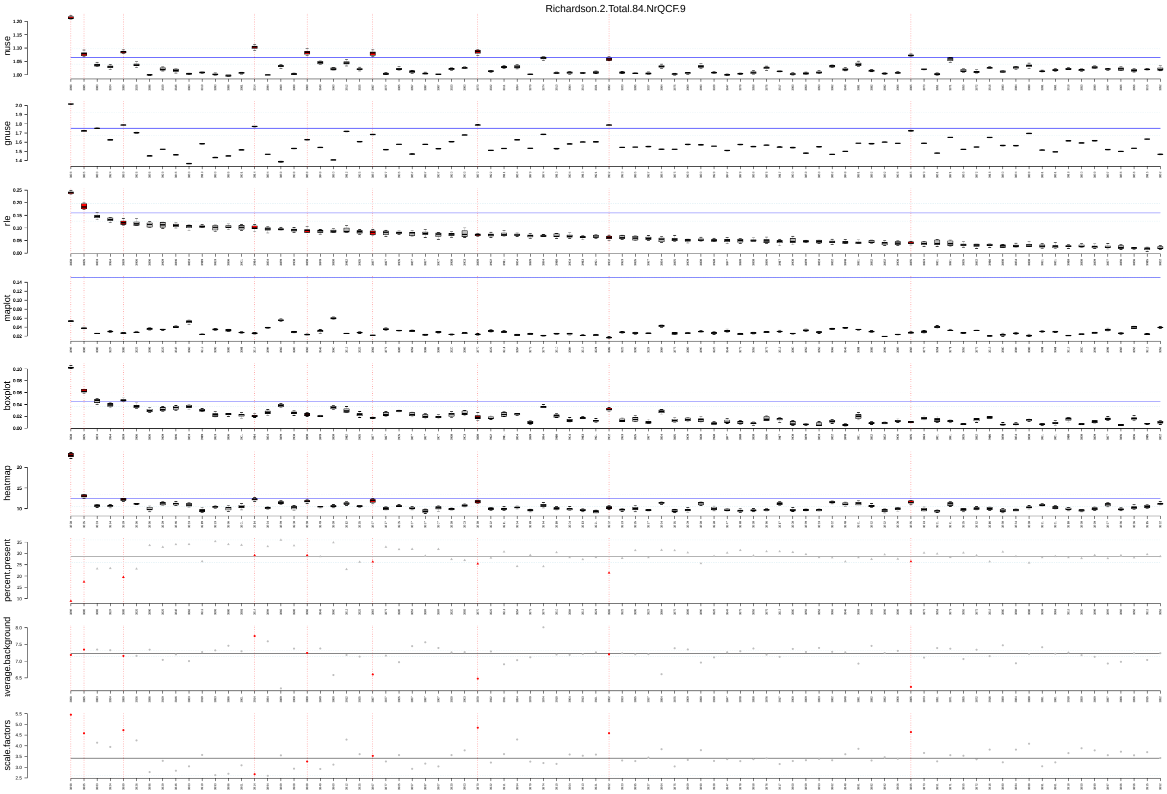
Richardson.2.Total.84.NrQCF.9
nuse
gnuse
rle
maplot
boxplot
heatmap
percent.present
average.background
scale.factors

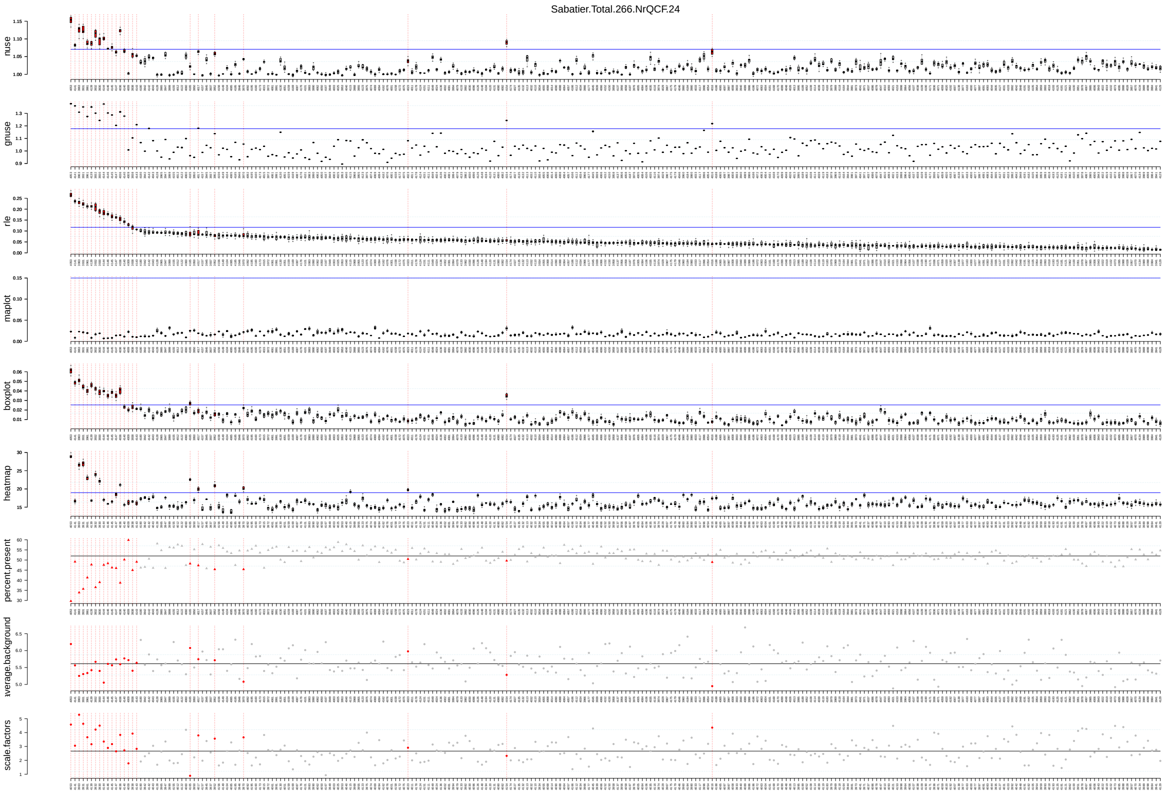
Sabatier.Total.266.NrQCF.24
nuse
gnuse
rle
maplot
boxplot
heatmap
percent.present
average.background
scale.factors

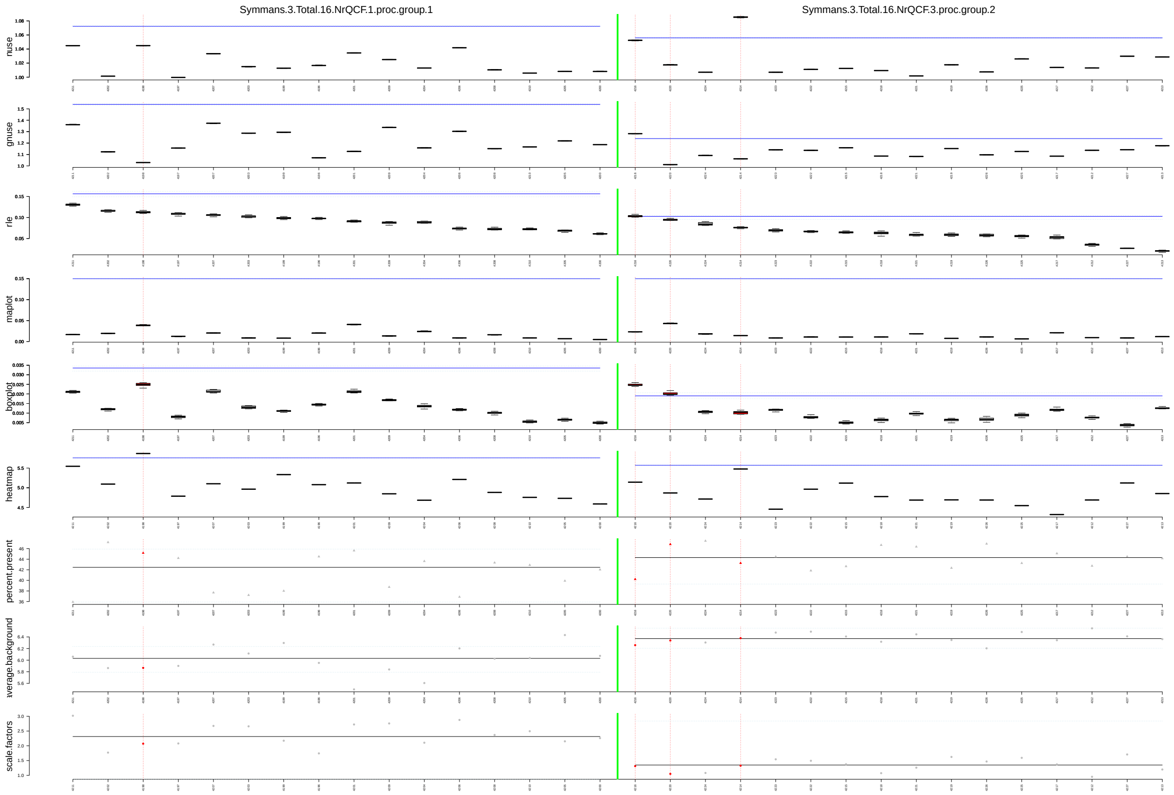
Symmans.3.Total.16.NrQCF.1.proc.group.1
Symmans.3.Total.16.NrQCF.3.proc.group.2
nuse
gnuse
rle
maplot
boxplot
heatmap
percent.present
scale.factors

Index	ID	CEL	NUSE	GNUSE	RLE	MA-plot	Boxplot	Heatmap
1	12	GSM177896.cel.gz	3	5	0	0	0	0
2	22	GSM177906.cel.gz	5	0	9	0	7	8
3	26	GSM177910.cel.gz	5	0	10	0	3	0
4	60	GSM177944.cel.gz	0	0	0	0	0	5
5	68	GSM177952.cel.gz	10	10	5	0	9	0
6	78	GSM177962.cel.gz	0	0	10	0	4	7
7	86	GSM177970.cel.gz	7	0	2	0	2	2
8	94	GSM177978.cel.gz	8	0	1	0	4	0
9	104	GSM177988.cel.gz	3	0	0	0	0	10
10	106	GSM177990.cel.gz	5	0	0	0	0	0
11	139	GSM178023.cel.gz	8	5	7	0	5	2
12	179	GSM178063.cel.gz	0	0	1	0	6	0
13	193	GSM178077.cel.gz	10	0	0	0	0	0
14	203	GSM26870.CEL.gz	10	1	0	0	0	0
15	242	GSM26909.CEL.gz	0	0	0	0	6	0
16	247	GSM26914.CEL.gz	3	4	2	0	8	0
17	269	GSM79172.CEL.gz	10	10	0	0	0	0
18	284	GSM79231.CEL.gz	0	0	6	0	3	0
19	304	GSM79314.CEL.gz	9	10	0	0	0	0
20	312	GSM79331.CEL.gz	1	6	1	0	1	0
21	313	GSM79337.CEL.gz	6	9	1	0	10	0
22	320	GSM79350.CEL.gz	10	10	10	0	10	0
23	325	GSM79355.CEL.gz	0	0	4	0	0	6
24	352	GSM79147.CEL.gz	0	0	10	0	3	4
25	380	GSM79194.CEL.gz	10	10	10	0	10	6
26	391	GSM79209.CEL.gz	0	0	7	0	1	1
27	439	GSM79270.CEL.gz	0	0	0	0	0	6
28	440	GSM79271.CEL.gz	0	0	0	0	0	9
29	446	GSM79278.CEL.gz	2	0	2	0	5	0
30	447	GSM79279.CEL.gz	9	4	2	0	4	0
31	464	GSM79303.CEL.gz	0	5	0	0	0	0
32	471	GSM79313.CEL.gz	3	8	0	0	3	0
33	483	GSM79334.CEL.gz	10	10	10	0	10	9
34	492	GSM79356.CEL.gz	0	0	0	0	0	7
35	556	GSM50091.CEL.gz	5	0	0	0	0	0
36	573	GSM50108.CEL.gz	10	10	3	0	9	7
37	575	GSM50110.CEL.gz	9	9	0	0	7	2
38	577	GSM50112.CEL.gz	0	0	4	0	3	10
39	578	GSM50113.CEL.gz	0	0	5	0	4	3
40	579	GSM50114.CEL.gz	10	10	0	0	0	0
41	583	GSM50118.CEL.gz	0	0	8	0	7	10
42	597	GSM50132.CEL.gz	10	10	3	0	10	7
43	600	GSM107074.CEL.gz	5	0	1	0	0	2
44	612	GSM107086.CEL.gz	2	4	6	0	10	0
45	613	GSM107087.CEL.gz	2	3	9	0	5	1
46	638	GSM107112.CEL.gz	6	10	0	0	0	0
47	654	GSM107129.CEL.gz	9	10	0	0	3	0
48	665	GSM107140.CEL.gz	10	10	0	0	5	1
49	676	GSM107151.CEL.gz	10	10	10	0	10	9
50	691	GSM107166.CEL.gz	4	0	7	0	2	10
51	714	GSM107189.CEL.gz	8	9	0	0	0	0
52	718	GSM107193.CEL.gz	5	10	10	0	7	3
53	720	GSM107195.CEL.gz	0	0	0	0	0	6
54	723	GSM107198.CEL.gz	0	0	0	0	0	9
55	729	GSM107204.CEL.gz	0	0	0	0	0	7
56	743	GSM107218.CEL.gz	3	0	0	0	0	6
57	754	GSM107229.CEL.gz	0	0	8	0	0	6
58	756	GSM107231.CEL.gz	0	0	6	0	3	0
59	769	GSM282385.CEL.gz	6	0	0	0	0	3
60	771	GSM282387.CEL.gz	6	5	0	0	0	0
61	781	GSM282397.CEL.gz	8	10	0	0	0	0
62	782	GSM282398.CEL.gz	8	5	0	0	1	6
63	793	GSM282409.CEL.gz	10	10	0	0	0	4
64	811	GSM282427.CEL.gz	0	0	0	0	0	5
65	813	GSM282429.CEL.gz	0	6	0	0	0	0
66	868	GSM282484.CEL.gz	0	9	3	0	1	0
67	902	GSM282518.CEL.gz	1	0	10	0	6	3
68	911	GSM282527.CEL.gz	5	3	4	0	7	2
69	919	GSM282535.CEL.gz	0	0	0	0	2	5
70	921	GSM282537.CEL.gz	0	0	9	0	3	0
71	928	GSM282544.CEL.gz	10	10	0	0	2	0
72	949	GSM282565.CEL.gz	9	10	0	0	0	9
73	950	GSM282566.CEL.gz	2	7	1	0	6	1
74	954	GSM282570.CEL.gz	8	7	0	0	0	1
75	955	GSM282571.CEL.gz	10	10	0	0	0	0
76	956	GSM282572.CEL.gz	10	10	9	0	5	7
77	1017	GSM505388_23678_AB01542166_24636.CEL.gz	0	0	7	0	1	2
78	1026	GSM505397_23678_AB01562100_26133.CEL.gz	1	1	5	0	0	4
79	1091	GSM505462_29539_AB01833522_35706.CEL.gz	6	1	0	0	0	0
80	1095	GSM505466_29539_AB01833699_35605.CEL.gz	0	0	2	0	3	7
81	1099	GSM505470_29539_AB01833733_35649.CEL.gz	10	10	6	0	4	9
82	1108	GSM505479_29539_AB01833780_35612.CEL.gz	2	0	0	0	3	5
83	1118	GSM505489_FL398-PERU53.CEL.gz	2	2	8	0	5	5
84	1120	GSM505491_FL454-713.CEL.gz	1	1	6	0	2	5
85	1121	GSM505492_U133A_FL112_US120_10_13_05.CEL.gz	5	5	10	0	8	9
86	1122	GSM505493_U133A_FL136_US123_11_14_05.CEL.gz	10	10	9	0	10	10
87	1123	GSM505494_U133A_FL137_US134_11_14_05.CEL.gz	7	8	0	0	0	1
88	1124	GSM505495_U133A_FL15_03_17_05.CEL.gz	5	7	10	0	10	10
89	1125	GSM505496_U133A_FL151_US129_12_08_05.CEL.gz	1	4	10	0	4	5
90	1126	GSM505497_U133A_FL161_US125_01_10_06.CEL.gz	4	8	10	0	8	10
91	1127	GSM505498_U133A_FL175_US147_01_13_06_2.CEL.gz	7	9	10	0	10	10
92	1128	GSM505499_U133A_FL32-US2_05_19_05.CEL.gz	6	7	10	0	8	10
93	1129	GSM505500_U133A_FL46-314_07_08_05.CEL.gz	1	2	8	0	5	9
94	1130	GSM505501_U133A_FL78_US92_09_01_05.CEL.gz	0	0	9	0	4	8
95	1131	GSM505502_U133A_FL80_US97_09_01_05.CEL.gz	2	1	8	0	6	7
96	1248	GSM441637.CEL.gz	0	0	4	0	0	7
97	1296	GSM441685.CEL.gz	0	0	8	0	3	5
98	1297	GSM441686.CEL.gz	10	6	3	0	4	5
99	1299	GSM441688.CEL.gz	10	3	6	0	5	0
100	1301	GSM441690.CEL.gz	8	1	3	0	6	1
101	1361	GSM441750.CEL.gz	7	0	0	0	0	1

Table S7. Overview of the 319 hybridizations rejected based on QC. Continued on next page.

Index	ID	CEL	NUSE	GNUSE	RLE	MA-plot	Boxplot	Heatmap
102	1371	GSM441760.CEL.gz	0	0	8	0	0	7
103	1382	GSM441771.CEL.gz	0	0	7	0	0	2
104	1395	GSM441784.CEL.gz	0	0	9	0	0	4
105	1400	GSM441789.CEL.gz	0	0	0	0	0	5
106	1403	GSM441792.CEL.gz	2	0	8	0	0	10
107	1418	GSM441807.CEL.gz	0	0	7	0	2	4
108	1424	GSM441813.CEL.gz	0	0	3	0	0	7
109	1425	GSM441814.CEL.gz	0	0	10	0	0	7
110	1438	GSM441827.CEL.gz	10	10	10	0	10	10
111	1457	GSM441846.CEL.gz	0	0	9	0	3	7
112	1460	GSM441849.CEL.gz	6	0	0	0	0	0
113	1469	GSM441858.CEL.gz	7	4	0	0	0	0
114	1491	GSM441880.CEL.gz	0	3	1	0	9	1
115	1496	GSM441885.CEL.gz	10	10	10	0	10	10
116	1503	GSM441892.CEL.gz	6	9	2	0	9	3
117	1511	GSM441900.CEL.gz	1	0	0	0	9	3
118	1524	GSM441913.CEL.gz	2	1	0	0	7	2
119	1541	GSM441356.CEL.gz	0	0	10	0	8	0
120	1548	GSM441363.CEL.gz	0	10	10	0	10	10
121	1553	GSM441336.CEL.gz	10	0	0	0	0	0
122	1575	gsm65878.cel.gz	6	0	0	0	0	0
123	1601	gsm65849.cel.gz	0	6	0	0	0	0
124	1606	gsm65852.cel.gz	0	0	0	0	0	9
125	1676	gsm65794.cel.gz	9	6	1	0	4	0
126	1687	gsm65805.cel.gz	0	7	0	0	0	0
127	1698	gsm65816.cel.gz	0	0	0	0	0	9
128	1709	GSM36835.CEL.gz	5	0	1	0	0	1
129	1718	GSM36861.CEL.gz	10	10	0	0	0	0
130	1725	GSM36875.CEL.gz	4	5	0	0	0	0
131	1731	GSM36900.CEL.gz	10	10	0	0	0	4
132	1732	GSM36901.CEL.gz	0	0	0	0	0	8
133	1750	GSM36966.CEL.gz	0	0	5	0	9	5
134	1753	GSM36969.CEL.gz	0	0	5	0	0	1
135	1757	GSM36991.CEL.gz	0	0	9	0	1	0
136	1758	GSM36992.CEL.gz	0	0	10	0	6	4
137	1759	GSM36993.CEL.gz	0	0	7	0	10	1
138	1769	GSM36879.CEL.gz	0	0	5	0	4	0
139	1779	GSM36905.CEL.gz	0	0	0	0	0	6
140	1813	GSM36997.CEL.gz	10	10	0	0	0	0
141	1824	GSM37030.CEL.gz	7	0	0	0	0	0
142	1836	GSM37052.CEL.gz	5	1	0	0	0	0
143	1848	GSM36778.CEL.gz	9	7	3	0	5	1
144	1849	GSM36787.CEL.gz	7	0	0	0	2	0
145	1858	GSM36813.CEL.gz	2	0	0	0	8	0
146	1873	GSM36811.CEL.gz	9	6	1	0	0	0
147	1925	GSM36984.CEL.gz	0	0	5	0	0	6
148	1949	GSM36933.CEL.gz	0	0	0	0	7	0
149	1959	GSM36795.CEL.gz	2	0	4	0	0	5
150	1980	GSM37044.CEL.gz	0	0	9	0	6	0
151	1999	GSM120659.CEL.gz	2	1	5	0	2	0
152	2001	GSM120661.CEL.gz	10	10	10	0	10	10
153	2004	GSM120665.CEL.gz	1	0	0	0	0	5
154	2013	GSM120670.CEL.gz	10	10	5	0	10	10
155	2024	GSM120683.CEL.gz	0	0	8	0	4	2
156	2027	GSM120686.CEL.gz	4	0	0	0	0	6
157	2075	GSM308285.CEL.gz	0	0	0	0	0	5
158	2098	GSM308308.CEL.gz	5	6	0	0	0	0
159	2108	GSM308319.CEL.gz	5	0	0	0	0	0
160	2128	GSM308339.CEL.gz	0	0	0	0	0	6
161	2147	GSM308358.CEL.gz	10	2	4	0	1	0
162	2151	GSM308362.CEL.gz	0	0	3	0	6	0
163	2153	GSM308364.CEL.gz	2	2	0	0	0	10
164	2154	GSM308365.CEL.gz	9	4	0	0	0	0
165	2171	GSM308382.CEL.gz	6	5	0	0	0	0
166	2195	GSM308406.CEL.gz	10	10	8	0	7	1
167	2197	GSM308408.CEL.gz	6	1	0	0	0	1
168	2201	GSM308412.CEL.gz	7	0	0	0	0	3
169	2213	GSM308424.CEL.gz	0	0	9	0	3	0
170	2218	GSM308429.CEL.gz	2	0	5	0	6	1
171	2246	GSM308457.CEL.gz	5	0	8	0	9	2
172	2248	GSM308459.CEL.gz	7	1	0	0	1	0
173	2251	GSM519723.CEL.gz	6	0	0	0	0	0
174	2275	GSM519747.CEL.gz	2	0	3	0	2	5
175	2288	GSM519760.CEL.gz	0	0	1	0	0	10
176	2299	GSM519772.CEL.gz	5	0	10	0	10	3
177	2314	GSM519787.CEL.gz	0	0	6	0	1	0
178	2329	GSM519802.CEL.gz	10	4	9	0	3	5
179	2333	GSM519806.CEL.gz	10	7	10	0	1	10
180	2352	GSM38062.CEL.gz	0	0	5	0	0	0
181	2365	GSM46891.CEL.gz	0	0	6	0	4	2
182	2383	GSM46908.CEL.gz	10	2	4	0	1	0
183	2407	GSM53034.CEL.gz	8	3	8	0	8	9
184	2411	GSM53109.CEL.gz	9	6	7	0	7	9
185	2429	GSM76613.CEL.gz	7	2	8	0	5	8
186	2491	GSM138035.CEL.gz	2	0	7	0	3	1
187	2492	GSM138028.CEL.gz	3	1	9	0	6	8
188	2493	GSM138031.CEL.gz	3	2	8	0	3	2
189	2494	GSM137950.CEL.gz	5	2	0	0	0	0
190	2495	GSM137943.CEL.gz	6	4	5	0	4	5
191	2496	GSM137944.CEL.gz	5	5	4	0	2	1
192	2529	GSM179932.CEL.gz	10	4	0	0	0	1
193	2548	GSM231887.CEL.gz	0	0	6	0	1	0
194	2566	GSM152569.CEL.gz	2	0	0	0	5	0
195	2570	GSM53161.CEL.gz	5	0	1	0	3	0
196	2571	GSM53147.CEL.gz	10	10	9	0	10	10
197	2572	GSM53131.CEL.gz	10	10	0	0	0	2
198	2676	GSM231918.CEL.gz	10	8	1	0	2	6
199	2678	GSM277707.CEL.gz	10	9	0	0	0	0
200	2747	FB_1214_U133_2.CEL	0	0	0	0	0	9
201	2757	RLi_74_U133_2.CEL	3	0	1	0	2	8
202	2764	FB_3562_U133_2.CEL	0	0	5	0	0	0
203	2802	HdT_1025_U133_2.CEL	5	0	4	0	4	3

Table S7. Overview of the 319 hybridizations rejected based on QC. Continued on next page.

Index	ID	CEL	NUSE	GNUSE	RLE	MA-plot	Boxplot	Heatmap
204	2803	HdT_10324_U133_2.CEL	0	1	7	0	3	2
205	2804	HdT_10381_U133_2.CEL	4	3	8	0	7	7
206	2820	DB_73_U133_2.CEL	9	10	8	0	7	10
207	2832	DB_9941_U133_2.CEL	7	5	6	0	6	8
208	2835	DB_9077_U133_2.CEL	5	0	3	0	1	3
209	2842	DB_9983_U133_2.CEL	7	9	1	0	0	1
210	2912	071213-18.CEL	7	5	0	0	0	0
211	2914	071213-20.CEL	8	5	0	0	0	4
212	2945	090806-07.CEL	6	5	0	0	2	3
213	2956	040706-22.CEL	0	0	3	0	0	6
214	2970	071213-04.CEL	6	7	0	0	0	0
215	2977	071213-01.CEL	9	6	0	0	3	0
216	3033	HdT_9913_U133_2.CEL	0	0	7	0	1	6
217	3043	DB_69_U133_2.CEL	5	5	0	0	0	0
218	3052	HdT_3411_U133_2.CEL	6	0	1	0	9	5
219	3055	HdT_9911_U133_2.CEL	7	6	9	0	9	10
220	3062	DB_56_U133_2.CEL	2	0	6	0	6	2
221	3063	DB_57_U133_2.CEL	1	0	0	0	0	6
222	3064	DB_58_U133_2.CEL	1	1	6	0	4	3
223	3078	HdT_3311_U133_2.CEL	5	4	0	0	3	1
224	3079	HdT_3296_U133_2.CEL	1	1	0	0	5	0
225	3084	DB_40_U133_2.CEL	10	10	0	0	1	1
226	3085	DB_42_U133_2.CEL	10	8	2	0	10	6
227	3099	HdT_3139_U133_2.CEL	1	1	2	0	0	8
228	3121	250706-15.CEL	0	0	0	0	0	9
229	3158	DB_11442_U133_2.CEL	0	0	2	0	0	10
230	3159	HdT_2570_U133_2.CEL	10	10	0	0	4	0
231	3165	HdT_2377_U133_2.CEL	3	4	5	0	2	1
232	3170	DB_11651_U133_2.CEL	9	7	0	0	0	1
233	3172	DB_11614_U133_2.CEL	7	2	7	0	5	7
234	3176	DB_17_U133_2.CEL	10	10	0	0	2	1
235	3209	DB_10797_U133_2.CEL	5	4	2	0	0	5
236	3242	GSM519129.CEL.gz	2	0	4	0	1	8
237	3251	GSM519138.CEL.gz	6	0	10	0	10	0
238	3257	GSM519144.CEL.gz	2	0	5	0	4	0
239	3270	GSM519157.CEL.gz	0	0	5	0	0	0
240	3281	GSM519168.CEL.gz	0	0	5	0	2	0
241	3296	GSM519183.CEL.gz	0	0	3	0	1	5
242	3300	GSM519187.CEL.gz	5	0	7	0	0	5
243	3301	GSM519188.CEL.gz	4	0	0	0	0	8
244	3303	GSM519190.CEL.gz	4	0	6	0	2	7
245	3337	GSM519224.CEL.gz	5	0	3	0	8	0
246	3350	GSM519237.CEL.gz	6	6	1	0	0	9
247	3379	GSM519266.CEL.gz	0	0	5	0	1	1
248	3381	GSM519268.CEL.gz	3	10	0	0	0	0
249	3394	GSM519281.CEL.gz	0	0	0	0	0	5
250	3400	GSM519287.CEL.gz	0	1	1	0	8	0
251	3476	GSM519363.CEL.gz	0	0	0	0	9	0
252	3499	GSM519386.CEL.gz	0	0	3	0	7	0
253	3502	GSM519389.CEL.gz	0	0	4	0	7	0
254	3529	GSM519416.CEL.gz	7	3	2	0	4	1
255	3531	GSM519418.CEL.gz	5	0	1	0	1	0
256	3532	GSM519419.CEL.gz	6	0	9	0	6	0
257	3535	GSM519422.CEL.gz	8	0	1	0	5	0
258	3542	GSM519429.CEL.gz	9	1	0	0	0	0
259	3543	GSM519430.CEL.gz	10	7	0	0	0	0
260	3544	GSM519431.CEL.gz	10	10	0	0	0	0
261	3545	GSM519432.CEL.gz	10	1	0	0	0	0
262	3547	GSM519434.CEL.gz	10	9	0	0	0	0
263	3548	GSM519435.CEL.gz	6	8	0	0	0	0
264	3552	GSM519439.CEL.gz	6	0	0	0	0	0
265	3553	GSM519440.CEL.gz	2	9	0	0	0	0
266	3554	GSM519441.CEL.gz	10	10	0	0	0	0
267	3555	GSM519442.CEL.gz	1	10	0	0	0	0
268	3556	GSM519443.CEL.gz	3	10	0	0	1	0
269	3559	GSM491177.CEL.gz	1	0	1	0	0	5
270	3581	GSM491199.CEL.gz	0	0	3	0	9	4
271	3587	GSM491205.CEL.gz	2	6	0	0	0	0
272	3594	GSM491212.CEL.gz	0	0	3	0	6	0
273	3608	GSM491226.CEL.gz	0	0	0	0	7	0
274	3664	GSM491282.CEL.gz	0	0	0	0	7	0
275	3675	GSM124997.CEL.gz	7	2	1	0	0	5
276	3698	GSM125020.CEL.gz	2	0	5	0	10	4
277	3705	GSM125027.CEL.gz	7	4	0	0	0	0
278	3802	GSM85476.CEL.gz	0	0	6	0	0	0
279	3807	GSM85481.CEL.gz	0	0	0	0	7	0
280	3808	GSM85482.CEL.gz	0	0	10	0	10	9
281	3810	GSM85484.CEL.gz	0	0	5	0	7	0
282	3823	GSM85497.CEL.gz	0	0	6	0	9	0
283	3865	GSM467542.CEL.gz	7	2	0	0	0	0
284	3867	GSM467544.CEL.gz	8	0	0	0	0	0
285	3868	GSM467545.CEL.gz	8	0	0	0	0	0
286	3870	GSM467547.CEL.gz	8	6	0	0	0	0
287	3885	GSM467562.CEL.gz	9	1	8	0	10	8
288	3889	GSM467566.CEL.gz	10	8	0	0	6	1
289	3898	GSM467575.CEL.gz	10	10	10	0	10	10
290	3902	GSM467579.CEL.gz	4	8	0	0	0	0
291	3914	GSM467591.CEL.gz	10	7	0	0	0	3
292	3930	GSM540108_160306-23.CEL.gz	10	9	10	0	9	10
293	3931	GSM540109_060406-05.CEL.gz	10	10	10	0	10	10
294	3932	GSM540110_200406-16.CEL.gz	10	9	10	0	10	10
295	3938	GSM540116_160302-01.CEL.gz	2	0	5	0	2	0
296	3952	GSM540130_160302-02.CEL.gz	1	0	0	0	0	10
297	3961	GSM540139_080414-04.CEL.gz	8	9	10	0	10	10
298	3963	GSM540141_090905-02.CEL.gz	10	10	10	0	10	10
299	3970	GSM540148_090205-23.CEL.gz	0	0	1	0	1	8
300	4009	GSM540187_080318-06.CEL.gz	0	0	6	0	0	0
301	4016	GSM540194_270905-10.CEL.gz	1	5	0	0	0	0
302	4017	GSM540195_260106-08.CEL.gz	5	4	1	0	1	6
303	4023	GSM540201_260106-07.CEL.gz	0	0	0	0	0	7
304	4036	GSM540214_260106-06_2nd_scan_taches.CEL.gz	10	10	10	0	9	9
305	4053	GSM540231_071205-05.CEL.gz	10	10	10	0	10	10

Table S7. Overview of the 319 hybridizations rejected based on QC. Continued on next page.

Index	ID	CEL	NUSE	GNUSE	RLE	MA-plot	Boxplot	Heatmap
306	4139	GSM540317_15719_T1.CEL.gz	8	10	10	0	10	0
307	4140	GSM540318_15724_T2.CEL.gz	7	10	8	0	2	0
308	4141	GSM540319_15744_T7.CEL.gz	6	9	10	0	10	0
309	4143	GSM540321_15765_T9.CEL.gz	0	8	3	0	1	0
310	4144	GSM540322_15986_T24.CEL.gz	9	10	10	0	9	0
311	4145	GSM540323_16137_T39.CEL.gz	6	9	10	0	9	0
312	4146	GSM540324_16325_T56.CEL.gz	4	10	10	0	10	0
313	4147	GSM540325_17231_T125.CEL.gz	2	7	10	0	8	3
314	4154	GSM540332_090115-08.CEL.gz	6	6	0	0	9	0
315	4165	GSM540343_090129-01.CEL.gz	0	0	1	0	2	10
316	4198	GSM441382.CEL.gz	0	0	0	0	0	10
317	4214	GSM441366.CEL.gz	10	0	0	0	0	0
318	4216	GSM441368.CEL.gz	0	10	8	0	10	0
319	4220	GSM441372.CEL.gz	0	0	1	0	9	0

Table S7. Overview of the 319 hybridizations rejected based on QC. ID: short array identifier used in the QC overview figures, pages 12-23; CEL: the original CEL file name. This frequently equates to the GSM accession number from GEO extended with '.CEL.gz'. The remaining six columns indicate in how many of $R = 10$ QC repeats the array was flagged for each of the six QC indicators NUSE, GNUSE, RLE, MA-plot, boxplot and heatmap.

References

[1] McCall, M.N., Bolstad, B.M., Irizarry, R.A.: Frozen robust multiarray analysis (fRMA). Biostatistics **11**(2), 242–253 (2010)

[2] McCall, M., Irizarry, R.: Thawing frozen robust multi-array analysis (fRMA). BMC Bioinformatics **12**, 369 (2011)

[3] Guedj, .M., Marisa, .L., De Reynies, .A., Orsetti, .B., Schiappa, .R., Bibeau, .F., MacGrogan, .G., Lerebours, .F., Finetti, .P., Longy, .M., Bertheau, .P., *et al.*: A refined molecular taxonomy of breast cancer. Oncogene **31**(9), 1196–1206 (2012)

[4] Kauffmann, A., Huber, W.: Microarray data quality control improves the detection of differentially expressed genes. Genomics **95**(3), 138–142 (2010)

[5] McCall, M.N., Murakami, P.N., Lukk, M., Huber, W., Irizarry, R.A.: Assessing Affymetrix GeneChip microarray quality. BMC Bioinformatics **12**, 137 (2011)

[6] Bolstad, B.M.: Low-level analysis of high-density oligonucleotide array data: background, normalization and summarization. PhD thesis, , University of California (2004)

[7] Leek, J.T., Scharpf, R.B., Bravo, H.C., Simcha, D., Langmead, B., Johnson, W.E., Geman, D., Baggerly, K., Irizarry, R.A.: Tackling the widespread and critical impact of batch effects in high-throughput data. Nature Reviews Genetics **11**(10), 733–739 (2010)

[8] Edgar, R., Domrachev, M., Lash, A.E.: Gene Expression Omnibus: NCBI gene expression and hybridization array data repository. Nucleic Acids Research **30**(1), 207–210 (2002)

[9] Friedman, J., Hastie, T., Tibshirani, R.: The Elements of Statistical Learning: Data Mining, Inference, and Prediction, 1st edn. Springer, New York, NY (2001)

[10] Hu, Z., Fan, C., Oh, D.S., Marron, J., He, X., Qaqish, B.F., Livasy, C., Carey, L.A., Reynolds, E., Dressler, L., *et al.*: The molecular portraits of breast tumors are conserved across microarray platforms. BMC Genomics **7**, 96 (2006)

[11] Parker, J.S., Mullins, M., Cheang, M.C.U., Leung, S., Voduc, D., Vickery, T., Davies, S., Fauron, C., He, X., Hu, Z., *et al.*: Supervised risk predictor of breast cancer based on intrinsic subtypes. Journal of Clinical Oncology **27**(8), 1160–1167 (2009)

[12] Sørlie, T., Tibshirani, R., Parker, J., Hastie, T., Marron, J., Nobel, A., Deng, S., Johnsen, H., Pesich, R., Geisler, S., *et al.*: Repeated observation of breast tumor subtypes in independent gene expression data sets. Proceedings of the National Academy of Sciences **100**(14), 8418–8423 (2003)

[13] Desmedt, C., Haibe-Kains, B., Wirapati, P., Buyse, M., Larsimont, D., Bontempi, G., Delorenzi, M., Piccart, M., Sotiriou, C.: Biological processes associated with breast cancer clinical outcome depend on the molecular subtypes. Clinical Cancer Research **14**(16), 5158–5165 (2008)

[14] Haibe-Kains, B., Desmedt, C., Loi, S., Culhane, A.C., Bontempi, G., Quackenbush, J., Sotiriou, C.: A three-gene model to robustly identify breast cancer molecular subtypes. Journal of the National Cancer Institute **104**(4), 311–325 (2012)

[15] Wirapati, P., Sotiriou, C., Kunkel, S., Farmer, P., Pradervand, S., Haibe-Kains, B., Desmedt, C., *et al.*: Meta-analysis of gene expression profiles in breast cancer: toward a unified understanding of breast cancer subtyping and prognosis signatures. Breast Cancer Research **10**(4), 65 (2008)

[16] Goldhirsch, A., Wood, W., Coates, A., Gelber, R., Thürlimann, B., Senn, H.J., *et al.*: Strategies for subtypes - dealing with the diversity of breast cancer: highlights of the St Gallen International Expert Consensus on the Primary Therapy of Early Breast Cancer 2011. Annals of Oncology **22**(8), 1736–1747 (2011)

[17] Wang, J., Wen, S., Symmans, W.F., Pusztai, L., Coombes, K.R.: The bimodality index: a criterion for discovering and ranking bimodal signatures from cancer gene expression profiling data. Cancer Informatics **7**, 199–216 (2009)

[18] Nielsen, T.O., Parker, J.S., Leung, S., Voduc, D., Ebbert, M., Vickery, T., Davies, S.R., Snider, J., Stijleman, I.J., Reed, J., *et al.*: A comparison of PAM50 intrinsic subtyping with immunohistochemistry and clinical prognostic factors in tamoxifen-treated estrogen receptor–positive breast cancer. Clinical Cancer Research **16**(21), 5222–5232 (2010)

[19] Tibshirani, R., Walther, G.: Cluster validation by prediction strength. Journal of Computational and Graphical Statistics **14**(3), 511–528 (2005)

[20] Lusa, L., McShane, L.M., Reid, J.F., De Cecco, L., Ambrogi, F., Biganzoli, E., Gariboldi, M., Pierotti, M.A.: Challenges in projecting clustering results across gene expression profiling datasets. Journal of the National Cancer Institute **99**(22), 1715–1723 (2007)

[21] Pusztai, L., Mazouni, C., Anderson, K., Wu, Y., Symmans, W.F.: Molecular classification of breast cancer: limitations and potential. The Oncologist **11**(8), 868–877 (2006)
