# Supplementary material for: Breast cancer subtype predictors revisited: from consensus to concordance?
Source: BMC Med Genomics. 2016 Jun 3;9:26. doi: 10.1186/s12920-016-0185-6 (PMC4893290; doi:10.1186/s12920-016-0185-6)
Supplement: Additional file 2 — Supplementary figures and tables. (PDF 543 kb) [file 12920_2016_185_MOESM2_ESM.pdf]

Additional File 2: Supplementary Figures and Tables

Breast Cancer Subtype Predictors Revisited: From Consensus to Concordance?

Herman M.J. Sontrop[1,2], Marcel J.T. Reinders[3], Perry D. Moerland[4,*]
1 Molecular Diagnostics Department, Philips Research, High Tech Campus 11, 5656 AE Eindhoven, The Netherlands
2 Friss Fraud and Risk Solutions, Orteliuslaan 15, 3528 BA, Utrecht, The Netherlands
3 Delft Bioinformatics Lab, Delft University of Technology, Mekelweg 4, 2628 CD Delft, The Netherlands
4 Bioinformatics Laboratory, Academic Medical Center, Meibergdreef 9, 1105 AZ Amsterdam, The Netherlands
∗ Corresponding author, e-mail: p.d.moerland@amc.uva.nl, phone: ++31 205666945

Panel	Index	Description	Type	κ (Weigelt)	κ (133plus2)	κ (all)	$\Delta\kappa$
All	1	weigelt uncentered	SSP.classic	0.467	-	-	-
	2	weigelt centered	SSP.classic	0.561	-	-	-
	3	SSP.classic.4s	SSP.classic	-	0.560	0.555	-0.005
	4	SCM.classic	SCM.classic	-	0.778	0.772	-0.006
	5	different CS and GL	SSP.cs	-	0.828	0.800	-0.028
	6	same CS, different GL	SSP.cs	-	0.854	0.820	-0.034
	7	same GL, different CS	SSP.cs	-	0.914	0.905	-0.009
	8	different CS and GL	SCM.cs	-	0.778	0.753	-0.025
	9	same CS, different GL	SCM.cs	-	0.814	0.776	-0.038
	10	same GL, different CS	SCM.cs	-	0.876	0.872	-0.004
Basal	1	weigelt uncentered	SSP.classic	0.921	-	-	-
	2	weigelt centered	SSP.classic	0.852	-	-	-
	3	SSP.classic.4s	SSP.classic	-	0.927	0.921	-0.006
	4	SCM.classic	SCM.classic	-	0.867	0.867	0.000
	5	different CS and GL	SSP.cs	-	0.948	0.937	-0.011
	6	same CS, different GL	SSP.cs	-	0.948	0.940	-0.008
	7	same GL, different CS	SSP.cs	-	0.987	0.979	-0.008
	8	different CS and GL	SCM.cs	-	0.906	0.892	-0.014
	9	same CS, different GL	SCM.cs	-	0.907	0.892	-0.015
	10	same GL, different CS	SCM.cs	-	0.987	0.982	-0.005
HER2	1	weigelt uncentered	SSP.classic	0.482	-	-	-
	2	weigelt centered	SSP.classic	0.564	-	-	-
	3	SSP.classic.4s	SSP.classic	-	0.502	0.480	-0.022
	4	SCM.classic	SCM.classic	-	0.762	0.795	+0.033
	5	different CS and GL	SSP.cs	-	0.808	0.798	-0.010
	6	same CS, different GL	SSP.cs	-	0.846	0.832	-0.014
	7	same GL, different CS	SSP.cs	-	0.916	0.901	-0.015
	8	different CS and GL	SCM.cs	-	0.756	0.754	-0.002
	9	same CS, different GL	SCM.cs	-	0.768	0.768	0.000
	10	same GL, different CS	SCM.cs	-	0.926	0.919	-0.007
LumA	1	weigelt uncentered	SSP.classic	0.449	-	-	-
	2	weigelt centered	SSP.classic	0.508	-	-	-
	3	SSP.classic.4s	SSP.classic	-	0.546	0.546	0.000
	4	SCM.classic	SCM.classic	-	0.766	0.757	-0.009
	5	different CS and GL	SSP.cs	-	0.808	0.776	-0.032
	6	same CS, different GL	SSP.cs	-	0.831	0.794	-0.037
	7	same GL, different CS	SSP.cs	-	0.904	0.897	-0.007
	8	different CS and GL	SCM.cs	-	0.750	0.732	-0.018
	9	same CS, different GL	SCM.cs	-	0.788	0.761	-0.027
	10	same GL, different CS	SCM.cs	-	0.840	0.839	-0.001
LumB	1	weigelt uncentered	SSP.classic	0.306	-	-	-
	2	weigelt centered	SSP.classic	0.244	-	-	-
	3	SSP.classic.4s	SSP.classic	-	0.276	0.311	+0.035
	4	SCM.classic	SCM.classic	-	0.701	0.678	-0.023
	5	different CS and GL	SSP.cs	-	0.738	0.715	-0.023
	6	same CS, different GL	SSP.cs	-	0.774	0.737	-0.037
	7	same GL, different CS	SSP.cs	-	0.857	0.857	0.000
	8	different CS and GL	SCM.cs	-	0.691	0.668	-0.023
	9	same CS, different GL	SCM.cs	-	0.736	0.702	-0.034
	10	same GL, different CS	SCM.cs	-	0.797	0.802	+0.005

Table S1. Intra-predictor concordance of SSPs and SCMs. Numerical details of Figure 3 in the main text. *Panel*: panel indicator; *Index*: box and whisker plot index per panel, starting from the top; *Description*: predictor pair description; *Type*: subtype predictor type; κ *(Weigelt)*: median kappa statistics based on the published subtype assignments by Weigelt et al. [1,2]; κ *(133plus2)*: median kappa statistics computed over all 2,019 Affymetrix hgu133plus2 arrays (Table 1, main text); κ *(all)*: median kappa statistics computed over all 3,908 samples in the Affymetrix compendium, i.e. including the hgu133a samples; $\Delta\kappa$: difference between the median kappa statistics computed on hgu133plus2 samples and on all samples. A negative value indicates a decrease in concordance when the hgu133a samples are included.

	Weigelt uncentered	Weigelt centered	SSP.classic	SSP.classic.4s	SCM.classic	SSP.cs	SCM.cs
cc (all, %)	61.72	66.59	70.75 (71.26)	66.97 (67.01)	83.88 (83.66)	90.32 (88.99)	86.67 (85.33)
κ (all)	0.467	0.561	0.575 (0.570)	0.560 (0.555)	0.778 (0.772)	0.865 (0.842)	0.812 (0.791)
κ (basal)	0.921	0.852	0.914 (0.904)	0.927 (0.921)	0.867 (0.867)	0.970 (0.958)	0.930 (0.922)
κ (HER2)	0.482	0.564	0.500 (0.470)	0.502 (0.480)	0.762 (0.795)	0.846 (0.841)	0.802 (0.793)
κ (lumA)	0.449	0.508	0.642 (0.624)	0.546 (0.546)	0.766 (0.757)	0.845 (0.817)	0.778 (0.766)
κ (lumB)	0.306	0.244	0.276 (0.308)	0.276 (0.311)	0.701 (0.678)	0.780 (0.763)	0.731 (0.712)

Table S2. Summary of intra-predictor concordance of SSPs and SCMs. Numerical details of Figure 3 in the main text: median percentage of concordant samples (cc) and median kappa statistics. Results presented in the first two columns were obtained using the published subtype assignments by Weigelt et al. on uncentered [1] and centered data [2], respectively. Remaining columns summarize results based on our hgu133plus2 compendium and - between parentheses - on the entire Affymetrix compendium, i.e. including the hgu133a arrays (Table 1, main text). For the CS-based predictors (SSP.cs, SCM.cs) the entries are pooled estimates corresponding to the vertical gray lines in Figure 3 in the main text (spanning 3 rows within each panel).

	Single SSP: centered vs. uncentered data				Pair of SSPs: centered data only				
	SSP	All	Single	Dual	SSP.1	SSP.2	All	Single	Dual
cc (all, %)	Sørlie	66.71	58.29	82.03	Sørlie	Hu	63.58	64.80	61.36
	Hu	68.87	63.50	78.64	Sørlie	PAM50	64.06	65.18	62.03
	PAM50	75.00	67.23	89.15	Hu	PAM50	72.36	72.25	72.54
κ (all)	Sørlie	0.570	0.462	0.766	Sørlie	Hu	0.528	0.541	0.504
	Hu	0.585	0.504	0.722	Sørlie	PAM50	0.532	0.544	0.510
	PAM50	0.677	0.575	0.861	Hu	PAM50	0.644	0.639	0.652
κ (basal)	Sørlie	0.879	0.840	0.962	Sørlie	Hu	0.792	0.813	0.744
	Hu	0.805	0.769	0.878	Sørlie	PAM50	0.894	0.899	0.882
	PAM50	0.903	0.902	0.905	Hu	PAM50	0.800	0.821	0.753
κ (HER2)	Sørlie	0.550	0.306	0.834	Sørlie	Hu	0.469	0.473	0.462
	Hu	0.650	0.564	0.820	Sørlie	PAM50	0.590	0.580	0.606
	PAM50	0.646	0.399	0.909	Hu	PAM50	0.584	0.541	0.650
κ (lumA)	Sørlie	0.648	0.573	0.779	Sørlie	Hu	0.518	0.520	0.516
	Hu	0.516	0.454	0.638	Sørlie	PAM50	0.443	0.458	0.417
	PAM50	0.664	0.560	0.854	Hu	PAM50	0.629	0.627	0.634
κ (lumB)	Sørlie	0.330	0.266	0.499	Sørlie	Hu	0.197	0.200	0.194
	Hu	0.538	0.376	0.741	Sørlie	PAM50	0.217	0.197	0.252
	PAM50	0.554	0.432	0.852	Hu	PAM50	0.624	0.611	0.645
κ (normal)	Sørlie	0.412	0.073	0.793	Sørlie	Hu	0.581	0.592	0.564
	Hu	0.289	0.028	0.543	Sørlie	PAM50	0.493	0.532	0.430
	PAM50	0.630	0.529	0.749	Hu	PAM50	0.500	0.457	0.557
Nr. of samples	-	832	537	295	-	-	832	537	295

Table S3. Concordance comparison: centering vs. difference in classic SSP. Percentage of concordant samples (cc) and kappa statistics calculated using subtype assignments by Weigelt et al. [1, 2]. The left-hand half of the table lists the concordance for a single classic SSP between assignments based on uncentered data and on centered data. In the right-hand half all comparisons were based on centered data for pairs of classic SSPs. The columns *All*, *Single* and *Dual* indicate results that were computed on all samples, only samples from single-channel experiments or only samples from dual-channel experiments, respectively. A lack of data centering in single-channel datasets leads to a strong increase of the correlation to the luminal B centroid [3]. Furthermore, correlations to the normal-like group are strongly decreased, in some cases to such an extent that the normal-like subtype can no longer be detected (Figure S1).

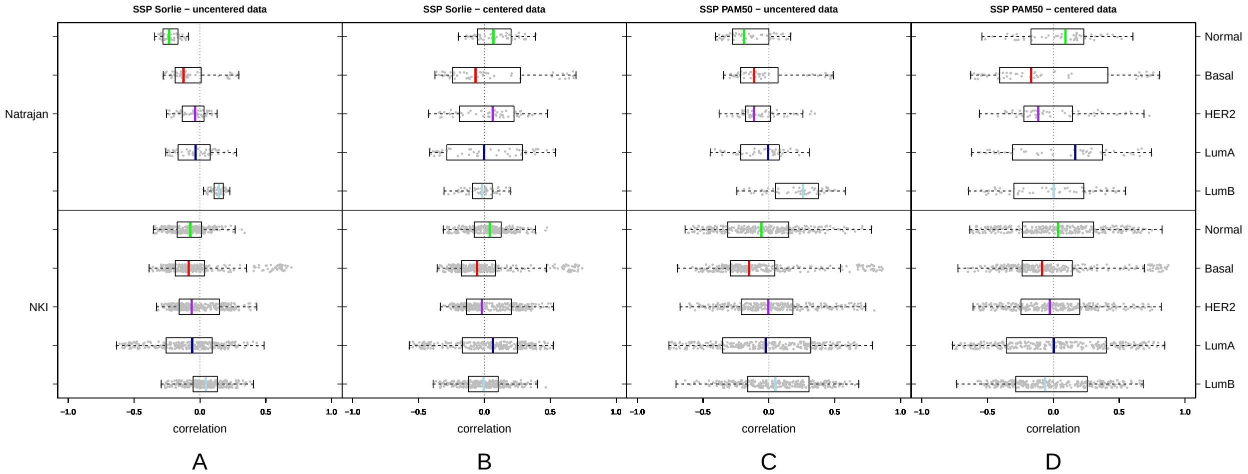

Figure S1. Example of impact of uncentered data in analysis of Weigelt and colleagues. Correlation between individual samples and each of the five subtype centroids of two classic SSPs (Sørlie and PAM50) for two datasets (Natrajan and NKI) analyzed in Weigelt et al. [1]. Correlations are shown for the SSP of Sørlie based on uncentered data (**A**), for the SSP of Sørlie based on centered data (**B**), for the PAM50 subtype predictor on uncentered data (**C**) and for PAM50 on centered data (**D**). The upper half (Natrajan) in each panel shows correlations for the single-channel dataset of Natrajan et al. [4] (ArrayExpress: E-TABM-543), while the lower half (NKI) shows correlations for the dual-channel dataset of Van de Vijver et al. [5]. Correlations were computed with the expression data as used by Weigelt et al. [1]. A related but different view on the same data is offered in the supplementary web appendix of the reaction by Sørlie et al. [3].

In Panel (**A**) The two BW plots corresponding to the normal-like and luminal B centroid for the Natrajan dataset are completely non-overlapping. This implies that in this case the normal-like subtype will never be detected, since for each sample the correlation to the luminal B centroid is stronger. Furthermore, in the same panel one can observe that the range of the BW plots for each centroid is fairly small. In Panel (**B**), we see that when the data is properly scaled the BW plots are wider and more centered and hence they do not directly imply the exclusion of detection of a subtype. The lower halfs in panel (**A**) and (**B**) show the results for a dual-channel data set of log-ratio data from a common reference design. The impact of data centering is fairly small in this case. Comparing panel (**C**) to panel (**A**) we see that even when using uncentered data, the ranges obtained by PAM50 are wider than for the SSP of Sørlie, while for centered data (panel (**D**)) they are wider yet. In addition, the BW plots associated with PAM50 show much higher correlations compared to the SSP of Sørlie which suggests a larger confidence in subtype detection.

	Weigelt (centered)	Haibe-Kains	Guedj	Perou
cc (%, Sørlie vs. Hu)	63.58	69.25	58.24	
cc (%, Sørlie vs. PAM50)	64.06	60.13	57.64	
cc (%, Hu vs. PAM50)	72.36	73.64	75.74	77.60
κ (all, Sørlie vs. Hu)	0.528	0.581	0.464	
κ (all, Sørlie vs. PAM50)	0.532	0.474	0.460	
κ (all, Hu vs. PAM50)	0.644	0.652	0.690	0.710
κ (basal, Sørlie vs. Hu)	0.792	0.906	0.692	
κ (basal, Sørlie vs. PAM50)	0.894	0.907	0.750	
κ (basal, Hu vs. PAM50)	0.800	0.906	0.771	0.856
κ (HER2, Sørlie vs. Hu)	0.469	0.430	0.347	
κ (HER2, Sørlie vs. PAM50)	0.590	0.556	0.420	
κ (HER2, Hu vs. PAM50)	0.584	0.700	0.648	0.722
κ (lumA, Sørlie vs. Hu)	0.518	0.617	0.456	
κ (lumA, Sørlie vs. PAM50)	0.443	0.372	0.396	
κ (lumA, Hu vs. PAM50)	0.629	0.606	0.704	0.673
κ (lumB, Sørlie vs. Hu)	0.197	0.192	0.275	
κ (lumB, Sørlie vs. PAM50)	0.217	0.196	0.257	
κ (lumB, Hu vs. PAM50)	0.624	0.476	0.633	0.618
κ (normal, Sørlie vs. Hu)	0.581	0.541	0.540	
κ (normal, Sørlie vs. PAM50)	0.493	0.314	0.530	
κ (normal, Hu vs. PAM50)	0.500	0.451	0.682	0.588
Nr. of samples	832	2576	2828	442

Table S4. Lack of concordance of classic SSPs based on published subytpe assignments. Percentage of concordant samples (cc) and kappa statistics for pairs of classic SSPs, based on published subtype assignments from four research groups. The top row indicates the different sources from which subtype assignments were obtained. *Weigelt (centered)*: subtype assignments from the rebuttal by Weigelt et al. [2]; *Haibe-Kains*: subtype assignments by Haibe-Kains et al. [6]; *Guedj*: subtype assignments by Guedj et al. [7]; *Perou*: subtype assignments from the Perou group, as listed on the UNC website (`https://genome.unc.edu/pubsup/breastGEO/`). All subtype assignments were based on scaled expression data. Most experiments were based on median-centered data, however, Haibe-Kains et al. [6] relied on robust scaling (Methods, main text). Subtype assignments by the Perou group were only available for the SSP of Hu and PAM50.

Platform	All	Others	Affy	All	Others	Affy	All	Others	Affy
SCM.1	HK	HK	HK	HK	HK	HK	D	D	D
SCM.2	D	D	D	W	W	W	W	W	W
cc (all, %)	74.83	65.72	**81.91**	78.01	69.45	**84.67**	85.66	82.40	**88.24**
κ (all)	0.662	0.542	**0.756**	0.703	0.590	**0.792**	0.807	0.763	**0.841**
κ (basal)	0.715	0.546	**0.860**	0.749	0.574	**0.898**	0.897	0.854	**0.930**
κ (HER2)	0.730	0.664	**0.787**	0.811	0.735	**0.877**	0.766	0.726	**0.800**
κ (lumA)	0.646	0.538	**0.716**	0.675	0.578	**0.741**	0.804	0.765	**0.831**
κ (lumB)	0.576	0.448	**0.677**	0.609	0.510	**0.685**	0.754	0.706	**0.794**
Nr. of samples	4606	2030	2576	4606	2030	2576	4606	2030	2576

Table S5. Impact of platform heterogeneity on SCM-based subtyping. Percentage of concordant samples (cc) and kappa statistics for pairs of classic SCMs. Individual subtype assignments as published in [6] were aggregated into a single vector and subsequently dichotomized into a set containing only assignments made on Affymetrix datasets and another set made on non-Affymetrix datasets. *SCM.1* and *SCM.2* indicate for each pair the classic SCMs involved (HK, D, W); *Platform* indicates the set of experiments on which levels of concordance were computed; *All* refers to all subtype assignments, irrespective of the corresponding measurement platform; *Affy* refers to subtype assignments based on data from the Affymetrix platform only; *Others* refers to subtype assignments for all non-Affymetrix platforms; *Nr. of samples* indicates the total number of arrays in each sub-analysis. Bold entries indicate the best performance for a given SCM pair.

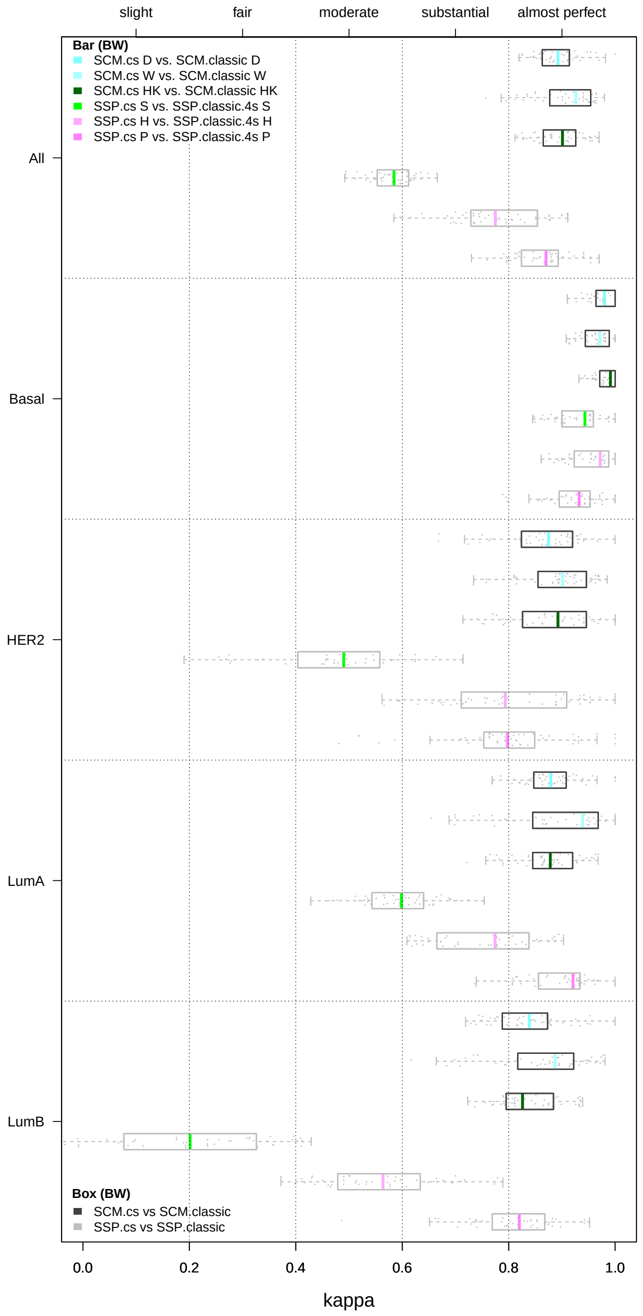

Figure S2. Concordance of CS-based models and their classic counterparts (hgu133plus2 compendium). The five panels show box and whisker (BW) plots for kappa statistics calculated over all subtypes and for each subtype separately, as indicated on the left hand side. Results for individual datasets are superimposed as dots. Each panel contains six BW plots. The upper three BW plots indicate concordance for SCM predictor pairs, whereas the lower three BW plots indicate concordance for SSP predictor pairs. For the classic predictors we used the *genefu* implementations, i.e. *ssp2003.robust* (SSP.classic.4s S), *ssp2006.robust* (SSP.classic.4s H), *pam50.robust* (SSP.classic.4s P), *scmod1.robust* (SCM.classic D), *scmod2.robust* (SCM.classic W) and *scmgene.robust* (SCM.classic HK). For each predictor pair, the CS version used the same gene list (IGL for SSPs, MGL for SCMs) as its classic counterpart. Results are based on the hgu133plus2 compendium. Top legend: composition of each pair indicated by the color of BW median values (indicated by a bar). Bottom legend: predictor type indicated by the color of a BW box. Numerical details of the BW plots are presented in Table S6.

	Description	hgu133plus2	All	Δ
cc (all, %)	SCM.cs D vs. SCM.classic D	92.15	92.13	-0.02
	SCM.cs W vs. SCM.classic W	94.55	94.42	-0.13
	SCM.cs HK vs. SCM.classic HK	92.89	92.42	-0.47
	SSP.cs S vs. SSP.classic S	70.24	70.08	-0.16
	SSP.cs H vs. SSP.classic H	83.95	82.22	-1.73
	SSP.cs P vs. SSP.classic P	90.77	90.20	-0.57
κ (all)	SCM.cs D vs. SCM.classic D	0.893	0.890	-0.003
	SCM.cs W vs. SCM.classic W	0.926	0.921	-0.005
	SCM.cs HK vs. SCM.classic HK	0.901	0.888	-0.013
	SSP.cs S vs. SSP.classic S	0.584	0.566	-0.018
	SSP.cs H vs. SSP.classic H	0.775	0.748	-0.027
	SSP.cs P vs. SSP.classic P	0.870	0.864	-0.006
κ (basal)	SCM.cs D vs. SCM.classic D	0.980	0.980	0
	SCM.cs W vs. SCM.classic W	0.971	0.961	-0.01
	SCM.cs HK vs. SCM.classic HK	0.991	0.986	-0.005
	SSP.cs S vs. SSP.classic S	0.943	0.926	-0.017
	SSP.cs H vs. SSP.classic H	0.972	0.946	-0.026
	SSP.cs P vs. SSP.classic P	0.933	0.927	-0.006
κ (HER2)	SCM.cs D vs. SCM.classic D	0.875	0.873	-0.002
	SCM.cs W vs. SCM.classic W	0.902	0.926	+0.024
	SCM.cs HK vs. SCM.classic HK	0.893	0.878	-0.015
	SSP.cs S vs. SSP.classic S	0.490	0.456	-0.034
	SSP.cs H vs. SSP.classic H	0.794	0.759	-0.035
	SSP.cs P vs. SSP.classic P	0.798	0.792	-0.006
κ (lumA)	SCM.cs D vs. SCM.classic.D	0.879	0.877	-0.002
	SCM.cs W vs. SCM.classic W	0.939	0.921	-0.018
	SCM.cs HK vs. SCM.classic HK	0.878	0.883	+0.005
	SSP.cs S vs. SSP.classic S	0.598	0.583	-0.015
	SSP.cs H vs. SSP.classic H	0.774	0.746	-0.028
	SSP.cs P vs. SSP.classic P	0.921	0.904	-0.017
κ (lumB)	SCM.cs D vs. SCM.classic D	0.838	0.837	-0.001
	SCM.cs W vs. SCM.classic W	0.887	0.887	0
	SCM.cs HK vs. SCM.classic HK	0.826	0.812	-0.014
	SSP.cs S vs. SSP.classic S	0.202	0.278	+0.076
	SSP.cs H vs. SSP.classic H	0.564	0.552	-0.012
	SSP.cs P vs. SSP.classic P	0.820	0.820	0

Table S6. Concordance of CS-based models and their classic counterparts (hgu133plus2 and hgu133a compendium). The upper six rows provide the concordance percentages between CS-based SSPs/SCMs and their classic counterparts. The remaining rows correspond to the median kappa statistics shown in Figure S2 (column *hgu133plus2*) and Figure S3 (column *All*). Δ: difference between median concordance percentages or median kappa statistics computed on hgu133plus2 samples and on the complete compendium, respectively.

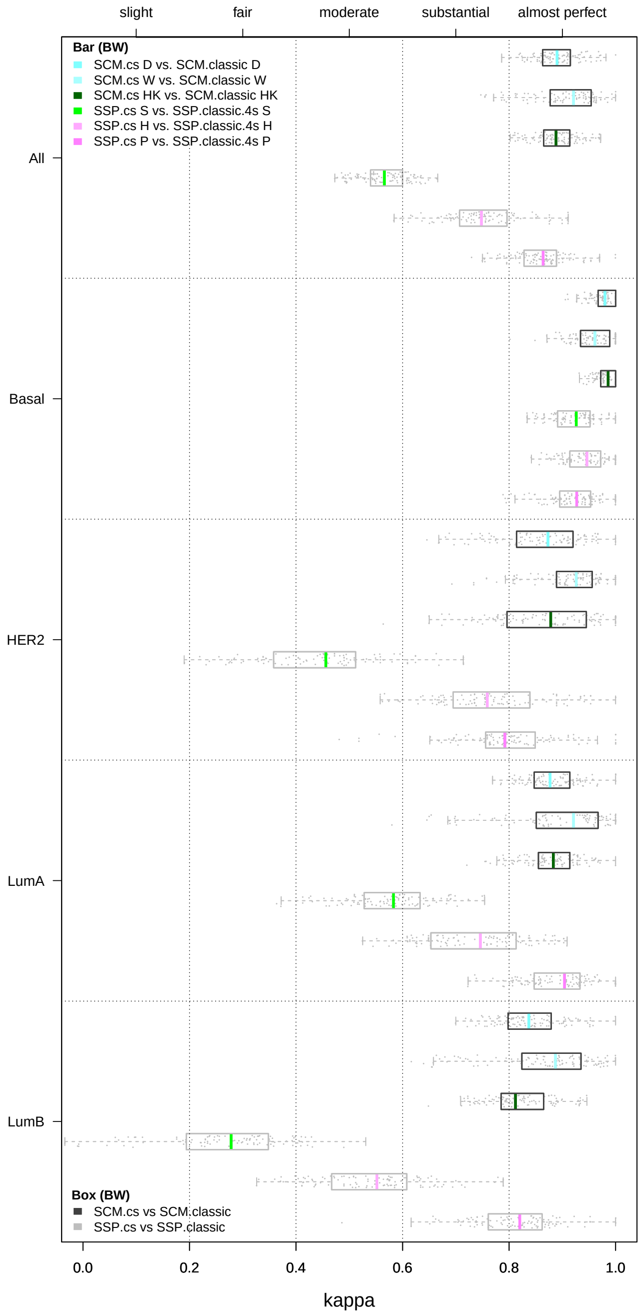

Figure S3. Concordance of CS-based models and their classic counterparts (hgu133plus2 and hgu133a compendium). Complement to Figure S2. The five panels show box and whisker (BW) plots for kappa statistics calculated over all subtypes and for each subtype separately, as indicated on the left hand side. Results for individual datasets are superimposed as dots. Each panel contains six BW plots. The upper three BW plots indicate concordance for SCM predictor pairs, whereas the lower three BW plots indicate concordance for SSP predictor pairs. For each predictor pair, the CS version used the same gene list (IGL for SSPs, MGL for SCMs) as its classic counterpart. Results are based on the entire Affymetrix compendium. Top legend: composition of each pair indicated by the color of BW median values (indicated by a bar). Bottom legend: predictor type indicated by the color of a BW box. Numerical details of the BW plots are presented in Table S6.

Description	cc (all, %)	κ (all)	κ (basal)	κ (HER2)	κ (lumA)	κ (lumB)
SSP.cs vs. SCM.cs	81.02 (80.69)	0.741 (0.734)	0.849 (0.847)	0.671 (0.657)	0.734 (0.728)	0.688 (0.663)
SSP.cs vs. STG.cs	80.07 (79.92)	0.729 (0.721)	0.826 (0.827)	0.599 (0.589)	0.755 (0.743)	0.682 (0.659)
SCM.cs vs. STG.cs	83.79 (83.32)	0.780 (0.770)	0.893 (0.893)	0.742 (0.745)	0.752 (0.750)	0.718 (0.666)
SCM.cs vs. STG.cs (same MGL)	89.84 (89.95)	0.861 (0.861)	0.953 (0.952)	0.811 (0.812)	0.853 (0.863)	0.811 (0.814)

Table S7. Summary of inter-predictor concordance of CS-based models. Numerical details of Figure 4 in the main text: median percentage of concordant samples (cc) and median kappa statistics between the SSP.cs, SCM.cs and STG.cs predictor pairs as indicated in the first column. Entries between parentheses represent results over the entire Affymetrix compendium, i.e. including the hgu133a arrays (Figure S4).

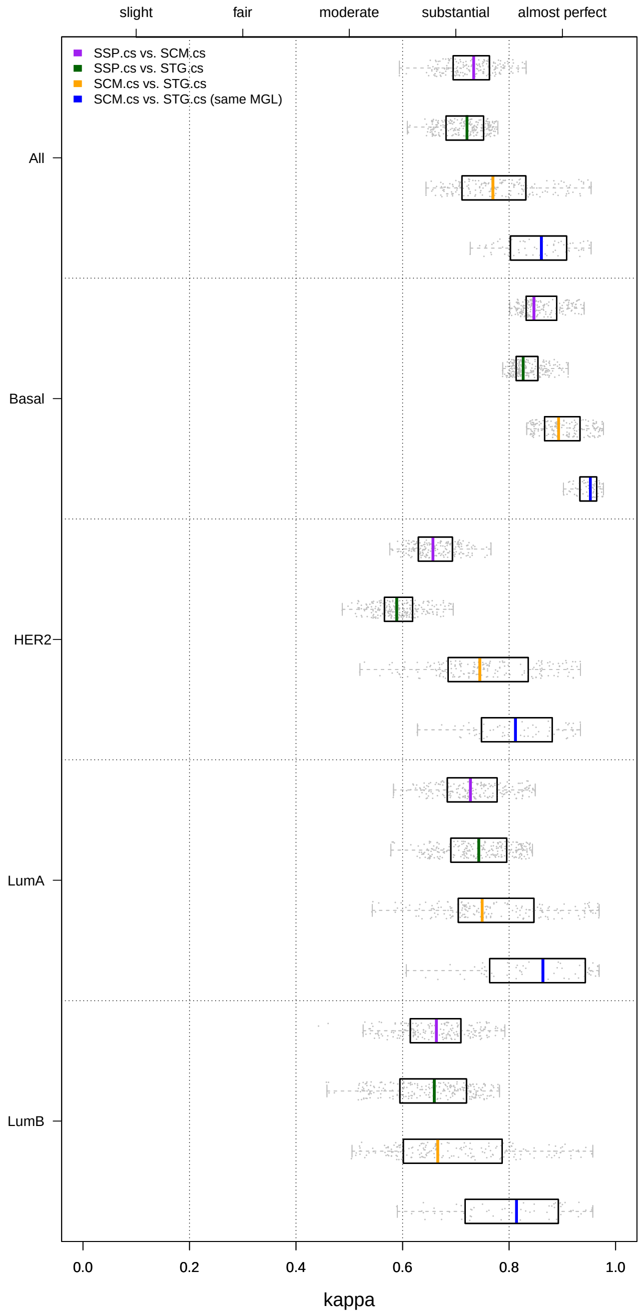

Figure S4. Inter-predictor concordance of CS-based models (hgu133plus2 and hgu133a compendium). Complement to Figure 4 in the main text. The five panels show box and whisker plots for kappa statistics calculated over all subtypes and for each each subtype separately, as indicated on the left hand side. Results for individual datasets are superimposed as dots. The upper three BW plots in each panel show the inter-predictor concordance estimates between the SSP.cs, SCM.cs and STG.cs predictors pairs, as indicated by the legend. The bottom BW plot in each panel provides the concordance estimates for SCM.cs and STG.cs predictor pairs when based on the same modules (with exception of PGR), i.e. MGLs. Results are based on the entire Affymetrix compendium. Numerical details of the BW plots are presented in Table S7.

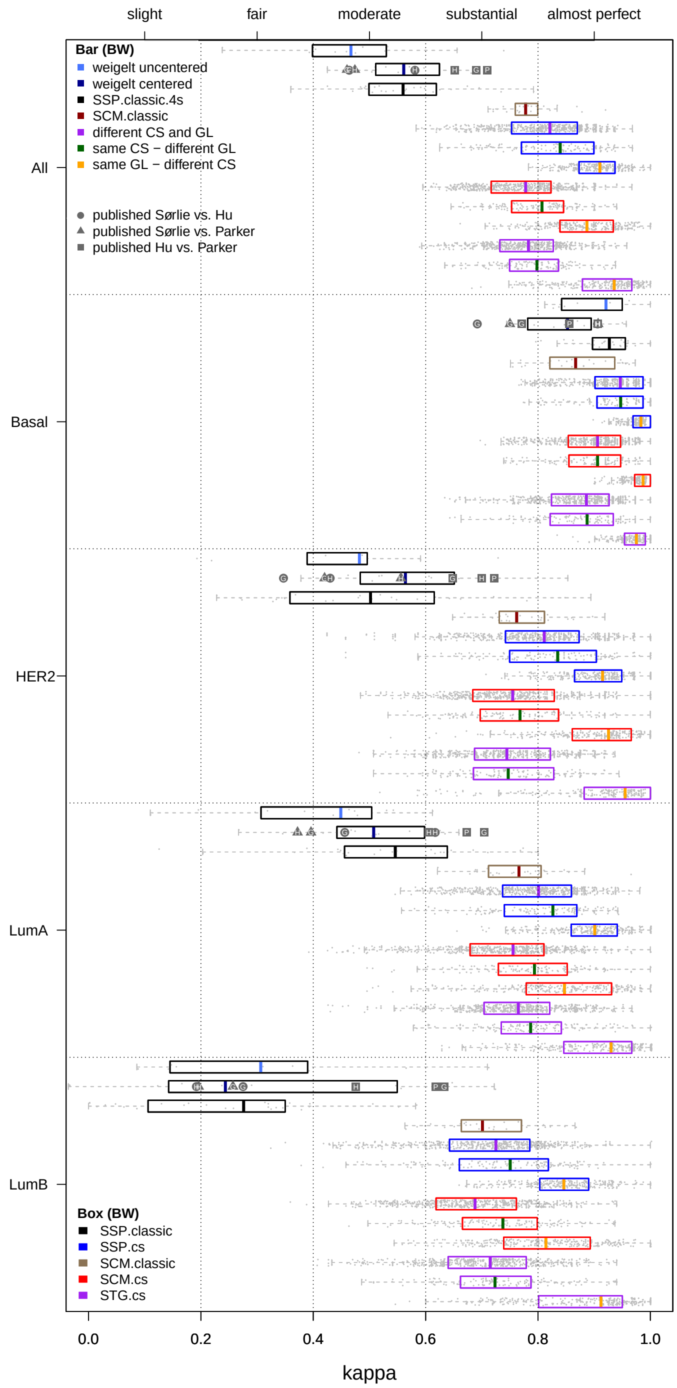

Figure S5. Intra-predictor concordance of SSPs, SCMs and STGs (hgu133plus2 compendium). Complement to Figure 3 in the main text. Also provides intra-STG.cs concordance estimates in addition to SSP and SCM intra-predictor concordance estimates. The modules used in the STG.cs model were identical to those used for the SCMs (with exception of PGR). Results were based on our hgu133plus2 compendium. See the legend of Figure 3 in the main text for more detailed information.

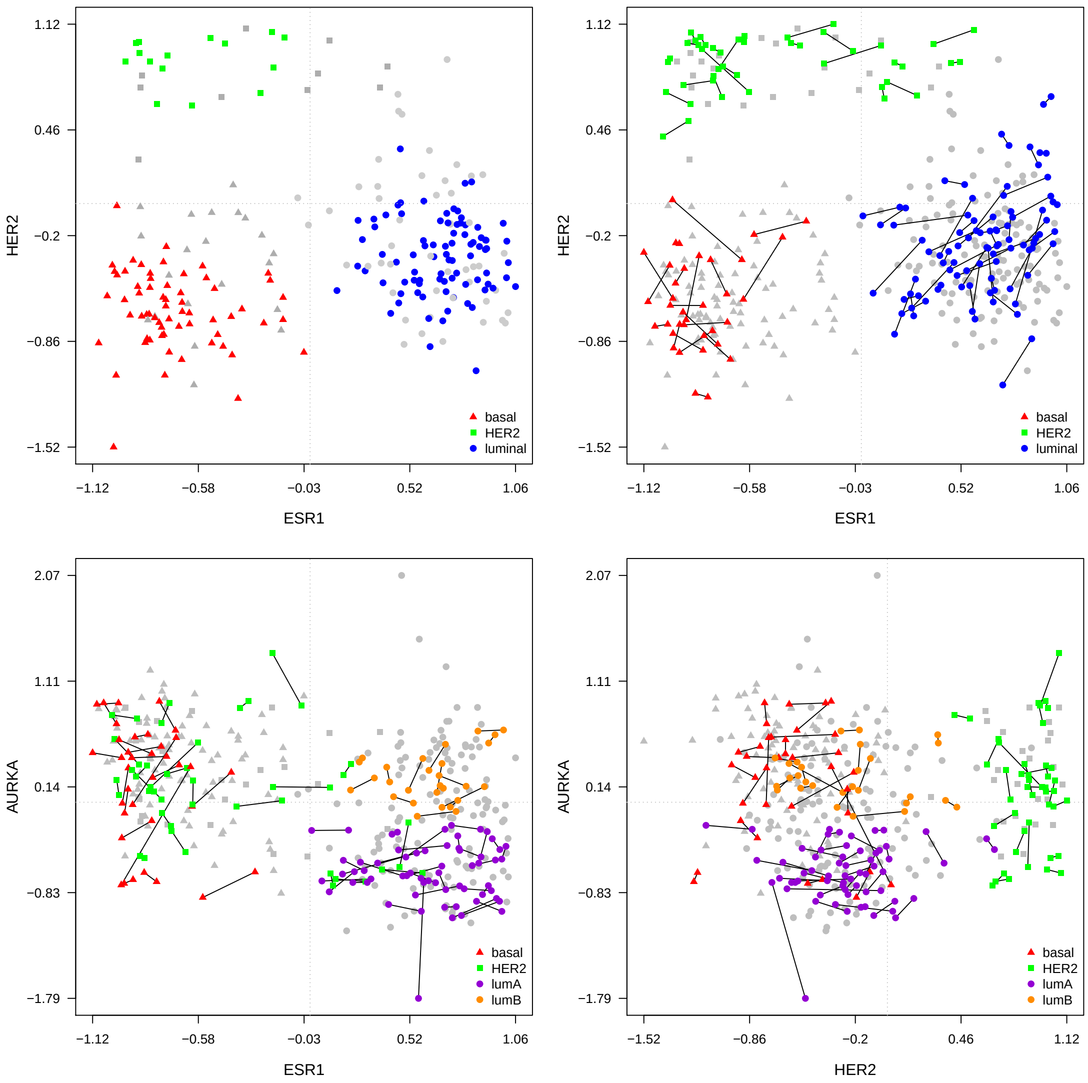

Figure S6. Concordance on replicate array pairs. SCM.cs HK was fitted on the 162 consensus set samples (Table 2, main text) from the Sabatier cohort (Dataset D9; Table 1, main text). The top left panel shows the module score scatterplot for the ESR1 and HER2 modules for all 266 samples from the Sabatier cohort. Consensus set samples are highlighted in color, whereas the remaining samples are shown in gray. The other panels show the module scores for a set of 93 replicate array pairs in our compendium. In these the module scores from the top left panel are shown in gray, while the module scores for the replicate arrays are highlighted in color. Module scores of replicate array pairs are connected by a line segment. In total 86 out of 93 replicate pairs were concordantly subtyped by this predictor (κ=0.90, cc=92.47%). For clarity only consistently subtyped pairs are shown.

Subset	cc (all, %)	κ (all)	κ (basal)	κ (HER2)	κ (lumA)	κ (lumB)
All	91.40	0.883	0.894	0.968	0.847	0.844
SSP.cs	91.40	0.883	0.881	0.969	0.859	0.868
SCM.cs	91.40	0.884	0.940	0.968	0.844	0.856

Table S8. Concordance on replicate array pairs. Percentage of concordant samples (cc) and kappa statistics for a set of 93 replicate array pairs in our compendium. Concordance values are median values over all pairs of CS-based predictors (*All*), SSP.cs predictors and SCM.cs predictors, respectively.

	ER HK	ER D	ER W	HER2 HK	HER2 D	HER2 W	PGR	Proliferation	AURKA HK	AURKA D	AURKA W
Bos	2.44	1.97	1.86	1.80	1.07	2.03	1.69	0.75	0.61	1.05	1.02
Dedeurwaerder	2.43	1.97	2.09	2.65	1.29	2.23	2.15	1.24	1.03	1.14	1.12
Desmedt	2.62	1.90	1.85	1.31	0.79	1.61	1.37	1.22	1.20	1.30	1.38
expO	2.39	1.37	1.53	1.46	1.12	1.59	1.57	1.36	1.45	1.36	1.29
Farmer	2.95	2.13	1.77	3.41	1.37	2.27	1.92	1.17	1.32	1.17	1.07
Guedj	2.12	1.49	1.49	1.45	0.89	1.65	1.73	0.91	1.14	1.01	0.91
Kao	1.88	1.33	1.47	1.76	1.07	1.70	1.43	1.08	1.16	1.16	1.10
Li	3.12	2.01	2.02	1.16	1.05	1.56	1.52	1.50	1.35	1.21	1.24
Lu	3.26	2.16	2.14	1.24	1.02	1.49	1.35	1.53	1.41	1.41	1.44
Miller	2.10	1.25	1.26	1.78	1.12	1.83	1.44	1.42	1.19	1.45	1.39
MSK	2.75	1.88	2.03	1.30	1.24	1.71	1.29	0.95	1.43	1.24	1.03
Pawitan	1.88	1.41	1.46	1.18	1.08	1.34	1.11	1.46	1.40	1.36	1.28
Richardson (I)	2.24	1.69	1.43	1.64	1.13	1.99	1.93	1.55	1.38	1.45	1.50
Richardson (II)	2.97	2.25	2.24	1.67	1.35	1.62	2.66	1.55	1.10	1.15	1.26
Sabatier	2.45	1.93	2.09	1.28	0.98	1.59	1.83	1.30	1.17	1.14	1.14
Schmidt	2.03	1.46	1.40	1.32	1.19	1.67	1.56	0.97	1.27	1.00	1.02
Shi	2.42	1.52	1.56	1.51	1.25	1.69	1.63	1.27	0.88	1.20	1.19
Symmans (I,II) + VDX	2.18	1.55	1.52	1.42	1.09	1.68	1.49	1.15	1.37	1.04	1.10
Symmans (III) + expO	2.34	1.34	1.47	1.34	0.98	1.54	1.47	1.35	1.47	1.29	1.23
UNT	2.05	1.58	1.57	1.63	1.06	1.69	1.36	1.36	1.23	1.56	1.43
VDX	2.55	1.83	1.80	1.61	1.36	1.90	1.49	1.23	1.29	1.21	1.21
BMI (median)	2.42	1.69	1.57	1.46	1.09	1.68	1.52	1.27	1.27	1.21	1.21
Nr. BMI $\geq$ 1.1	21	21	21	21	10	21	21	16	18	17	15
Nr. BMI $\geq$ 1.5	21	14	14	10	0	19	11	4	0	1	0

Table S9. Bimodality indices of individual modules (hgu133plus2 and hgu133a compendium). Wang et al. [8] characterize a distribution as being bimodal if the bimodality index (BMI) ≥ 1.1 and strongly bimodal if BMI ≥ 1.5. The first row indicates the various modules used to measure ER, HER2, PGR and proliferation (Additional file 1: Section 2). Proliferation was measured by the AURKA proliferation modules by Haibe-Kains et al. [6] (HK), Desmedt et al. [9] (D) and Wirapati et al. [10] (W) and the proliferation module (Proliferation) (Additional file 1: Section 2). BMI values are listed for each dataset from the Affymetrix compendium. The last three rows provide the median BMI value over all 21 datasets, the number of times the module was bimodal and the number of times the module was strongly bimodal, respectively.

Profile	ER	HER2	KI-67	PGR	5 Subtype STG	4 Subtype STG
1	ER-	HER2-	KI-67-	PGR-	basal	basal
2	ER-	HER2-	KI-67-	PGR+	luminal A	luminal A
3	ER-	HER2-	KI-67+	PGR-	basal	basal
4	ER-	HER2-	KI-67+	PGR+	luminal B (HER2-)	luminal B
5	ER-	HER2+	KI-67-	PGR-	HER2	HER2
6	ER-	HER2+	KI-67-	PGR+	luminal B (HER2+)	HER2
7	ER-	HER2+	KI-67+	PGR-	HER2	HER2
8	ER-	HER2+	KI-67+	PGR+	luminal B (HER2+)	HER2
9	ER+	HER2-	KI-67-	PGR-	luminal A	luminal A
10	ER+	HER2-	KI-67-	PGR+	luminal A	luminal A
11	ER+	HER2-	KI-67+	PGR-	luminal B (HER2-)	luminal B
12	ER+	HER2-	KI-67+	PGR+	luminal B (HER2-)	luminal B
13	ER+	HER2+	KI-67-	PGR-	luminal B (HER2+)	HER2
14	ER+	HER2+	KI-67-	PGR+	luminal B (HER2+)	HER2
15	ER+	HER2+	KI-67+	PGR-	luminal B (HER2+)	HER2
16	ER+	HER2+	KI-67+	PGR+	luminal B (HER2+)	HER2

A

Profile	ER	HER2	AURKA	SCM
i	ER-	HER2-	AURKA-	basal
ii	ER-	HER2-	AURKA+	basal
iii	ER-	HER2+	AURKA-	HER2
iv	ER-	HER2+	AURKA+	HER2
v	ER+	HER2-	AURKA-	luminal A
vi	ER+	HER2-	AURKA+	luminal B
vii	ER+	HER2+	AURKA-	HER2
viii	ER+	HER2+	AURKA+	HER2

B

Figure S7. Molecular taxonomy of the St. Gallen and SCM subtyping schemes. **A**) The St. Gallen subtype definitions [11] present a subtyping scheme based on the over(+)/under(-)expression of clinical markers for ER, HER2, KI-67 (proliferation status) and PGR. These four markers allow for $2^4 = 16$ distinct profiles. Each row corresponds to a particular profile, columns *ER*, *HER2*, *KI-67* and *PGR* indicate the over/underexpression status for each marker. For clarity, differences between groups are also highlighted by color. The column *5 Subtype STG* indicates the St. Gallen surrogate definitions of the intrinsic subtypes for each profile. In these, the luminal B subtype is subdivided into a luminal B (HER+) and luminal B (HER2-) subtype, while the HER2 subtype is associated with ER- profiles only. The column *4 Subtype STG* presents a mapping to the four main subtypes considered in this paper. Note that in this case we made the deliberate decision to map the luminal B (HER2-) profiles to the luminal B subtype, while the luminal B (HER2+) profiles were mapped to the HER2 subtype. This mapping was chosen in order to maximize similarity with SCMs as shown in Panel (**B**). SCMs do not consider PGR status and therefore lead to $2^3 = 8$ distinct profiles. For ease of comparison the profiles (rows) are ordered in the same way as the STG profiles. Assuming all processes are measured in the same way, in most cases, input vectors with identical ER, HER2 and proliferation status will be mapped to the same subtype. Discordance, however, may arise due to PGR status. For a sample with an identical ER, HER2 and proliferation profile, a luminal A or B subtype may be obtained for STGs (panel (**A**), profiles (2) and (4)), while for SCMs a basal subtype is obtained (panel (**B**), profiles (1) and (2)). Furthermore, the actual level of over/underexpression of a marker is relevant for SCMs, but not for STGs. This is likely to introduce additional discordance.

References

[1] Weigelt, B., Mackay, A., A'hern, R., Natrajan, R., Tan, D.S.P., Dowsett, M., Ashworth, A., Reis-Filho, J.S.: Breast cancer molecular profiling with single sample predictors: a retrospective analysis. Lancet Oncology **11**(4), 339–349 (2010)

[2] Weigelt, B., Mackay, A., A'hern, R., Natrajan, R., Tan, D.S.P., Dowsett, M., Ashworth, A., Reis-Filho, J.S.: Reflection and reaction, authors' reply breast cancer molecular profiling with single sample predictors: a retrospective analysis. Lancet Oncology **11**(8), 720–721 (2010)

[3] Sørlie, T., Borgan, E., Myhre, S., Vollan, H.K., Russnes, H., Zhao, X., Nilsen, G., Lingjærde, O.C., Børresen-Dale, A.L., Rødland, E.: The importance of gene-centring microarray data. Lancet Oncology **11**(8), 719–720 (2010)

[4] Natrajan, R., Weigelt, B., Mackay, A., Geyer, F.C., Grigoriadis, A., Tan, D.S.P., Jones, C., Lord, C.J., Vatcheva, R., Rodriguez-Pinilla, S.M., *et al.*: An integrative genomic and transcriptomic analysis reveals molecular pathways and networks regulated by copy number aberrations in basal-like, her2 and luminal cancers. Breast Cancer Research and Treatment **121**(3), 575–589 (2010)

[5] van de Vijver, M.J., He, Y.D., van't Veer, L.J., Dai, H., Hart, A.A.M., Voskuil, D.W., Schreiber, G.J., Peterse, J.L., Roberts, C., Marton, M.J., *et al.*: A gene-expression signature as a predictor of survival in breast cancer. New England Journal of Medicine **347**(25), 1999–2009 (2002)

[6] Haibe-Kains, B., Desmedt, C., Loi, S., Culhane, A.C., Bontempi, G., Quackenbush, J., Sotiriou, C.: A three-gene model to robustly identify breast cancer molecular subtypes. Journal of the National Cancer Institute **104**(4), 311–325 (2012)

[7] Guedj, .M., Marisa, .L., De Reynies, .A., Orsetti, .B., Schiappa, .R., Bibeau, .F., MacGrogan, .G., Lerebours, .F., Finetti, .P., Longy, .M., Bertheau, .P., *et al.*: A refined molecular taxonomy of breast cancer. Oncogene **31**(9), 1196–1206 (2012)

[8] Wang, J., Wen, S., Symmans, W.F., Pusztai, L., Coombes, K.R.: The bimodality index: a criterion for discovering and ranking bimodal signatures from cancer gene expression profiling data. Cancer Informatics **7**, 199–216 (2009)

[9] Desmedt, C., Haibe-Kains, B., Wirapati, P., Buyse, M., Larsimont, D., Bontempi, G., Delorenzi, M., Piccart, M., Sotiriou, C.: Biological processes associated with breast cancer clinical outcome depend on the molecular subtypes. Clinical Cancer Research **14**(16), 5158–5165 (2008)

[10] Wirapati, P., Sotiriou, C., Kunkel, S., Farmer, P., Pradervand, S., Haibe-Kains, B., Desmedt, C., *et al.*: Meta-analysis of gene expression profiles in breast cancer: toward a unified understanding of breast cancer subtyping and prognosis signatures. Breast Cancer Research **10**(4), 65 (2008)

[11] Goldhirsch, A., Wood, W., Coates, A., Gelber, R., Thürlimann, B., Senn, H.J., *et al.*: Strategies for subtypes - dealing with the diversity of breast cancer: highlights of the St Gallen International Expert Consensus on the Primary Therapy of Early Breast Cancer 2011. Annals of Oncology **22**(8), 1736–1747 (2011)
